# A systematic review of mobile device use in the primary school classroom and impact on pupil literacy and numeracy attainment: A systematic review

**DOI:** 10.1002/cl2.1417

**Published:** 2024-06-20

**Authors:** Claire Dorris, Karen Winter, Liam O'Hare, Edda Tandi Lwoga

**Affiliations:** ^1^ School of Social Sciences, Education and Social Work Queen's University Belfast Belfast UK; ^2^ School of Sociology, Social Policy and Social Work Queen's University Belfast Belfast UK; ^3^ Centre for Evidence and Social Innovation Queens University Belfast Belfast UK; ^4^ College of Business Education Dar es Salaam Tanzania

**Keywords:** children, curriculum, digital technology, education, educatonal technology, handheld devices, iPads, learning, literacy, meta‐analysis, mobile devices, mobile technology, numeracy, primary classroom, primary education, systematic review, teacher skills, technology integration

## Abstract

**Background:**

Investment in mobile devices to support primary or elementary education is increasing and must be informed by robust evidence to demonstrate impact. This systematic review of randomised controlled trials sought to identify the overall impact of mobile devices to support literacy and numeracy outcomes in mainstream primary classrooms.

**Objectives:**

The aim of this systematic review was to understand how mobile devices are used in primary/elementary education around the world, and in particular, determine how activities undertaken using mobile devices in the primary classroom might impact literacy and numeracy attainment for the pupils involved. Within this context, mobile devices are defined as tablets (including iPads and other branded devices), smartphones (usually those with a touchscreen interface and internet connectivity) and handheld games consoles (again usually with touchscreen and internet‐enabled). The interventions of interest were those aimed at improving literacy and/or numeracy for children aged 4–12 within the primary/elementary school (or equivalent) classroom.

Specifically, the review aimed to answer the following research questions:

‐ What is the effect of mobile device integration in the primary school classroom on children's literacy and numeracy outcomes?
‐ Are there specific devices which are more effective in supporting literacy and numeracy? (Tablets, smartphones, or handheld games consoles)
‐ Are there specific classroom integration activities which moderate effectiveness in supporting literacy and numeracy?
‐ Are there specific groups of children for whom mobile devices are more effective in supporting literacy and numeracy? (Across age group and gender).
‐ Do the benefits of mobile devices for learning last for any time beyond the study?
‐ What is the quality of available evidence on the use of mobile devices in primary/elementary education, and where is further research needed in this regard?

An Expert Advisory Group supported the review process at key stages to ensure relevance to current practice.

**Search Methods:**

The search strategy was designed to retrieve both published and unpublished literature, and incorporated relevant journal and other databases with a focus on education and social sciences. Robust electronic database searches were undertaken (12 databases, including APA PsychInfo, Web of Science, ERIC, British Education Index and others, and relevant government and other websites), as well as a hand‐search of relevant journals and conference proceedings. Contact was also made with prominent authors in the field to identify any ongoing or unpublished research. All searches and author contact took place between October and November 2020. The review team acknowledges that new studies will likely have emerged since and are not captured at this time. A further update to the review in the future is important and would build on the evidence reflected here.

**Selection Criteria:**

The review included children within mainstream primary/elementary/kindergarten education settings in any country (aged 4–12), and interventions or activities initiated within the primary school classroom (or global equivalent) that used mobile devices (including tablets, smartphones, or hand‐held gaming devices) to intentionally support literacy or numeracy learning. In terms of study design, only Randomised Controlled Trials were included in the review.

**Data Collection and Analysis:**

A total of 668 references were identified through a robust search strategy including published and unpublished literature. Following duplicate screening, 18 relevant studies, including 11,126 participants, 14 unique interventions, and 46 relevant outcome measures were synthesised using Robust Variance Estimation and a random effects meta‐analysis model. Risk of Bias assessment was undertaken by three reviewers using the ROB2 tool to assess the quality of studies, with 13 studies rated as having some concerns, and 5 as having high risk of bias. Qualitative data was also extracted and analysed in relation to the types of interventions included to allow a comparison of the key elements of each.

**Main Results:**

A positive, statistically significant combined effect was found (Cohen's *d* = 0.24, CI 0.0707 to 0.409, *p* < 0.01), demonstrating that in the studies and interventions included, children undertaking maths or literacy interventions using mobile devices achieved higher numeracy or literacy outcomes than those using an alternative device (e.g., a laptop or desktop computer) or no device (class activities as usual). However these results should be interpreted with caution given the risk of bias assessment noted above (5 studies rated high risk of bias and 13 rated as having some concerns). As the interventions and classroom circumstances differed quite widely, further research is needed to understand any potential impact more fully.

Sensitivity analysis aimed to identify moderating factors including age or gender, screen size, frequency/dosage of intervention exposure, and programme implementation features/activities (based on Puentedura's [2009] SAMR model of technology integration). There were too few studies identified to support quantitative analysis of sufficient power to draw robust conclusions on moderating factors, and insufficient data to determine impact beyond immediate post‐test period. Sensitivty analysis was also undertaken to exclude the five studies identified as having a high risk of bias, to identify any impact they may have on overall findings.

**Authors' Conclusions:**

Overall, this review demonstrates that for the specific interventions and studies included, mobile device use in the classroom led to a significant, positive effect on literacy and numeracy outcomes for the children involved, bringing positive implications for their continued use in primary education. However given the concerns on risk of bias assessment reported above, the differing circumstances, interventions and treatment conditions and intensities, the findings must be interpreted with caution. The review also supports the need for further robust research to better understand what works, under what circumstances, and for whom, in the use of mobile devices to support learning.

## 1 PLAIN LANGUAGE SUMMARY

Mobile devices in the primary school classroom may improve literacy and numeracy learning, though concerns about risk of bias and uncertainty about modes of effect limit conclusions.

### 1.1 What is this review about?

This review gathered evidence on how mobile devices (including tablets, mobile phones, and handheld digital games) are used in primary school classrooms to help children's literacy and numeracy skills. An Expert Advisory Group supported the process to help findings relate to everyday practice.

### 1.2 What did the review hope to find out?

The review aimed to assess the impact of mobile devices in primary school classrooms on children's literacy and numeracy achievement, and its methodological quality.

The secondary objectives were to assess whether some devices or classroom activities were more effective than others in supporting literacy and numeracy; whether some children benefitted more from these devices (e.g., across age or gender); and whether effects lasted beyond the duration of the studies.

### 1.3 How were studies identified for inclusion in the review?

Between October and November, 2020, searches were conducted across electronic databases, journals, web pages and other sources to identify all relevant studies. Randomised Controlled Trials (a robust experimental design) were included. Children in the studies must have been between the age of 4 and 12, with research taking place in a primary/elementary school classroom (or equivalent grade in other countries). The children must have used the mobile devices themselves, rather than the teacher, to support their learning. The studies must have measured literacy or numeracy learning outcomes.

### 1.4 What studies are included in the review?

A total of 18 relevant studies, incorporating 14 unique mobile device interventions, and 46 relevant outcome measures were included in the review. The studies were from across six countries (USA, Netherlands, UK, Malawi, Turkey, and Cambodia), and included 11,126 participants. Five studies considered literacy, 11 considered numeracy, and 2 included both subjects. The duration of interventions ranged from 1 to 120 h.

### 1.5 What did the review find out?

Children had better results in maths or literacy tests when they used mobile devices for their lessons compared to children who used an alternative device (e.g., a laptop or desktop computer) or no device (class activities as usual). These results must be interpreted in light of overall concerns about methodologic quality, since 5 studies were at high risk of bias and the remaining 13 studies were moderate risk of bias.

There were too few studies to answer the secondary questions about why the mobile devices worked and for whom, and insufficient information provided to identify whether the benefits of mobile devices lasted for any time beyond the study.

### 1.6 What do the findings of the review mean?

**For researchers:** This review highlights that while mobile devices can support literacy and numeracy learning, we do not currently know enough about how they work best, what makes them effective, and how teachers can best use them in lessons. Further research will help to better understand this. Future research should also pay closer attention to minimising risk of bias and to how and when mobile devices are actually used in real life, so that their research reflects real activities.

**For teachers and other educational professionals:** We know that mobile devices overall can help children to learn better, however we are uncertain about what types of devices or strategies work better. Teachers should think carefully about the ways in which they are using mobile devices, how they are using them alongside other teaching activities and approaches, and how they might add value to children's learning experience.

**For the design of educational interventions:** The design and development of educational interventions should be based on evidence, therefore those designing such interventions should pay close attention to existing research, while also investing in new research. Any new interventions should be evaluated with rigorous methods to minimise risk of bias and show that they are making a difference to learning.

### 1.7 How up to date is this review?

The review includes research up to November 2020. It is important to repeat this review again as new research emerges, as practice is changing quickly.

## 2 BACKGROUND

The world is changing rapidly, in part due to the advances in technology that once amazed and now are often taken for granted. Perhaps the most significant advancement is the emergence and development of the internet, or World Wide Web. The depth of global impact that the internet has had right across our lives would have been difficult to predict, including the impact on consumer behaviours (Voramontri & Klieb, 2019), teaching and learning (Dockerty, 2019; Gamliel, 2014; Wastiau et al., 2013), and social networking (Van Deursen & Helsper, 2018; Webster et al., 2021). As adults, it has changed our lives, with infinite knowledge and opportunity just a click of our smartphone away. Yet today's generation of children have no experience of a pre‐technology world. Our vocabulary has expanded in response; Prensky (2001) calls these children Digital Natives; Twenge (2017) describes post‐millennials as the iGeneration; while Leathers et al. (2013) coined the term Digitods for toddlers who can navigate a swipe‐screen before they can talk.

As technology has advanced, its applicability to education has been explored and new devices and interventions developed. This has brought a world of possibility for creative and innovative educational experiences for the iGeneration, inside and outside the classroom (Hsin et al., 2014) and forced a reimagining of pedagogical practices (Fleer, 2011). Mobile devices are commonplace in the classroom in developed countries, and emerging in less developed countries. Yet this is an area where research has not kept pace with the development and adoption of technology (see e.g., Crompton et al., 2017; Herodotou, 2018), and while the potential for learning is evident, the impact that mobile devices actually have on educational outcomes remains unclear. Set within a backdrop of wider austerity, and with the unprecedented challenges for education that a global pandemic has brought, it is critical that investment is made in the most effective tools and approaches to best support educational outcomes. This is not to suggest that technology can or should replace traditional teaching methods, or that it will be relevant to every child, subject or setting. This review is therefore undertaken with this acknowledgement in mind. The assessment of theories and evidence below considers potential application, as and when appropriate, to supplement educational practice. A strong evidence base to demonstrate when technology can be of benefit, and indeed when it is not relevant or beneficial, should further inform any investment.

### 2.1 Description of the condition

This review is focused specifically on primary or elementary school education (generally including children between the ages of 4 and 12), rather than the full educational age spectrum, and considers literacy and numeracy education rather than the wider primary curriculum. This decision was informed by the differences in primary and post‐primary education, and the use of technology within these in terms of the subjects studied, the approach to pedagogy, and activities undertaken. There is also a central focus on literacy and numeracy. These are core skills which equip a child to engage in the wider curriculum and have far‐reaching implications across the life‐course. Research by the National Literacy Trust (Clark & Teravainen‐Goff, 2018) shows that children who are more engaged with literacy have better mental wellbeing, while Gross et al. (2009) note the long‐term costs to the public purse to address the impact of poor literacy and numeracy. The 2018 PISA tests (OECD, 2019), designed to assess reading, science, and maths skills of 15‐year‐olds across the globe, show the UK moving up the rankings in maths (18th, up from 27th in 2016) and in reading (14th, up from 22nd in 2016), yet still falling below many other countries. A focus on effective literacy and numeracy education is therefore priority for primary age children and a valuable focus of this review.

The review was also informed by a child rights perspective. While the UN Convention on the Rights of the Child (UNCRC, 1989) was conceived before the digital world as we know it, existing Articles must be reinterpreted to reflect the changing circumstances of children's lives. Livingstone et al. (2016, p. 18) provides a useful summary of children's rights in a digital world, highlighting the ‘three Ps’ which include a right to *protection* from threats; a right to *provision* of the resources necessary for development to full potential, and a right to *participation* in processes which support development and engagement as an active part in society. Stoilova et al. (2020) reflect that the onus is now on governments and organisations to develop legislation and safeguards to ensure that all children can realise their rights in the online world, not just to stay safe, but to explore and actively engage with the opportunities that the digital world brings. Any discussion about the increased use of technology must be viewed alongside a wider discussion on online safety, the challenges faced in keeping children and young people safe online, while supporting their rights to make use of technology. Many have written on this comprehensively and in more detail than is possible in this review, and a range of resources are available to support teaching and learning with digital devices. The UK Council for Internet Safety (2020) has also developed ‘Education for a connected world’, a guidance document for anyone working with children and young people and featuring key messages and responsibilities for safe use of technology. While online safety is not presented in detail in this review, nor the safety implications of individual interventions considered, it is implicit that schools must consider online safety within wider policies and procedures, alongside increased technology use in the classroom.

In summary, this systematic review and meta‐analysis sought to understand how activities undertaken using mobile devices in the primary classroom might impact literacy and numeracy attainment for the pupils involved. The interventions of interest are those aimed at improving literacy and/or numeracy for children aged 4–12 within the primary/elementary school (or equivalent) classroom. This is further expanded on below.

### 2.2 Description of the intervention

**Figure 1:**
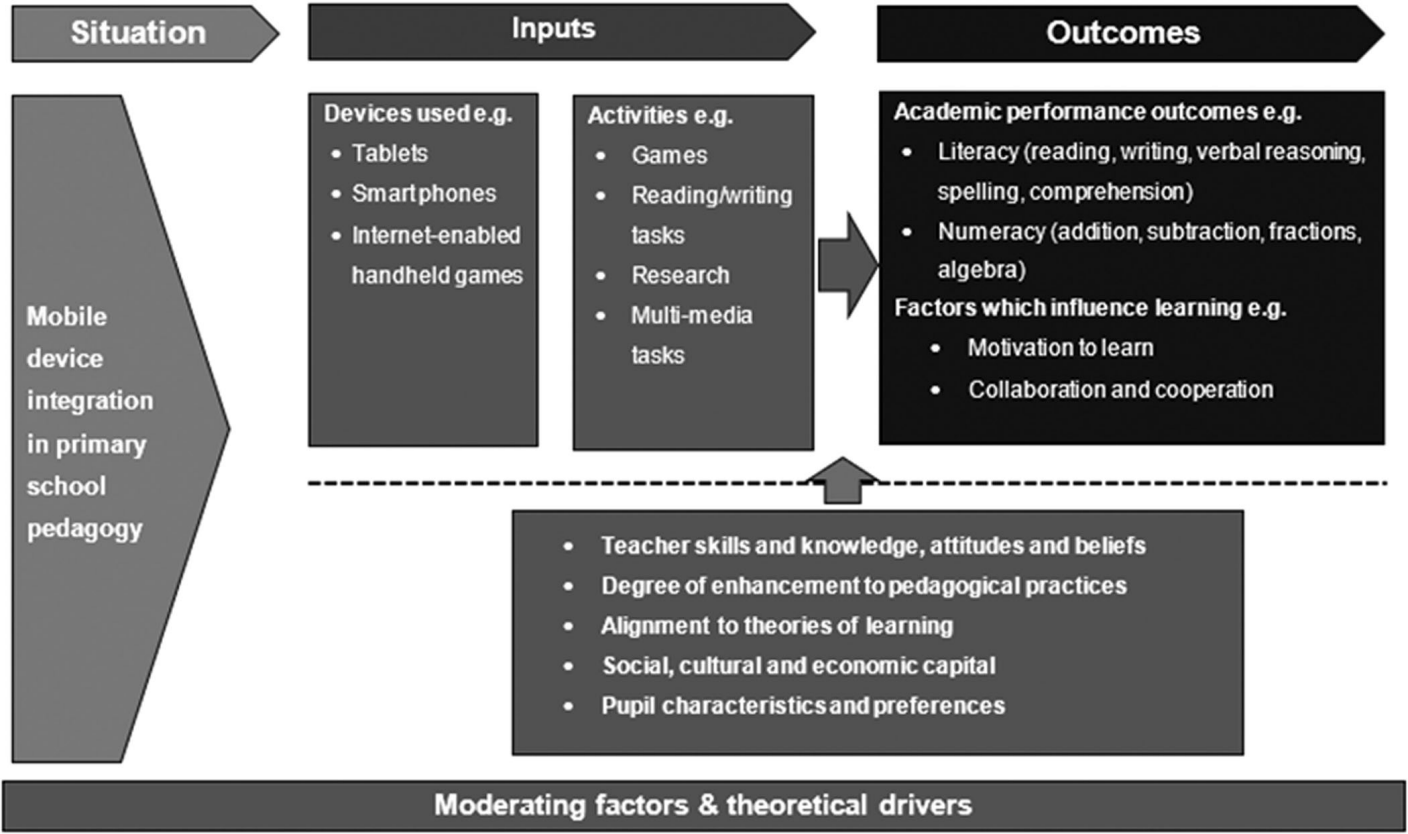
Logic model.

In developing this systematic review, it was useful to represent the emerging themes as a theory of change, demonstrating how the elements work together to contribute to improved outcomes for children. Within this theory of change (Figure 1), four elements are considered in how mobile technology is used in the primary school classroom. These are (a) devices; (b) activities and interventions; (c) outcomes; and (d) moderating factors and theoretical drivers.

#### 2.2.1 Mobile devices

Mobile devices commonly used by children include tablets, smart‐phones, and gaming devices. In 2018, the Office for National Statistics reported that for the first time, 100% of households with children across England, Scotland and Wales had internet access. OFCOM (2022) presents a picture of everyday technology usage for children across the UK. Device ownership begins early; 17% of 3‐ to 4‐year‐olds are reported to own a mobile phone, rising to 91% by the age of 11 and 100% by the age of 15. Children spend a significant amount of time online, with 97% of 5‐ to 15‐year‐olds averaging 20.5 h per week (OFCOM, 2021). Activities include game‐playing, uploading/viewing media, social networking and listening to music. For most children in the developed world, the incredible opportunities of the digital world are no further than an arm's length away, day or night, with Weinstein (2018, p. 3598) noting that social media is ‘intertwined with daily life’.

Despite the widespread ownership and engagement of technology, patterns of usage differ from child to child, and also change with time. OFCOM (2022) reported girls spending on average longer on their phones than boys, while boys spent longer playing games online; however, the gap is closing year on year, with online games becoming increasingly popular among girls. Boys were also more likely to report using online games as a means of connecting with their friends. Mascheroni and Olafsson (2014) noted that the more creative and skilled activities, such as blogs, publishing videos or photos (content creation), were undertaken by fewer children overall, however the latest OFCOM (2022) report found that content creation is now a daily activity for most young people. This represents a shift from consumer to contributor of information online.

Since March 2020, a pandemic has caused unprecedented disruption worldwide, necessitating behavioural changes as we adapt to new ways of communicating, working, and playing. Over the past 2 years there has been an increase in the amount of time spent online, and the types of activities undertaken, and research on the implications will be emerging for some time to come. The OFCOM (2022) report notes an overall increase in online activity for children in the UK during lockdown, however found that primary‐aged children were less likely to have adequate access to an appropriate device to undertake online schooling activities than secondary‐aged children.

**Devices in education:** Various sources suggest mobile devices are rapidly increasing in popularity in schools, with significant investment due to the potential for transformative pedagogy that they represent (Crompton et al., 2017; Nikolić et al., 2019). Research by the British Educational Suppliers Association (BESA, 2015) found that from a sample of 335 UK primary schools, 71% reported using tablets in the classroom, representing a significant increase from the previous year. Mobile devices have many benefits, including portability, the ability to customise, and comparative affordability (Haßler et al., 2015). Emerging research is beginning to show how they can be used to change classroom practice, including increasing motivation (Ciampa, 2014), classroom interaction (Campbell & Jane, 2012), and improving educational outcomes (Herodotou, 2018; Looi et al., 2015). The intuitive design of tablets and smartphones, coupled with affordability and the potential to ‘bring your own’, make them ideally placed to influence traditional teaching methods, and this undoubtedly presents an opportunity for schools. The NMC Horizon Report K‐12 Edition (Johnson et al., 2014) identified such ‘intuitive technology’ as having the potential to significantly impact educational practice over the coming years.

In the classroom, mobile devices may be accessed individually (a ‘one to one’ approach) or shared between groups of pupils. Burke and Hughes (2018) argue that due to the personalised nature of mobile devices in particular, one device per child maximises their effectiveness in the classroom. While one tablet per child in every school, even in developed countries, is currently far from possible, another option has emerged. As ownership of smartphones and tablets increases among children, schools are now considering how they can use these in class (see e.g., Rae et al., 2017). Such ‘bring your own device’ approaches also bring challenges, with online safety, appropriate behaviour policies, infrastructure capacity and ensuring equality of access, only some of the necessary considerations.

#### 2.2.2 Activities and interventions

Digital technology alone does not improve learning or transform education (Facer & Selwyn, 2021). Rather, the important elements are the activities or interventions, and how they complement traditional learning. Mobile devices can be used across all subjects, and indeed, many countries have technology embedded as a requirement within their curricula across all stages of schooling, both to support the development of specific technology skills, and to support wider learning. Activities undertaken via mobile devices either directly access the internet or use device Applications (‘Apps’) or inbuilt device functions. Educational Apps are one of the more popular categories across the main App stores, with downloads reaching millions and growing, particularly since lockdown (Papadakis, 2021). Table 1 below summarises some popular educational Apps and educational websites (hereafter referred to as interventions) available, demonstrating the wide variety of activities involved.

**Table 1:** Examples of mobile device interventions.

Intervention	Area of study	Summary of intervention
Motion Math	Maths	The Motion Math model includes multiple levels of mathematics content, aimed at children aged 4–11 and covering general arithmetic concepts aligned to the school curriculum, including fractions, addition, subtraction, and percentages. Pupils can work independently at their own pace. The ‘tilt’ facility on mobile devices allows children to physically manipulate on‐screen graphics, e.g., directing a falling star to a slot. The game‐based intervention facilitates formative assessment through tracking performance, direct feedback to the child, and increasing difficulty when answers are correct. Teachers receive feedback on pupil usage and performance.
Mathletics	Maths	A learning platform designed for use in schools and aligned to the UK primary school curriculum, however, can also be used at home. Activities can be accessed via tablet or desktop computer and include a range of tutorials and interactive games. A test option is available, and pupil activities are marked automatically with detailed reports provided for the teacher. There is also a facility to assign homework. Activities incorporate challenges to motivate individual pupils, with points awarded for completion. Mathletics also includes scheduling and customisation facilities for teachers to support planning.
Learning Bug Club	Literacy	A phonics‐based reading programme which can be accessed online on all devices. eBooks, aligned learning activities and comprehension quizzes are matched to the curriculum and to individual children's skill level, allowing them to work at their own pace. Books and reading materials cover a range of fiction and non‐fiction topics to meet pupil interests, and a ‘read aloud’ function supports those who are struggling. Children can continue with their reading at home or school by logging in to their ‘My Stuff’ area, and rewards can be earned for completed activities.

The types of activities undertaken via digital interventions vary widely, for example, basic reading and writing activities, playing games, watching instructional videos, researching topics of interest, completing online tests, or taking photographs or videos. The range of activities is as vast as the subjects covered. However, there are several evident commonalities. Play has a central role in the early years and primary/elementary education curricula, with a widely established body of research showing the effectiveness of play‐based learning, from free play through to instructive games (Whitebread et al., 2012). Most educational digital interventions aim to make learning more fun, creating a positive attitude towards a subject and aiming to enhance enjoyment of learning).

The screen size is an important difference between tablets (usually between 7 and 13 inches) and mobile phones or games consoles (usually smaller). There is limited research on the differences in educational value for each, however a recent study by Haverkamp et al. (2023) found that the reading experience of university students using a tablet was more positive than when using a mobile phone. Given the discussion above on growing mobile phone ownership in children, and their potential use in education, it is useful to consider if screen size makes interventions more effective, or changes the learning experience in any way. Screen size is therefore considered in the meta‐analysis below. However, any comparison is undertaken with the assumption that a much more detailed understanding of the differences in functionality is necessary to draw conclusions.

Within literacy and numeracy, the range of mobile device interventions is also broad, reflecting the core subject elements, for example, phonics, spelling, grammar or comprehension. Table 2 provides further examples of how digital interventions may support learning. The next secton also considers the literacy and numeracy learning outcomes which these interventions seek to support.

**Table 2:** Examples of how elements of literacy and numeracy are addressed in mobile device interventions.

Subject	Components	Activity examples in the research
Literacy	**Sounds, words and reading**	**eBook:** standard ereader with audio narration to support vocabulary recognition (Lee, 2017). ***Letter Works:*** a tablet app replicating magnetic letters on a virtual board, which children can manipulate to spell words (D'Agostino et al., 2016).
Literacy	**Comprehension**	***Comprehension Booster:*** an online reading app with accompanying explanatory images, the option to have words read aloud, followed by comprehension questions to assess understanding. (Horne, 2017). *eBook reader* with option to add notes to summarise the key points & demonstrate understanding (Union et al., 2015).
Literacy	**Writing**	***Comics Head Lite:*** a ‘create your own comic strip’ app to create stories (Moon, Wold and Francom, 2017). ***Popplet:*** an online concept mapping tool to plan and develop essay ideas (Kervin & Mantei, 2016).
Numeracy	**Number recognition & simple counting**	***Building Blocks Programme:*** 200 games to introduce shapes, patterns and numbers (Foster et al., 2016). ***Knowledge Battle:*** an educational videogame‐style intervention, presenting basic maths knowledge through fun characters and storylines (Hieftje et al., 2017).
Numeracy	**Mathematical operators**	***Sacar10:*** An online maths programme presenting sums and mathematical challenges in game format (Zaldívar‐Colado et al., 2017). ***Catch the Monster**:* Online game‐based activities to support understanding of fractions, featuring digital number lines (Fazio et al., 2016).
Numeracy	**Mathematical reasoning**	***OpenSim***: An opportunity to trial maths concepts that come up in everyday life via a Virtual Reality environment (Kim & Ke, 2017). ***CoSy_World***: A 3D virtual environment with maths problems and challenges to undertake to move from scene to scene (Bouta & Retalis, 2013).

### 2.3 How the intervention might work

#### 2.3.1 Aligning mobile device use in education with theories of learning

In considering how such educational interventions might work, it is useful to consider how their features may align with traditional pedagogy, and in particular theories of learning. A summary of key learning theories and reflection on how new educational technologies, and in particular mobile devices, align to these theories, is set out in Table 3 below.

**Table 3:** Summary of relevant theories of learning and their application to mobile interventions.

Theory	Notable influencers	Key features	Influence in mobile device interventions
**Behaviourism**	John B. Watson, B.F. Skinner, Ivan Pavlov	Classical and operant conditioning, where learning is encouraged through reward and punishment, repetitive learning and stimulus feedback.	Rote‐learning and repetitive activities, practice games, basic feedback through right/wrong sounds or symbols, advancing to the next level when a required standard or score is achieved.
**Cognitive Learning Theory and Constructivism**	Jean Piaget Carl Rogers	Active rather than passive learning, involving interaction with surroundings, physical manipulation of objects to learn about their properties, and interpretation of observations based on existing knowledge. Child‐centred, hands‐on and creative learning, with the teacher as facilitator rather than instructor. Learning through development of relationships and engagement with others, collectively making sense of the surroundings.	Manipulative learning through touchscreen function, allowing children to explore shapes and object properties; child‐centred learning with the child working through activities at own pace; engaging technology providing opportunities for creativity and investigation, either alone or in groups.
**Social constructivism (or socio‐cultural approach)**	Lev Vygotsky, Jerome Bruner	As above, active learning and teaching, however learning is a two‐way process involving collaboration with peers and teachers. Modelling behaviour supports learning (scaffolding), and positioning learning within the Zone of Proximal Development.	Interventions which include instructional elements (virtual scaffolding), the opportunity to review these and repeat until competent, collaborative opportunities, detailed feedback to teacher to allow appropriate intervention.

Modern pedagogy reflects a range of learning theories and approaches, each of which incorporate elements which can be integrated within mobile device intervention design to better support learning. The independent and self‐directed nature of learning, which well‐designed mobile devices can offer, places the child at the centre of their own learning, empowering them to think critically in decision‐making (Woloshyn et al., 2017; Wong & Lung‐Hsaing, 2013). They can also provide individualised learning experiences to support each child's unique approach to learning. However, the role of social learning, collaboration and shared enquiry is less obvious within existing mobile interventions, particularly where mobile devices are used alone, and should be supported through opportunities to engage with peers during the activities.

Mobile device interventions also provide opportunity for formative assessment (Mitten et al., 2017), which Higgins (2014) proposes should lead to action or change, either for the teacher or the learner. However, effective feedback must move beyond ‘right or wrong’, instead supporting understanding of why and what lessons can be learned. If used appropriately, digital devices in the classroom can provide individualised and specific feedback on progress directly to pupils, while bringing additional opportunities for teachers to review and assess pupil progress in real time and offer targeted feedback to support learning (Dalby & Swan, 2019).

Hirsh‐Pasek et al. (2015), Kucirkova (2017) and others have highlighted the need to develop a framework to guide the quality, design and content of commercially available educational Apps, as these are currently largely unregulated. Kucirkova (2017) also highlights the lack of input from teachers and other educational professionals in their design. Coproduction, she notes would increase both the quality and applicability of Apps, and their uptake by teachers.

#### 2.3.2 Outcomes for children

Within education, research on the impact of technology on pupil outcomes usually focuses on either academic achievement (primary outcomes) or factors that influence learning (moderating factors). Within literacy and numeracy, primary outcomes may include reading or writing fluency (e.g., Wu & Gadke, 2017), or accuracy (number of sums correct) in a math test (Musti‐Rao & Plati, 2015). Moderating factors may impact the teaching environment or pupils' learning experience, for example, motivation to learn (Turan & Seker, 2018), enjoyment of lessons (Moon, Wold and Francom, 2017) or opportunities to better collaborate with classmates (Davidsen and Vanderlinde, 2016).

##### Primary outcomes

Within education, primary outcomes for children refer primarily to their academic achievement across subjects studied. Examples include reading fluency and comprehension in literacy; number recognition or application of operators (addition, subtraction, multiplication, division) in maths; or ability to recognise patterns and classifications in science. Primary outcome measures generally include standardised tests, or researcher‐developed assessments. Standardised measures are available for many common academic subjects, and benefit from the availability of a ‘norm’ against which individual scores can be compared, as well as standardised procedures for administration and scoring (Morris, 2011). Examples include the Stanford‐Binet Intelligence Test (and other similar tests) which provides an overall measure of ability; or the New Group Reading Test (GL Assessment) which provides a reading age and ‘standard age’ score. The statutory educational assessments of a country are usually standardised and often used in research of this nature.

Where a standardised measure does not exist, is expensive or requires expertise to administer, the researcher may develop their own tool, such as a test based on curriculum content, or a marking scheme developed to assess content of a piece of work. Researcher‐developed measures can lack the validity of standardised tests; however validity can be assessed, for example, in comparing correlation with a standardised tool (as demonstrated by Proctor, 2011). Often, a combination of standardised and researcher‐developed measures may be most appropriate.

The Education Endowment Foundation (2019) reports extensive evidence of ‘moderate learning gains’ when technology is integrated in teaching across a wide range of subjects and age groups, resulting in an additional 4 months' progress on average (EEF ‘toolkit for teaching and learning’ calculations based on impact, cost and strength of evidence). However, EEF conclusions suggest that the type of technology, and the way in which it is integrated within the classroom, vary widely. A brief look at studies on the impact of technology on literacy or numeracy reveals a broad spectrum of interventions and findings, significant and non‐significant. Simms et al. (2019) undertook a systematic review of interventions to support mathematical achievement in primary school children. While the scope of the review incorporated all maths interventions, the authors identified 42 (from a total of 80) interventions which required technology to engage children in numeracy learning. The format of these activities varied widely; in some cases, children were engaged directly in virtual environments, others involved playing online games, while others used technology only as a small part of the activity, for example, using a digital pen to undertake maths exercises. Individually, the studies identified showed a range of significant and non‐significant effects, and due to the variety of interventions, Simms et al. (2019) were unable to undertake meta‐analysis to demonstrate an overall effect size. However, there are clear lessons from the overall review, notably that while the delivery mechanism plays a role, we must also look beyond this to understand the theory and strategies at play within the intervention. Meanwhile, Talan (2020) undertook meta‐analysis of studies using mobile learning across all subjects and grade levels and found an effect size of −0.015 for maths interventions, representing the smallest impact across all subjects. The findings therefore reveal a lack of consistency.

Cheung and Slavin (2012) also conducted a systematic review and meta‐analysis of education technology to support reading. From a total of 85 studies, they found that technology had a small, positive effect on literacy in comparison to ‘normal’ activities. However as with their study above, this was undertaken before mobile device development, therefore represents only traditional technology (computers and interactive whiteboards). The study included pupils from 5 to 18 years, and reports differing effects, with higher learning gains for older pupils – in contrast to the same authors' study on maths achievement above. The authors note the wide range of interventions and varying effects between studies, concluding that more research is needed to better understand the overall impact and how the interventions can be most effectively used.

More recently, Tingir et al. (2017) undertook a meta‐analysis of mobile device use across grades K‐12 (aged 5–18) and incorporating all subject areas. Due to limited search scope, only 14 studies were identified, 3 of which included reading interventions. Sub‐group analysis revealed interventions for reading to be significantly more effective than other subjects, however results should be interpreted with caution given the small number of studies included. Most recently, Savva et al. (2022) undertook meta‐analysis to examine the effects of electronic storybooks on language and literacy outcomes for children aged 3 to 8. While reporting a small, positive effect, the authors also discuss the extricability of the device from learning theory and teaching approaches and features. In particular, they reflect on the role of adult scaffolding and the potential effectiveness of device features which seek to replicate this. Commonly across all such studies, blanket conclusions on the effectiveness of technology versus traditional teaching methods are not prudent due to the complex nature of both the subject area and the intervention features.

As already noted, the use of technology is not always going to be relevant to a class, subject or situation, and other teaching approaches, tools and methods will be more appropriate. However, where relevant, technology has the potential to impact on a wide‐ranging set of primary outcomes, depending on how it is used. As demonstrated above, the actual impact can also vary widely; in relation to literacy and numeracy, this is explored further through this systematic review.

##### Moderating factors

A moderating factor or variable refers to the situation when the relationship between two variables (in this case, mobile device use and academic outcome) is influenced (moderated) by a third variable (e.g., motivation to learn). The teaching environment across each school differs, as do individual child interests, abilities and behaviours – each of these introduces a wide range of potential moderating variables, which may have positive or negative impact on the desired primary outcome. OECD (2015) found that those countries reporting heavy investment in technology in schools demonstrated no significant improvement in reading, writing or maths. While mobile device usage in the classroom continues to grow, effectiveness remains unclear. Therefore, a closer look at the factors which may moderate impact is prudent. These include increased collaboration, inclusion and motivation to learn, as well as teacher skills, attitudes and approaches.

**Collaboration and inclusion:** Clark and Abbott (2015) evaluated iPad implementation in a primary school in Northern Ireland, situated in the 10th most deprived area, and the first school in the region to provide one‐to‐one tablets for pupils. They found increased inclusion and collaboration, observing that children with additional needs were able to join in with activities on the tablets, and previously observed gender differences in subject areas decreased. Overall, children's interest was sustained, and ownership of learning increased.

Burke and Hughes (2018) studied the integration of touchscreen technology in the curriculum in Canada, with a particular focus on students with diverse abilities, and found that such technology in the classroom can be transformative where children have previously had difficulty engaging with traditional teaching methods. Technology has also been shown to support a reduction in gender inequalities in education. Clark and Abbott (2015) reported that boys' motivation often increased with the use of iPads in lessons, while McQuillan and O'Neill (2009) discuss how the embedding of technology in the curriculum from an early age has narrowed the gap in technology skills between boys and girls and has had a positive impact on girls' participation in STEM subjects.

**Motivation to learn:** Self Determination Theory (Deci & Ryan, 1985) offers a distinction between extrinsic and intrinsic motivation and has been influential in pedagogy. Extrinsic motivation is driven by external factors, such as a fear of getting into trouble, or the promise of a reward. Intrinsic motivation stems from internal factors, such as a sense of achievement, personal challenge, or ‘purely for the enjoyment of the activity in itself’ (Ryan & Deci, 2000). Researchers have shown that intrinsic motivation is a stronger determinant of engagement in the classroom (see e.g., Richter, 2016, Taylor et al., 2014). Malone and Lepper (1987) propose a ‘taxonomy of intrinsic motivations for learning’. The task should be *challenging* in a way that is neither too boring or too difficult, allowing learners to select their own level of ability and work at their own pace. Tasks should *stimulate curiosity*, in both sensory and cognitive ways, for example, through sounds, pictures, and actions. The learner should feel *in control of the task*, with the ability to make independent choices and control the direction of activities themselves. Opportunities for *cooperation and collaboration* increase intrinsic motivation through increased social competence, the realisation of common goals and the opportunity to learn from and support one another. Finally, the task should provide an *element of competition*, with others or with oneself. These elements have important implications for the design of educational interventions.

**Teacher attitudes and beliefs:** Kagan (1992) proposed that teachers screen any new knowledge through a filter of existing pedagogical beliefs. Those who do not feel adequately skilled in the use of mobile devices and their applicability to pedagogy, or do not feel positively towards the potential of educational technology, may be unwilling to use them. A systematic review by Tondeur et al. (2017), found that pre‐existing pedagogical beliefs can be a barrier to technology integration. Choy and Ng (2015) further support this view, citing studies which demonstrate that despite availability of technology in many schools, teachers who view technology less favourably are less likely to use it in a transformative manner, when transformation is desirable and relevant in the context. However, Matzen and Edmunds (2007) found that the relationship between pedagogical beliefs and technology use is bi‐directional, with technology also having the power to change pedagogical beliefs over time. Indeed, Burden et al. (2012) found that mobile devices forced teachers to rethink their role in the classroom, changing the way they relate to their students and helping them work more collaboratively. Long‐held beliefs are the hardest to change, while more recently formed beliefs can be more easily influenced. Professional teacher development can therefore support behaviour change in this regard if effort and focus is placed on understanding and changing these beliefs.

Positive leadership is also critical. Before mobile technology, Matzen and Edmunds (2007) reflected on how the wider school context, culture and resources could support or hinder technology integration, positing that a whole school approach is necessary for technology implementation to be transformative. Choy and Ng (2015) note that the school culture and infrastructure can impact individual teacher attitudes and suggest that a school principal with a positive attitude to technology, coupled with a clear school vision, strong communication and, of course, access to technological tools, increases the chances of teacher ‘buy‐in’.

**Figure 2:**
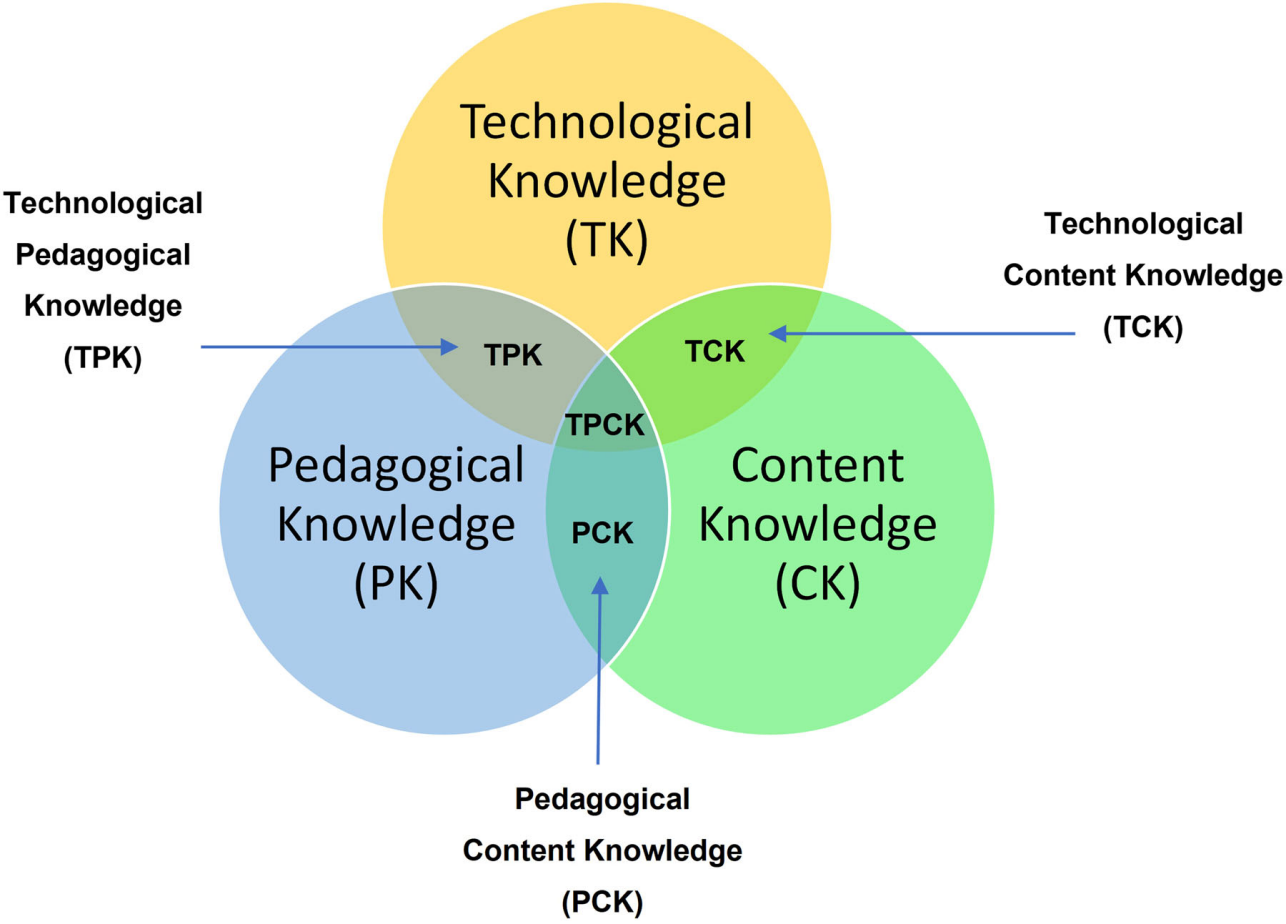
The TPACK framework.

**Teacher skills and knowledge:** Access to mobile devices, and availability of well‐designed applications which mirror the theories of learning and motivation discussed above do not automatically translate to improved outcomes for pupils. When their use has been identified as relevant, the way in which these devices are used to support learning is key. The TPACK Framework (Technological, Pedagogical and Content Knowledge) (Mishra & Koehler, 2006) is a commonly cited model within the literature on teacher's implementation of technology in the classroom (see e.g., Dewi et al., 2021; Santos & Castro, 2021; Voogt & McKennyey, 2017) therefore useful to consider in further detail. The TPACK Framework (Figure 2) adds a technology filter to the Shulman (1986) theory of pedagogical content knowledge, which has been influential in teacher education and development (Berry, 2008).

The TPACK Framework identifies the knowledge components necessary for a teacher to effectively integrate technology in the classroom. These include *content knowledge* of their specific subject area; *pedagogical knowledge* of general approaches to teaching; and *technological knowledge* of the equipment and Applications. The model considers the intersection of these three elements of knowledge, theorising that for successful and effective implementation, all three must be present, and importantly, combined in the classroom.

Many researchers have identified teachers' lack of appropriate pedagogical knowledge, and/or their understanding of how to combine it with their content and pedagogical knowledge, as a barrier to technology implementation (Kearney et al., 2018; Voogt & McKennyey, 2017). Indeed, Burke and Hughes (2018) suggest the biggest barrier to technology implementation lies in a lack of teacher training and support to keep up to date with advancements. Building and sustaining teacher skills and knowledge through continued professional development, and supporting a positive attitude to technology, must therefore be prioritised when embedding mobile technology in the classroom (Zipke, 2018).

**Degree of enhancement to pedagogical practices:** A further critical implementation theory focuses on the degree to which mobile devices are used to enhance, rather than duplicate, current practices. The SAMR model (Substitution, Augmentation, Modification, and Redefinition) (Puentedura, 2006) receives prominent focus (e.g., Crompton & Burke, 2020; Keane et al., 2016; Savignano, 2018). The model considers the degree to which technology can change what was previously possible in the classroom. Table 4 below describes the four levels of technology integration defined by Puentedura, and additionally provides an example of classroom activities at each level.

**Table 4:** Stages of the SAMR Model, Puentedura (2006).

Implementation stage	Description	Example of activity
***Substitution***	Technology acts as a direct substitute, with no functional change	An online book is used in place of a paper copy of the same book. There are no additional pictures or content included in the online version.
***Augmentation***	Technology acts as a direct substitute, with functional improvement	A computer word processor is used to write an assignment, allowing for a more creative presentation such as the inclusion of pictures or diagrams, while the ability to edit documents makes the drafting process easier.
***Modification***	Technology allows for significant task redesign	Pupils use the internet to undertake independent research to inform their assignment. This has the potential to expand their knowledge, while also supporting the development of new skills.
***Redefinition***	Technology allows for the creation of new tasks, previously inconceivable	A multi‐media assignment is undertaken, with pupils using video, audio recordings and other creative tools to develop their assignment, and then share this with peers via a class blog.

Substitution and augmentation are both considered to *enhance* pedagogy; the usual activities are undertaken, however there may be some additional function. Modification and redefinition are both considered to *transform* pedagogy; technology makes it possible for new and creative activities to be undertaken, therefore adding to the existing learning experience. Puentedura (2006) proposed that for true transformation of learning, implementation must aspire towards redefinition of pedagogy, rather than simply substituting one tool for another. However, in practical terms, the model is a spectrum of technology integration. As the potential application of technology in the classroom grows, the SAMR model is increasingly being used to influence good practice in classroom settings, and to support teaching professionals in their efforts to transform the pupil experience.

Researchers have also attempted to use the SAMR framework to categorise practice across schools, with varying success. Geer et al. (2017) found it difficult to distinguish between the four stages when assessing the extent of implementation, however resolved this by classifying technology use as either enhancing or transformative. They found most teachers using technology to enhance rather than transform their practice, potentially due to the relatively recent adoption of such technology. The SAMR tool has its limitations, not least that it focuses on the technology itself and ignores wider modifiers such as teacher and pupil knowledge and attitudes, and the wider dynamics within the classroom (Hamilton et al., 2016). The authors also note that there is limited information provided by Puentedura on the theoretical background or supporting evidence to the development of the model, nor is there peer‐reviewed literature. However, as a model by which to categorise the extent of implementation, it is a useful one, for both educators and researchers.

TPACK and SAMR are of course not the only implementation models, however are prominent in research and practice discussions. A more simplified version of SAMR – RAT (Replacement, Amplification, Transformation) developed by Hughes et al. (2013) – makes practical application clearer by generalising augmentation and modification, which have been criticised as being difficult to distinguish between. Puentedura also drew parallels with ‘Bloom's Taxonomy’ (Bloom, 1956), a long‐established pedagogical model of the learning journey, from remembering and understanding new knowledge; then applying and analysing the knowledge; through to creating or generating new knowledge.

Overall, it is clear that simply providing mobile devices to all primary school classrooms is not enough to improve child educational outcomes. The ways that these are used to motivate learners, the types of activities undertaken, the skills and knowledge of the teacher and the added value to existing practice, are key. While these implementation models have been criticised as being too simplistic (see e.g., Choy & Ng, 2015), given the dynamic natures of both pedagogy and technology, and the number of factors that may impact implementation in the classroom, they make a valuable contribution to our understanding of how technology may enhance educational outcomes, and the factors for consideration in practical implementation in the classroom. Importantly, both theories remain relevant despite the rapidly changing nature of technology and its application in the classroom. While the meta‐analysis in this review considers only primary outcomes, these implementation considerations are critical to the qualitative analysis, and the interpretation of findings.

### 2.4 Why it is important to do this review

There are existing meta‐analyses and reviews on a similar theme, but with limitations. A systematic review by Haßler et al. (2015) is most similar, however, is not a registered systematic review, which may have implications for the robustness of the review. The searches were undertaken in 2014, following which there has been rapid growth and evolution of the use of mobile technologies in the classroom. The current review therefore draws on more recent research. Furthermore, Haßler et al.'s review considered both primary and secondary school use, did not include smartphones, and focused on wider learning outcomes. There is no sub‐analysis completed, either across age groups or specific learning outcomes. Given the differences in curriculum content and teaching approaches in primary and post‐primary schools (or equivalent), a closer look at primary school practice is merited.

A protocol is currently registered with the Campbell Collaboration (Liabo et al., 2016) with a focus on the impact on academic achievement (including literacy, numeracy, and wider knowledge) and on school engagement (as measured by attendance patterns and school enjoyment) of schemes which primarily seek to increase pupils' wider access to technology, such as discounted laptop schemes or Internet access. These devices are not necessarily for use within the classroom, rather may be used at home or within the community. Additionally, the final review has not yet been completed.

Most recently, Dietrichson (2020) undertook a systematic review of school‐based interventions to improve reading and mathematics for students with or at risk of academic difficulties in grades Kindergarten to six (primary school equivalent). While there is some crossover, this review included all interventions rather than those specifically using mobile devices, and focused on targeted interventions for those experiencing educational delays, rather than on those for the class as a whole.

A full list of further reviews identified is included in Supporting Information: Appendix 1 along with details of their area of focus and limitations in relation to this review. In summary, existing reviews differ from the proposed review in a number of important ways. They tend to be focused on older or younger age groups of children (pre‐school, post‐primary, higher education) without sub‐analysis on the age‐group of interest, or focused specifically on pupils requiring additional support or with special educational needs, rather than general usage of mobile devices in the classroom. They are often inclusive of all technologies (including e.g., interactive white boards and desktop computers) rather than focused specifically on mobile devices, or more narrowly focused (e.g., on iPad branded tablets only). Given the speed of development of technology and the rapid evolution of technology applicability in the classroom, the searches are quickly outdated. Finally, they may not meet the standards set out by the Campbell Collaboration in terms of systematic review methodology, for example, by including peer reviewed journals only, or excluding grey or unpublished literature. For this reason, the current review usefully adds to existing knowledge.

Any innovation in the classroom has the capacity to impact all pupils and must be implemented by professionals who are equipped with the skills and knowledge to use it appropriately and effectively to support pupil attainment. This review will provide an accessible resource for policy makers, educational practitioners, and technology developers in the world of primary or elementary education. It has important policy and practice implications across several areas, including curriculum development and delivery; technical provision in schools; school policies and infrastructure, and teacher training and professional development.

## 3 OBJECTIVES

This systematic review sought to understand how mobile devices are used in primary/elementary education around the world. The study aimed to identify and synthesise high quality research (published and unpublished) to determine how activities undertaken using these mobile devices in the primary classroom might impact literacy and numeracy attainment for the pupils involved. Within this context, mobile devices are defined as tablets (including iPads and other branded devices), smartphones (usually those with a touchscreen interface and internet connectivity) and handheld games consoles (again usually with touchscreen and internet‐enabled). The interventions of interest are those aimed at improving literacy and/or numeracy for children aged 4–12 within the primary/elementary school (or equivalent) classroom.

Specifically, the review aimed to answer the following primary research question and five supplementary questions:

1. What is the effect of mobile device integration in the primary school classroom on children's literacy and numeracy attainment outcomes?
2. Are there specific devices which are more effective in supporting literacy and numeracy? (Tablets, smartphones, or handheld games consoles)
3. Are there specific classroom integration activities which moderate effectiveness in supporting literacy and numeracy?
4. Are there specific groups of children for whom mobile devices are more effective in supporting literacy and numeracy? (Across age group and gender).
5. Do the benefits of mobile devices for learning last for any time beyond the study?
6. What is the quality of available evidence on the use of mobile devices in primary/elementary education, and where is further research needed in this regard?

### 3.1 Stakeholder engagement

Chapter 2 of the Cochrane Handbook (Thomas et al., 2019) highlights the importance of stakeholder engagement throughout the review process, from defining priority topic and review questions through to interpreting review findings in relation to everyday practice. A participatory approach has therefore been incorporated to ensure stakeholder engagement throughout this review process. Cottrell et al. (2015) identified several benefits of stakeholder engagement in systematic reviews, including Increased credibility; the ability to anticipate controversy; transparency and accountability; improved relevance; enhanced quality; and increased opportunity for dissemination and uptake of findings. They also identified several challenges, including the time required to engage stakeholders; training and resources needed; and the process of engaging appropriate people at the appropriate time.

An Expert Advisory Group was established early in the process to shape the review focus and bring expert knowledge about everyday practice, as practical primary school teaching experience was not amongst the skills of the core systematic review team. The group comprised four members, including a primary school vice‐principal and a primary school senior teacher (both technology leads within their schools); a parent of three primary‐aged children; and an educational policy professional with expertise in the use of technology within the primary school curriculum. The advisory group was small to align with reviewer capacity, but brought key knowledge and skills in terms of practical application of technology in education – from the perspective of teacher, parent, and policy developer. To recruit advisors, emails were sent to pre‐existing contacts, and recommendations were followed up. A summary paper and Terms of Reference for the Expert Advisory Group was developed and shared with proposed members to ensure informed consent (See Supporting Information: Appendix 2).

The group met on three occasions during the review process (one face to face meeting and two online meetings, due to COVID‐19 pandemic restrictions), with further engagement via email between meetings. The first meeting took place in the early stages of the review process, before title registration. In addition to introducing the review, discussion focused on the types of technology used in classrooms, with the advisory group supporting a narrowed focus from technology more broadly to the specific use of mobile devices, in line with their experience of current practice. The proposed focus of literacy and numeracy was also discussed at the meeting and the group agreed this was a common area of interest for all primary school teachers and therefore of practical relevance.

Following the first meeting, group members were engaged in several email discussions on the common devices and applications used in primary schools to support literacy and numeracy learning, which helped to refine the interventions of interest and inclusion/exclusion criteria for use in the search process. A follow up online meeting then took place to discuss the feedback and propose the focus and approach to be taken in the review. This feedback contributed to protocol development and submission, and to the final methodology employed.

A further email activity took place following identification of the final set of included studies, to support the classification of included interventions under the SAMR framework classification. A summary paper (Supporting Information: Appendix 3) was shared with group members, describing the SAMR framework and stages, the interventions identified in the included studies, and the key features of each. Group members were invited to use their professional experience to classify each intervention as substitution, augmentation, modification or redefinition of ‘normal’ practice. Following email feedback, an online focus group was held with the Expert Advisors to discuss their conclusions and finalise classifications for each included intervention. At this stage, a wider discussion was also held with the group on how the research interventions compared to ‘real life’ practice, and where they felt the benefits of technology lay from their personal experience. This focus group was recorded and transcribed, and the reflections used to support interpretation of the findings. Finally, a draft Plain English Summary was shared with Advisors for review and comment to ensure accessibility to non‐researchers.

## 4 METHODS

### 4.1 Criteria for considering studies for this review

#### 4.1.1 Types of studies

This systematic review, and the method described below, is based on a pre‐published protocol (Dorris et al., 2021). The search criteria used to identify studies for inclusion focused on the participants studied, the intervention undertaken (including outcome/s of interest, delivery method and venue in which the intervention was delivered), and the primary research methodology adopted (types of studies).

Connolly et al. (2018) found the use of RCTs in educational research to have increased significantly, and their applicability to have been demonstrated. RCTs are considered amongst the highest quality standard of evidence, therefore, only studies which reported effect sizes through the comparison of intervention and control groups either through RCTs or Cluster RCTs were eligible for inclusion in this review. Control groups could include either traditional teaching methods which did not incorporate technology (no intervention), or an alternative technology (e.g., desktop computers). Included interventions must have been time‐equivalent – therefore, interventions that resulted in pupils receiving additional tuition beyond standard class time were excluded. Quasi‐experimental, non‐experimental, or qualitative studies were excluded. Qualitative data was extracted from the final selected studies to provide some background to the differing interventions, and support subgroup analysis, however wider analysis of the content/approach of interventions and their theories of change was not possible within the review scope.

#### 4.1.2 Types of participants

The included population for this review was children within mainstream primary, elementary or kindergarten education settings in any country (with ‘mainstream’ referring to the dominant statutory educational provision of the country). These children are usually in the age range 4 to 11, however on occasion some children aged 12 were included as it was not possible to isolate the effects across different age groups. There were no cases in which both primary and post‐primary aged pupils were included within a study. Studies which assessed the use of mobile devices in special schools, educational provision other than at school, informal preschool settings or indeed home schooling, were excluded. Additionally, interventions targeted at a sub‐group of low‐performing students, rather than the class as a whole, were excluded. Eligible studies from all countries were included if they were returned by the search, however, it is important to acknowledge that searches were conducted using the English language across databases which overrepresent English language and research.

#### 4.1.3 Types of interventions

Included in the review were interventions initiated within the primary school classroom (or global equivalent) that used mobile devices (including tablets, smartphones, or hand‐held gaming devices) to intentionally support learning for the class as a whole. Interventions were considered where delivery was by the classroom teacher, or a researcher, as long as it was delivered within the usual day to day class time. In all interventions, the device must have been used directly and primarily by the child, although some use by the teacher alongside this was acceptable. The decision on which devices to include was discussed with the Expert Advisory Group and informed by the earlier literature review. Tablets were considered the most likely device used in classrooms; however smartphones and handheld games were mentioned in literature and are cheaper and more accessible, therefore important to capture. Laptops, chromebooks and similar were excluded from the study as they lack the portability, dexterity and manipulation that tablets and smaller devices bring, therefore were felt to provide a different overall experience. Table 5 summarises these criteria.

**Table 5:** Inclusion and exclusion criteria for interventions.

Eligible interventions included	Ineligible interventions were those which
Interventions which used tablets, smartphones or hand‐held gaming devices. Interventions which focused on literacy or numeracy outcomes. Interventions using apps, websites accessed through a mobile device, or preloaded software. Interventions where students directly used the mobile device themselves, either individually or in pairs or in groups. Those targeted at the whole class, rather than delivered to a subgroup to address learning deficit. Both one‐off and regular activities (however dosage is considered when comparing studies at analysis stage).	Used technology other than mobile devices as specifically defined above. Had no specific focus on literacy or numeracy. Involved teacher usage of the device, but pupils had no direct engagement with the device. Took place outside of core curriculum delivery time, or which did not take place within the mainstream classroom. Were targeted at children with learning difficulties or delays in an effort to help them ‘catch up’ with peers.

#### 4.1.4 Types of outcome measures

While the literature on technology integration in primary school classrooms considers a wide range of influencing factors, including enhanced motivation and engagement with peers (see e.g., Ciampa, 2014), only academic performance outcome measures were included in this study. Studies which focused on improvement in any element of literacy or numeracy were considered for inclusion.

##### Primary outcomes

In planning for this review, a range of source material was read, and a list compiled of the types of outcome measures used or reflected in papers and studies. The primary school curricula from across the four UK Nations were also reviewed to identify the elements of literacy and numeracy taught. The wide range of potential literacy outcomes reflects the complexity of the subject and the multiple skills that effective literacy requires. These can be classified under three categories. Listening outcomes focus on hearing sounds, correctly combining sounds into words, and identifying the sound or word on the screen. Reading and writing outcomes include the identification of written words, accurately and fluently, and accurate spelling and grammar when writing. Thirdly, comprehension outcomes measure the understanding of what has been read and decision‐making ability based on information available. These skills are usually learned in order of complexity, therefore measures assessing comprehension were more likely to be used with older children. Similarly, several common numeracy elements are assessed in studies. These can be grouped in three categories. Mathematical knowledge includes number recognition, identification of operators (subtraction, addition, multiplication, division) and how to use them (incorporating accuracy and fluency). Mathematical thinking covers problem solving, reasoning, spatial awareness and working memory, while complex operations include geometrical concepts and number manipulation. Again, children progress through these skills therefore older children are more likely to be assessed in use of complex operations.

Within the scope of literacy and numeracy, many outcome measures are used, including standardised assessments, bespoke tools and statutory academic assessment of the country. The inclusion of specific outcome measures was not used as a criterion for study inclusion, however the measures used across a range of studies were used to identify search terms.

##### Secondary outcomes

No secondary outcomes were considered in this meta‐analysis.

### 4.2 Search methods for identification of studies

A sensitive and comprehensive search strategy was designed, including electronic and other sources. This is summarised below.

#### 4.2.1 Electronic searches

Electronic databases and other search sources were identified in advance and stated in the published protocol (Dorris et al., 2021), in line with best practice in systematic reviews (see Supporting Information: Appendix 4). In compiling the final list of databases, a combination of professional experience, library subject guides and pilot searches were used to identify the most relevant for inclusion. The final search strategy incorporated relevant journal and other databases with a particular focus on education and social sciences, however as recommended by the Campbell Collaboration (Kugley et al., 2016), both field‐specific and multidisciplinary databases were searched. The search strategy was designed to retrieve both published and unpublished literature, including government research or studies by non‐governmental organisations, conference papers and reports on proceedings, technical reports, dissertations and theses, white papers, and other relevant unpublished literature. Searches took place between October and November 2020, with databases accessed through Queen's University, Belfast, and via the Internet where relevant. As noted above, an update of the searches would be relevant in terms of identifying newly published studies to build on this work.

To conduct searches in the databases identified, first a broad groups of relevant search term groupings was developed, as described in Table 6. An initial list of search terms was then compiled within each grouping by reviewing keywords and subject headings from a sample of randomly selected, relevant articles, and subject terms used in ERIC and British Education Abstracts. Careful consideration was given to synonyms, country‐specific spelling, and alternative names for devices, for example, primary school/elementary school; mobile phone/cell phone; randomised/randomized. The final search terms and keywords are included in Supporting Information: Appendix 5.

**Table 6:** Search term groupings.

Search term grouping	Details
**1. Population of interest**	Combining broad terms for appropriate age with class/classroom/school.
**2. Setting**	Mainstream primary school setting, or global equivalents.
**3. Intervention of interest**	(a) Type of mobile device used (tablets, smartphones, handheld games consoles; all touchscreen and internet‐enabled) and (b) curricular topic addressed (i. literacy OR ii. numeracy and associated concepts).
**4. Study design:**	Randomised controlled trials only.

Using Boolean Operators, a sample search string was then developed (See Supporting Information: Appendix 6) by combining each grouping within ERIC as a trial database using the equation *1 AND 2 AND (3a OR 3b) AND 4*. This exhaustive process is what sets a systematic review apart from other forms of literature reviews, lending both robustness and transparency to the work.

As databases vary, the final search terms were adapted to suit each database by reviewing the database thesaurus, and again piloting the search string. Where available, database limiter functions were used for ‘school setting’ (education level), rather than inputting a search string. A record of each search completed was documented, including date of search, specific combination of keywords used, and total numbers of studies identified and retrieved (see Supporting Information: Appendix 7).

In undertaking searches in Google, a potential source of bias is introduced due to inbuilt algorithms which track user data to provide personalised search results. ‘Secure’ search engines available designed without tracking, such as DuckDuckGo, are available and can be used by systematic reviewers to avoid algorithm bias. However, various sources (Landerdahl, 2022; Rethlefsen et al., 2021) recommend that the use of ‘incognito mode’ within Google or Google Scholar will give the same results. Google Scholar was used in this review, with search history, location services and other personalisation options switched off to ensure this did not impact results by returning tailored search results. Google Scholar search function is limited to 256 characters (including operators) therefore a smaller, more targeted search string was developed, and the first 500 hits screened for relevance.

Grey literature and thesis/dissertation searching were important elements of this search strategy. Searches were undertaken via a range of relevant sources, including OpenGrey, Microsoft Academic Search, and ProQuest Dissertation and Theses. Additionally, government education websites were searched (across England, Scotland, Ireland, Northern Ireland and Wales) alongside websites of charities and funding organisations, including the Education Endowment Foundation, National Literacy Trust, National Numeracy Trust, and the British Educational Research Association.

In the pre‐published protocol to this study (Dorris et al., 2021), a date of 1990 onwards was proposed as a date limiter, given that devices such as the Delaware Fingerworks or Palm Pilot were in existence, however on closer consideration, these devices did not have comparable functionality to tablets and smartphones, or educational applicability as considered in this study. The decision was therefore made to focus only on ‘new technologies’. As iPads and similar tablets only emerged from 2010 onwards, this was considered an appropriate limiter.

#### 4.2.2 Searching other resources

Contact was made with authors prominent in the subject area via email, including the first and second authors of included studies and any others appearing regularly in excluded but relevant studies. Authors were asked to share details of any unpublished studies or work in progress, either of their own or known to them. Additionally, two relevant journals, the British Journal for Educational Technology, and Computers and Education, were identified through triangulation of information gleaned from identified studies, journal metrics and professional experience, and 5 years of editions were reviewed for relevant studies.

Alongside any conference proceedings identified through the grey literature searches above, several conference/s were identified as being of high relevance, including the International Society for Technology in Education; BETT; British Educational Research Conference and the European Conference on Education. These were selected given their global reach, relevance to primary/elementary education and technology, and focus on research and pedagogy rather than marketing opportunities for technological products. This decision was also informed by the Expert Advisory Group, several of whom had personal experience of attending EdTech conferences and were familiar with the focus of each. The conference proceedings from 2015 onwards were searched by hand to identify those not yet indexed in the commercial databases.

Reference lists of included studies were reviewed, relevant studies identified, and articles retrieved online (via QUB database). Bibliographies of other relevant systematic reviews or meta‐analyses were also reviewed, and relevant studies identified and retrieved. Finally, a citation index search of relevant databases identified any more recent studies citing the already identified studies.

### 4.3 Data collection and analysis

#### 4.3.1 Selection of studies

All searches were conducted by the first author, following the strategy set out above, while co‐authors supported the screening process. Eligible studies were imported to EPPI‐Reviewer screening software (Thomas et al., 2019), and duplicate records removed. Studies, rather than reports of studies, were the desired unit of analysis, therefore multiple reports of various aspects of the same study were manually linked via EPPI‐Reviewer to avoid double counting. All reports of the same study were used to glean available information.

**Title and abstract screening:** The first round of screening reviewed the title and abstract of studies for inclusion/exclusion. Screeners were given the following questions (and relevant responses):

1. Was the study undertaken from 2010 onwards? (If no, exclude on date)
2. Does the study consider the use of appropriate mobile devices in the classroom? (If no, exclude on intervention)
3. Are study participants for the most part in the correct age group (4–12) and within a primary school (or equivalent) class setting? (If no, exclude on population) (note a small number of pupils in a class may be outside of the desired age group, as specified in ‘types of participants’ above. Decision on inclusion was based on the age of the class majority).
4. Does the study focus on outcomes of interest? (Literacy or numeracy and related skills) (If no, exclude on intervention)
5. Do pupils use the device themselves (rather than the teacher)? (If no, exclude on user)
6. Is a control group design used? (If no, exclude on study design)

Where the answer was no to any one of the above questions, the study was eliminated; if the study was eliminated after any one question, no further questions were necessary. Where the answer was yes to all questions, the study was included for full text screening.

A test batch of 50 records was allocated to each reviewer for screening, and Cohen's kappa coefficient (*k*) calculated on the test batch to measure inter‐rater reliability. As per Cohen's original discussion, a *k* value of 0.41 or greater was considered ‘fair’ (Landis and Koch, 1977). This process was repeated, allocating further batches of 50 records for screening and *k* calculated until consistency was established across the team and the screening questions deemed appropriate. The first author then screened all remaining records, and distributed all records amongst co‐authors (K.W., L.O'H., E.T.L.) to ensure each record was independently screened by two reviewers. A record of Cohen's *k* is included in Supporting Information: Appendix 8. A third member of the reviewer pool was asked to provide additional assessment where *k* was considered low.

**Figure 3:**
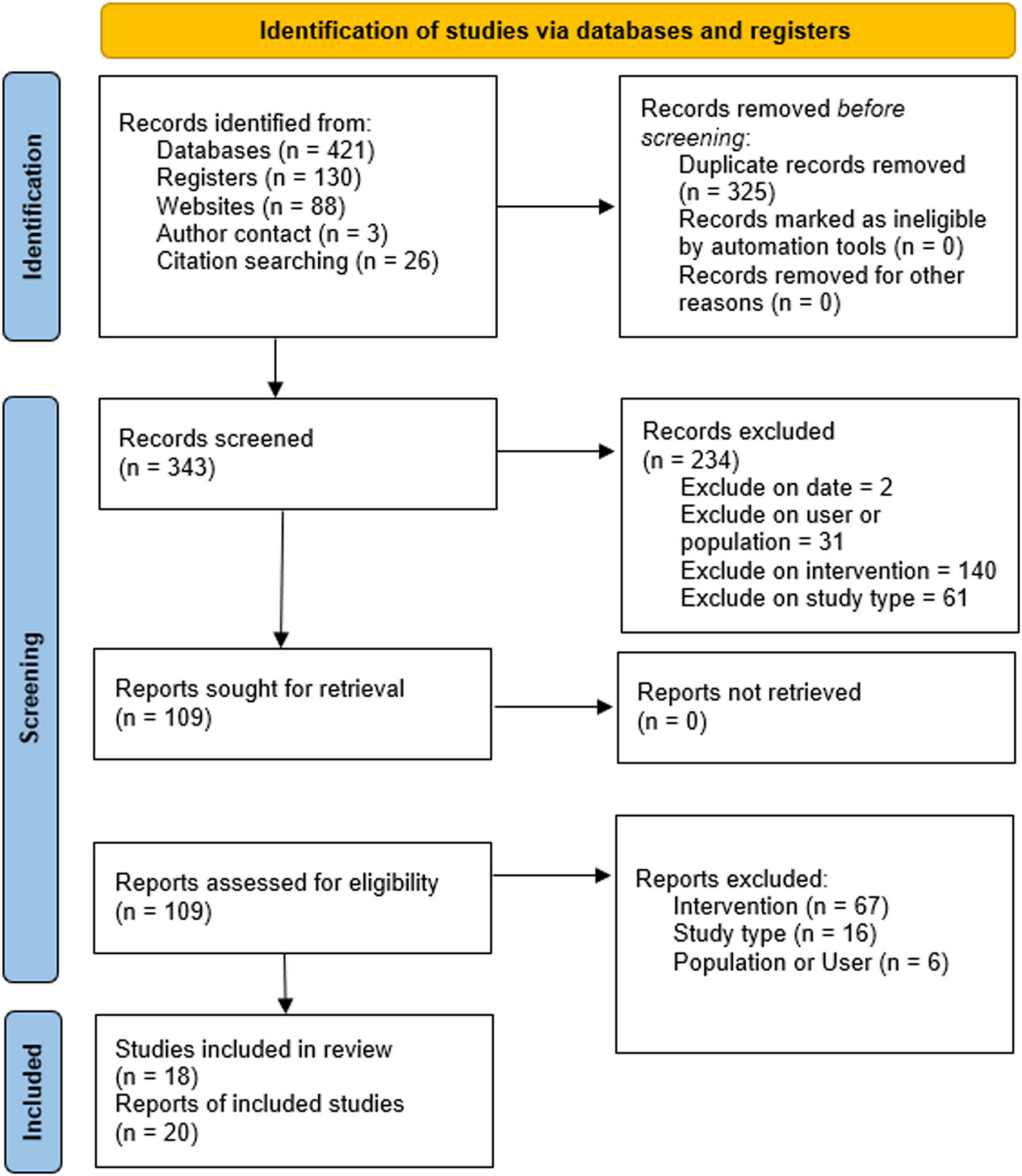
Flow diagram – Search process. *Source*: Page MJ, McKenzie JE, Bossuyt PM, Boutron I, Hoffmann TC, Mulrow CD, Mulrow CD, et al. The PRISMA 2020 statement: an updated guideline for reporting systematic reviews. BMJ 2021;372:n71. doi: 10.1136/bmj.n71. For more information, visit: http://www.prismastetement.org/.

**Full text screening:** Full text was retrieved for remaining studies deemed relevant, or where their relevance was unclear, and the dual screening process was again followed. As above, the first author screened all records, while co‐authors independently screened a selection each. For the most part, there was fair/moderate or significant agreement between screeners' decision. Any disagreements between reviewer decisions at each stage of the process were resolved through discussion with a third reviewer until consensus was reached. The screening process was fully documented using a PRISMA Flow Diagram (Figure 3) as specified in Chapter 4 of the Cochrane Handbook (Lefebvre et al., 2019). A list of ‘characteristics of excluded studies’ was compiled for those studies which met the search eligibility criteria but were excluded for a specific reason.

#### 4.3.2 Data extraction and management

A data extraction framework, guided by the Template for Intervention Description and Replication (TIDieR) (Hoffman et al., 2014), was developed and refined following identification and review of the final studies. Data extraction focused on key information about the study design and sample, intervention details and delivery approach, outcome measures and tools used, and overall impact. Only the primary author extracted general review information, while outcome data was independently extracted by two reviewers.

#### 4.3.3 Assessment of risk of bias in included studies

As the review included only randomised studies, the Cochrane ‘Risk of Bias’ (RoB2) tool was used to assess for bias (Sterne et al., 2019). Both the individual and cluster randomisation versions of the tool were used as appropriate. Studies were rated as having low risk, some concerns or high risk of bias across individual domains, and for the study as a whole. As before, two reviewers independently rated each study, with disagreements resolved in discussion with a third reviewer. Results of the Risk of Bias assessment are presented in the next section.

#### 4.3.4 Measures of treatment effect

Summary data was collected from each included study, and meta‐analysis undertaken. While the outcomes of interest (literacy and numeracy) were pre‐specified, the measures differed amongst studies. Summary outcome data was collected for each group (intervention and control) within each included study, including pre‐ and post‐test mean score and standard deviation for each outcome measure, and number of participants in each group. This reflected the final number of participants whose outcome data was analysed, rather than the number allocated at the start of intervention, therefore excluded those who did not complete the intervention.

Two reviewers independently extracted outcome data, with the first author extracting data from all studies, and co‐authors taking a selection each. Most data extraction and coding took place via EPPI‐Reviewer; however, outcome data was recorded in Excel to facilitate meta‐analysis in the chosen software of RStudio.

#### 4.3.5 Unit of analysis issues

Standard methods of combining effect sizes in meta‐analysis assume independence, and where this is not the case, may produce misleading results (Cheung, 2019). Dependence may arise where two or more intervention groups are compared with one control group, leading to double counting of the control group members. While this was the case in several included studies, both intervention groups did not meet the inclusion criteria, therefore one was excluded. Dependence also arises where multiple outcome measures are reported for the same participants (correlated effects), leading to non‐independent effect sizes. This was the case in many included studies. This can be addressed by including just one outcome measure in meta‐analysis for each set of participants, however this would have led to the inclusion of only 18 outcome measures from a potential 46. Given the already small set of included studies, this would minimise the study power. An alternative approach is the Robust Variance Estimation (RVE) method (Hedges et al., 2010), which can be used to deal with non‐independent effect sizes. In addition, the RVE analysis method incorporates small sample corrections which can reduce inflated type 1 errors due to clustering in cluster randomised trails (of which there were nine). Details of this model and the calculations involved are discussed further below. No studies reported multiple outcome measures at follow‐up periods beyond immediate post‐test, meaning no conclusions can be drawn about the potential long‐term benefits of the interventions (research question five).

#### 4.3.6 Dealing with missing data

Where the study report was missing key data, the reviewers attempted to calculate the required measures from reported data (e.g., calculating standard error from confidence intervals or *p*‐value). However, where this was not possible, the author was contacted to request data. Where this still did not yield required information, the data was excluded from the meta‐analysis.

#### 4.3.7 Assessment of heterogeneity

Education research tends towards a high level of between‐study variability (heterogeneity) due to the variety of pupil age groups and backgrounds; school types, settings and leadership structures; subjects studied, and interventions undertaken. Therefore, variability across the final set of included studies was assumed and a random effects model selected for statistical analysis. However, variability was also confirmed through statistical means. Cochran's *Q* calculates the proportion of variation in observed effects that is due to variation in true effects. As *Q* has low power when the number of studies is low (as is the case in this meta‐analysis), *I* ^2^ was also calculated and reported, with *I* ^2^ > 50% considered moderate heterogeneity and *I* ^2^ > 75% considered large heterogeneity.

#### 4.3.8 Assessment of reporting biases

**Publication and time‐lag bias:** The search strategy reported above was constructed to minimise risk of publication bias, including multiple publication, or non‐publication. A funnel‐plot was also constructed, plotting study precision against effect size, and inspected for symmetry. Additionally, as this assessment is largely subjective, Egger's regression test was conducted (Egger et al., 1997).

**Outcome reporting bias:** There may also be bias in terms of the specific outcomes reported on in a study, with data only partially reported, particularly if one or more outcome areas or subsets produce more significant findings. As above, the RoB2 tool was used to assess potential bias in this regard.

**Location and language bias:** language and location were not used to limit searches. The published protocol stated translations would be sought where studies were not presented in English, and where a translation was unavailable, the study would be included as ‘unclassified’ and potential bias assessed and discussed.

#### 4.3.9 Data synthesis

##### Descriptive analysis

Descriptive data on the 18 included studies was first presented in a series of tables and charts to provide a summary of the key characteristics of the studies included and identify patterns. Data presented includes year of publication, geographical location of the study; literacy versus numeracy; type of device used; intensity of intervention (low, medium, or high); intervention characteristics; participant demographics and educational setting. This information is particularly useful to assess patterns in the research undertaken and identify gaps for future research – one of the aims of the systematic review process.

##### Meta‐analysis

As there were 18 included studies, all of which included a Randomised Controlled Trial or Cluster‐Randomised Trial, meta‐analysis was applicable. This was undertaken using various packages in RStudio to determine the overall effectiveness of mobile devices in supporting literacy and/or numeracy development within the primary school classroom.

To synthesise the main effects across all identified studies, Standardised Mean Difference (Cohen's *d*) was calculated for each, as all dependent variables were continuous data. Cohen's *d* is primarily calculated from post‐test score, standard deviation and group size for the control and intervention groups, and this was the primary outcome data sought. However, as noted above, where this information was not reported, other reported data (such as standard error; *t*‐tests and *p*‐values; mean gain scores and gain score standard deviation; or mean and ANCOVA) were used to calculate Cohen's *d* where possible, using the online effect size calculate provided by David B. Wilson (Practical Meta‐Analysis Effect Size Calculator). Where appropriate outcome data could still not be retrieved, the lead author was contacted to request the required information (see Supporting Information: Appendix 9 for sample correspondence template); this was the case for four of the studies. Responses were received from all four, however appropriate data for meta‐analysis was not available for one of the eligible studies (Bebell & Pedulla, 2015), leading to the exclusion of several outcome measures for this study.

##### Qualitative analysis

As noted above, qualitative studies were not included. However, alongside meta‐analysis, some qualitative analysis of review information took place across the included studies to identify themes in terms of the approaches to research taken, applications and devices used, and the intervention activities. While the primary aim of the systematic review was to draw conclusions about the impact of mobile devices on academic achievement in maths and literacy, it is also important to ask the questions why, how and for whom this impact is achieved. Qualitative analysis provides the background knowledge through which to interpret the meta‐analysis findings, and support application to practice. An open coding approach was used, in line with Glaser and Strauss (1967) Grounded Theory.

#### 4.3.10 Subgroup analysis and investigation of heterogeneity

Moderating factors can tell us something about why an intervention may work, or for which groups it is more effective. Subgroup analysis allows various groups of effect sizes to be meta‐analysed in isolation to determine if one produces a larger overall effect than others. As above, calculations were performed in RStudio, and results are reported below. Within this review, five prespecified subgroup analyses were undertaken, reflecting both intervention and participant characteristics.

Firstly, subgroups were analysed by degree of enhancement to normal practice to identify if effectiveness was moderated by judgement of activities as substitution, augmentation, modification, or redefinition of standard teaching practice as per Puentedura (2006) SAMR Framework. As the review team did not include a practising primary school teacher, and therefore was unfamiliar with ‘normal’ practice in the classroom, members of the Expert Advisory Group independently rated interventions via the SAMR Framework. A focus group was facilitated to discuss responses and agree a final SAMR rating for each intervention, summarised in the ‘characteristics of included studies’ table (Supporting Information: Appendix 10). Screen size was also considered, to determine if small screens (less than seven inches) were more effective than larger ones. In this instance, screen‐size is used as a proxy for type of device – with smaller screen sizes usually relating to handheld games or mobile phones. This will have important implications for future practice, particularly since the number of children with their own smartphone (with typically smaller screens than tablets) is increasing (OFCOM, 2019), and as schools begin to consider implementation of Bring Your Own Device policies to make use of children's own devices in school for educational purposes. Finally, in terms of intervention characteristics, the potential impact of dosage of delivery (reported in hours for most studies) was assessed. Country of study was not included in moderator analysis given the low number of studies for each country and the small number of countries included, however in the future when more research is available, differences by country will be of interest.

Moderating effects of participant characteristics were also considered, including gender and age. As discussed in the literature review, boys were considered more enthusiastic technology users than girls (Bergin et al., 1993), however more recent research (see e.g., Mullan, 2018) concludes that girls and boys are now equally proficient but with a preference for different activities. Any potential difference in impact across the genders in terms of educational outcomes will have implications for practice and is therefore an important moderating factor. Similarly, studies included a broad age range of children, from aged 4 and in kindergarten class, up to 12 in final stage of primary or elementary education. It was important to determine if effect size differed by age group, therefore an additional moderator variable (not specified in the protocol) was included. Within RStudio, dummy codes were set up to incorporate these moderator variables.

#### 4.3.11 Sensitivity analysis

Although effort was made throughout the review process to remain objective, there were various stages at which decisions made may have impacted final conclusions. Sensitivity analysis allows for studies, or parts of studies, to be systematically excluded from the meta‐analysis calculations, and findings compared to assess whether the overall effect size remains robust despite decisions taken or is skewed by the inclusion/exclusion of particular studies. In the final sample of included studies, the only potential concern lay in those studies with a high or unclear risk of bias. Therefore, the meta‐analysis was run twice with and without those studies rated as high risk.

## 5 RESULTS

### 5.1 Description of studies

#### 5.1.1 Results of the search

The searches were undertaken between October and November 2020, using the process pre‐specified in the Protocol (Dorris et al., 2021). Searches returned a total of 668 records as summarised in Table 7. A full breakdown of sources is included Supporting Information: Appendix 4. All records were imported into EPPI‐Reviewer Web software for screening. 325 records were identified as duplicates and excluded, leaving 343 results to be screened by title and abstract. Of these, 234 were excluded. 109 records were screened at full text, and of these, 89 were excluded, leaving 20 records. Reasons for exclusion at both stages of screening are summarised in Table 8. The most common reason for exclusion was due to interventions not meeting the requirements of the review – for example, carried out on desktop computers rather than mobile devices (e.g., Worth et al., 2018 evaluation of GraphoGame Rime), or where the mobile device intervention was a small part of a wider literacy or maths intervention (e.g., Cheung & Guo, 2018 and others' evaluations of ABRACADABRA literacy programme). Examples of the studies excluded are listed in the supporting documentation of this review.

**Table 7:** Summary of search findings by source.

Source searched	Relevant studies returned
Key database searches	421
Grey literature and relevant websites	88
Citation and reference tracking	26
Hand searching (journals and other sources)	130
Author contact	3
**Total**	**668**

**Table 8:** Reasons for exclusion at screening (title and abstract, and full text).

Title and abstract screening (343 records) Reasons for exclusion	Number of studies excluded
Exclude on date	2
Exclude on intervention	140
Exclude on study type	61
Exclude on population or user	31

Full text screening (109 records) Reason for exclusion	Number of studies excluded
Exclude on intervention	67
Exclude on study type	16
Exclude on population or user	6

Two records provided additional reporting of included studies, therefore a total of 18 unique studies met the criteria set out in the protocol for inclusion in the review and were taken forward for data extraction and analysis. Outcome data was available across all studies to enable inclusion in both qualitative and meta‐analysis. In line with conclusions drawn across systematic reviews in similar areas of interest (see e.g., Crompton & Burke, 2020; Tingir et al., 2017), this review confirms that robust research on the use of mobile devices in primary schools is emerging but will take time to build. This is as expected, given the relatively new nature of the technology, and the overall requirements and indeed relevance of Randomised Controlled Trials to these interventions. The full search process is documented in the PRISMA diagram Figure 3.

#### 5.1.2 Included studies

It is useful to examine the studies themselves in more detail to identify commonalities and differences, for example, in the types of interventions studied, or the populations within which the research was undertaken. A summary of characteristics of included studies is included in Supporting Information: Appendix 10. Below is a brief presentation and discussion on key characteristics.

##### Year of publication

**Figure 4:**
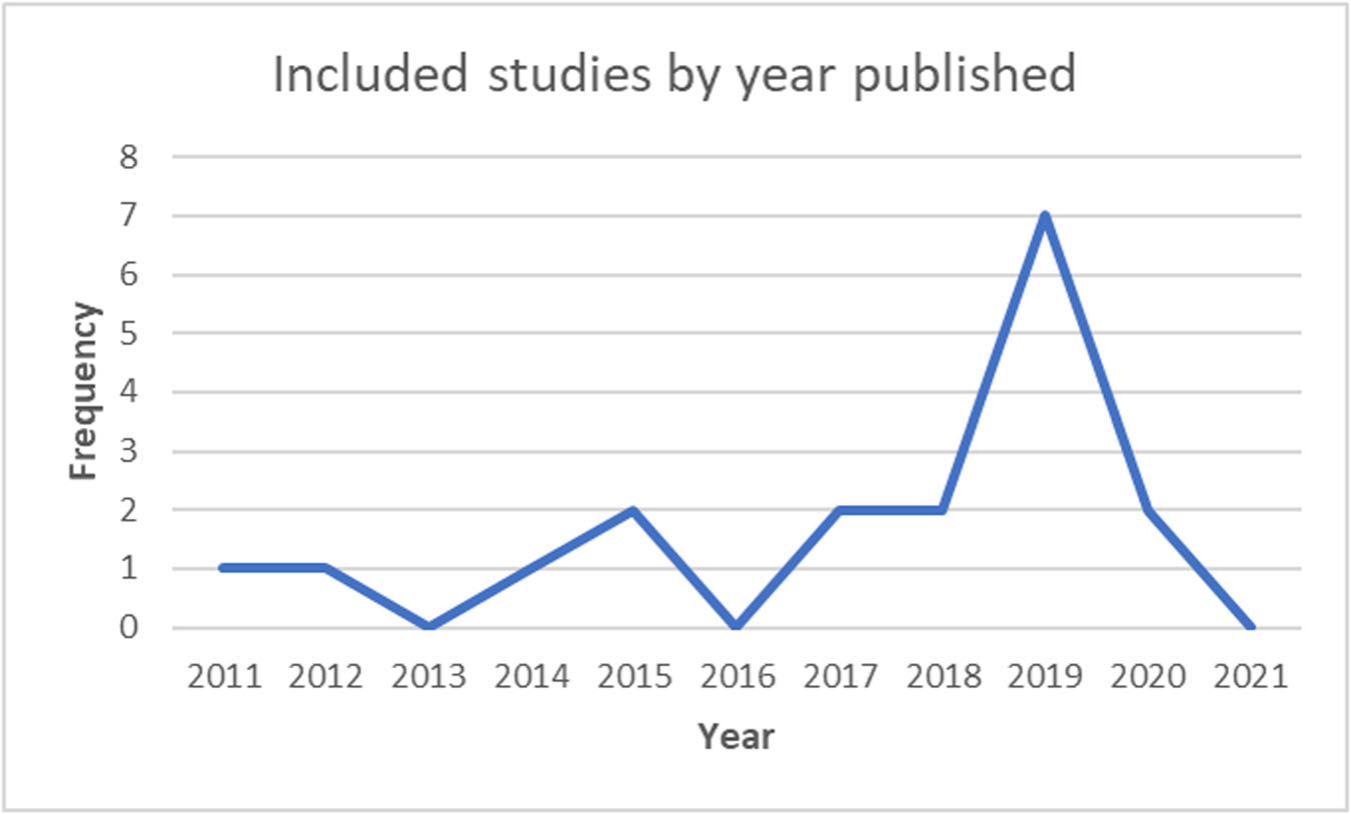
Studies by year of publication.

While the search criteria encompassed studies from 2010 onwards, all included studies have been published since 2011, and the majority have been published in the last 5 years (Figure 4). This demonstrates a small but growing interest in this area of education, and the need for further research. There are also several studies by the same research team which may account for this spike in studies. An update to this systematic review in future years will be important to clarify if there has indeed been a rise in research interest.

##### Geographical location of studies

**Figure 5:**
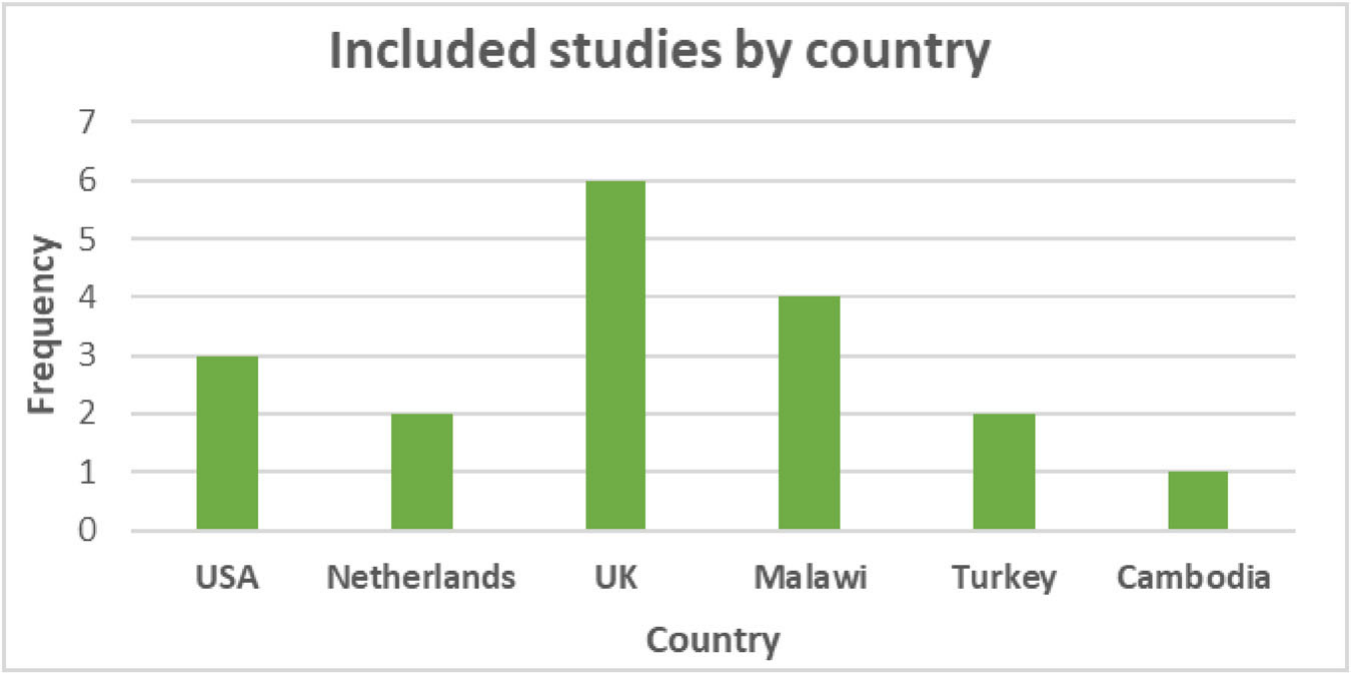
Studies by country of publication.

Of the 18 included studies, 3 were conducted in the USA, 2 in the Netherlands, 6 in the UK, 4 in Malawi, 1 in Cambodia, and 2 in Turkey (Figure 5). However, three of the four studies undertaken in Malawi had team members in common, and similarly the Netherlands studies had common teams. Since the number of studies is small, this does not reflect an increased interest in specific locations, rather reflects areas of interest for particular research teams and the interventions being studied. This may also reflect access to funding in particular regions.

##### Intervention setting

As one of the inclusion criteria for selection was that interventions must take place in a primary school class (or equivalent), there was minimal variety in the types of settings. Two studies took place in kindergarten or infant schools, however children were in the desired age range, while the remaining 16 studies took place in primary or elementary schools. There was insufficient information presented consistently across all studies to compare demographic differences meaningfully.

##### Sample size and participant characteristics

Sample sizes varied widely across the included studies. There were 11,126 participants across all included studies, with a mean sample size of 618.1 (SD = 669.73) ranging from 17 to 2133. The pupils ranged in age from 4 to 12. As studies did not provide a full breakdown of ages of individual pupils, it is not possible to provide an accurate distribution, therefore a crude summary has been presented to facilitate some moderator analysis incorporating participant's age group (Table 9). The most common age bracket was between 7 and 9 years old, aligning with year three and four in the UK primary school system. Similarly, a gender balance was not provided for all studies, however a crude classification has been given below (Table 10). This reflects that for 10 of the 18 included studies, there was an equal balance between boys and girls in the study (within 2% either way). Two studies had a higher percentage of girls, while two had a higher percentage of boys. Breakdown was not reported at all in the remaining four studies.

**Table 9:** Age of children in study samples.

Age of children in study	No. of studies	% of total
Age 4–6	4	22%
Age 7–9	10	56%
Age 10–12	4	22%
**Total**	**18**	**100%**

**Table 10:** Gender balance of children in study samples.

Gender balance of children in study	No. of studies	% of total
Equal numbers of boys and girls	10	56%
Higher % boys	2	11%
Higher % girls	2	11%
Breakdown not reported	4	22%
Total	18	100%

##### Intervention characteristics

There were 14 unique interventions assessed across the included studies. An exercise was undertaken to assess key characteristics of interventions aligned to the elements identified in the literature as being key to effective interventions (summarised in Supporting Information: Appendix 11). Commonly, interventions aim to make learning fun, using games or incorporating ‘real life’ activities (*n* = 13). Interventions often encourage autonomy, allowing the child to work at their own pace, reviewing items they are unclear of, or advancing to more difficult activities (*n* = 14), while some also adapt to match individual abilities (*n* = 10). Many interventions take a repetitive or rote approach to learning (*n* = 10), while others provide instruction, such as watching informative videos, reading notes, or retrieving definitions from an online dictionary (*n* = 6). Some interventions provide formative feedback on activities and performance, allowing the child to see their progress in real time and learn from errors (*n* = 12), while others also allow the teacher to monitor progress, and intervene where required (*n* = 8). Finally, only a few interventions promote creativity and combine complex skills (*n* = 3), or encourage collaboration between pupils (*n* = 2).

##### Moderating variables

The published protocol for this review identified several variables which may moderate the effectiveness of the intervention. Further detail on these variables, plus statistical analyses, are reported further below.

##### Intervention focus

Both literacy and numeracy interventions were included in this systematic review. Five studies focused on literacy, 11 focused on numeracy, and 2 included both literacy and numeracy outcomes.

##### Devices used and size of screen

In 16 of the 18 studies, children used tablets of 7 inches or larger to conduct intervention tasks. In the remaining two studies, one study used small mobile phones while the other used Nintendo DS Lite handheld games consoles; these both had screens smaller than seven inches.

##### Degree of implementation

**Figure 6:**
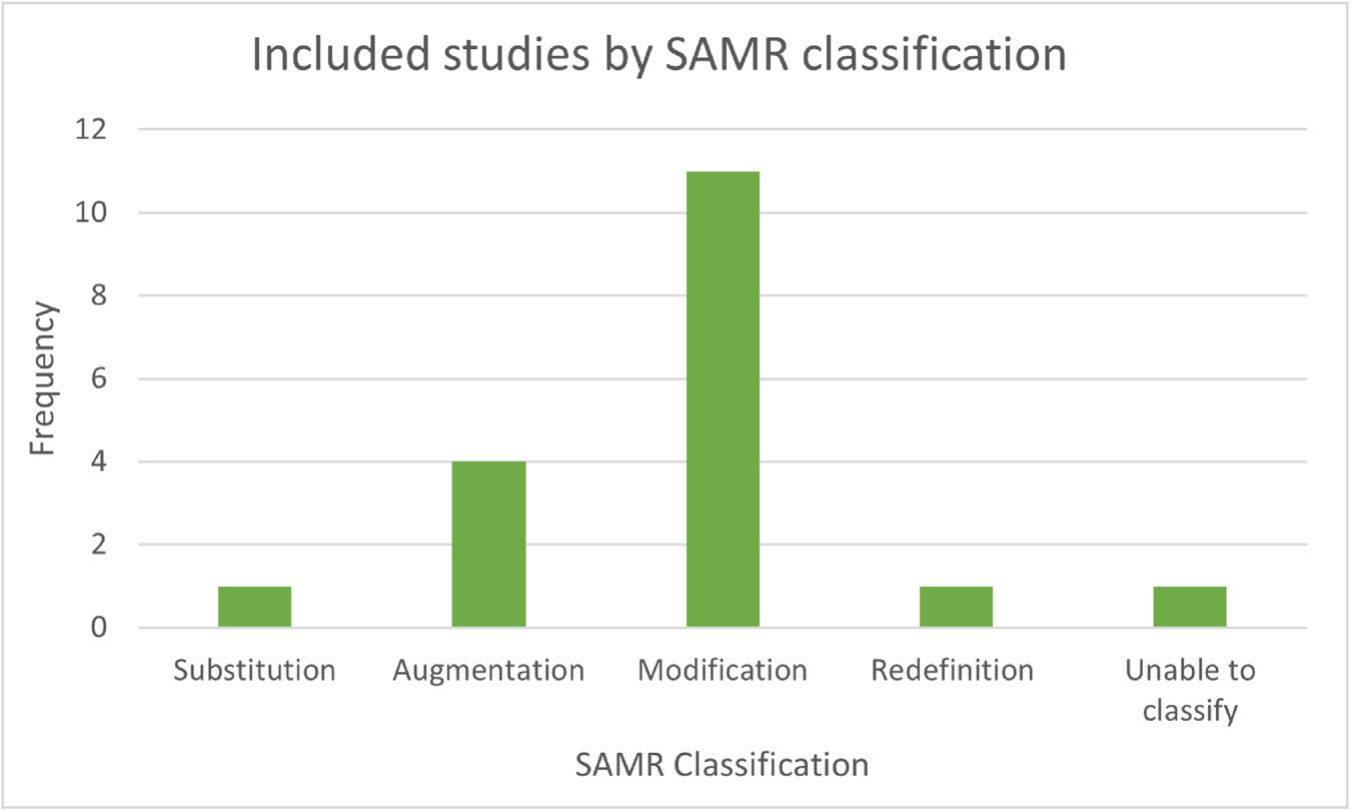
Studies by SAMR classification.

The degree to which the mobile device intervention enhances ‘normal’ class activity, rather than just substituting for a similar activity, was classified using the SAMR Framework (Puentedura, 2006). Ratings by the Expert Advisory Group are summarised in Figure 6.

By far the most common classification was modification – this reflects that the use of mobile devices allowed significant improvements to be made to ‘normal’ class activity. Only one study (Dundar & Akcayir, 2012) involved an intervention that simply substituted a digital activity for the same non‐digital activity (in this case, substituting a paper book for an e‐book, with no added functionality). Three studies were classed as including interventions that augmented ‘normal’ practice, that is, they replicated the normal activity, and added a little extra functionality. One such example was undertaking simple puzzles on the device that could have been undertaken using paper and pencil; additional functionality in this instance allowed mistakes to be deleted or second attempts made easily. Meanwhile, only one study (Yamaç et al., 2020) included an intervention considered to completely redefine the type of activity possible in the classroom. This ‘digital creative writing environment’ supported collaboration, creativity, multi‐modal activities, and feedback processes which would not have been possible in traditional ‘pen and paper’ creative writing approaches.

##### Intervention duration (dosage)

**Figure 7:**
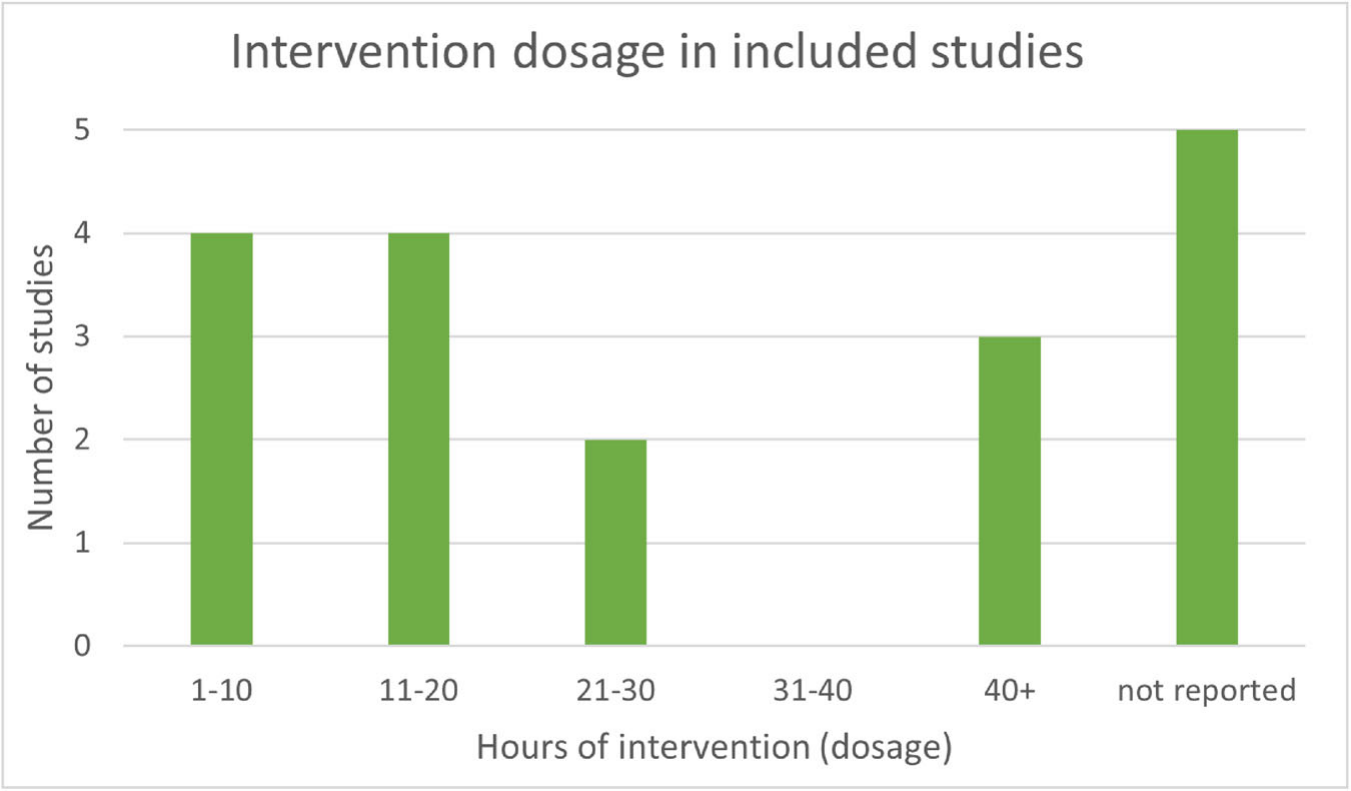
Interventions by dosage.

There were evident differences in the intensity of intervention received by pupils in each study. Where available, the duration of intervention in each study was recorded in hours and summarised for analysis purposes within groupings (1–10 h; 11–20 h; 21–30 h; 31–40 h; 41+ h). Distribution across these categories is summarised in Figure 7. Five studies did not report how often or for what duration children took part in the intervention. The minimum dosage was just 2 h in total (Messer et al., 2018) and the maximum dosage was 120 h (Levesque et al., 2020). This difference in dosage and intervention intensity likely has important implications for intervention effectiveness.

###### Analysis of outcome measures used across the studies

While all studies measured either literacy or numeracy outcomes (or both), within this, a wide range of specific skills and knowledge were assessed. While four of the included studies reported only one outcome for participants, the remainder reported multiple measures for each participant. A total of 46 relevant, unique outcome measures were identified across the 18 studies. Of these, 24 were numeracy outcomes, and 22 were literacy outcomes.

All outcome measures are summarised in Supporting Information: Appendix 12.

##### Data collection tools and approach

There were 19 outcome measures (10 studies) assessed using bespoke tools designed for the studies, while 27 outcome measures (8 studies) were collected using standardised measures (including statutory educational assessments). Standardised measures have several advantages. Firstly, they are accepted to be valid and reliable measures of the skills in question due to rigorous testing, with supporting evidence readily available. Secondly, many standardised measures are administered on a regular basis in schools for the population of interest, and where scores are disaggregated to pupil level, can support research in this area. It is useful to note that standardised tools also have disadvantages, for example, they can be expensive and require training to administer, and may not be as tailored to the intervention outcomes of interest as a bespoke tool may be.

Where bespoke tools were designed specifically for the research, only one study (Chen, 2014) provided no discussion on reliability or validity of the tool. Three studies (Messer et al., 2018; Schacter & Jo, 2017; and Yamaç et al., 2020) reported Cronbach's Alpha scores for the new tools (a common measure of internal consistency or reliability) with scores showing acceptable, good, or excellent internal consistency. Pitchford and Outhwaite (2016), published a separate study evaluating the reliability and validity of their bespoke tool administered via touchscreen tablet (with results demonstrating ‘proof of concept’ of a valid and reliable measure). This assessment tool was used as the outcome measure in Pitchford and Outwaite (2019) and Pitchford (2015). A further two studies (Fabian & Topping, 2019, and Miller & Robertson, 2011) cross‐checked their test items with experienced teachers in an effort to establish the validity of their tools.

The method of data collection was not specified for all measures. Since all interventions assessed use of mobile devices, some outcome measures were collected via the device itself, either completed by the pupil themselves or by an assessor. Other measures were assessed using paper/pencil tests, or orally (reading aloud or answering questions). One study (Pitchford, 2015) reflected on how administration via the mobile device may have given an advantage to intervention groups, due to their familiarity with the device leading to ‘practice effects’. This potential advantage may also be the case in other studies where similar collection means were used.

##### Literacy outcome measures

As noted above, literacy outcomes fall under a number of domains, including listening, reading and writing, and comprehension. Across the included studies, by far the most tested skills were reading and writing (14 measures, accounting for 63.6% of total literacy measures), as summarised in Table 11.

**Table 11:** Breakdown of literacy outcome measures.

Literacy domain	No. of outcome measures	% Total literacy outcome measures
Listening	3	13.6%
Reading and writing	14	63.6%
Comprehension	2	9.1%
Composite measures (including two or more of the above domains)	3	13.6%
**Total**	**22**	**100%**

##### Numeracy outcome measures

Within included studies, general maths knowledge, including number recognition and accurate and fluent use of operators tended to be most tested (13 outcome measures) while five outcome measures assessed reasoning and mathematical thinking, and only one measure considered more complex operations, in this case geometry. Again, more complex concepts are assessed in older children. Numeracy outcome measures are summarised in Table 12.

**Table 12:** Breakdown of numeracy outcome measures.

Numeracy domain	No. of outcome measures	% Total numeracy outcome measures
Mathematical knowledge	13	54.2%
Mathematical thinking	5	20.8%
Complex operations	1	4.2%
Composite measures (including two or more of the above domains)	5	20.8%
**Total**	**24**	**100%**

#### 5.1.3 Excluded studies

There were a variety of reasons for exclusion of studies at both stages of screening, with the most common being that the intervention was inappropriate to the aims of the review (*n* = 207). In many cases, the intervention did not use mobile devices, or used them as a small part of a larger intervention (e.g., the Bug Club reading programme, which combines online and print‐reading activities). In other retrieved studies, literacy and numeracy were not the focus of the intervention or were a small part of a wider subject. In other cases, the interventions took place outside the school classroom (e.g., in after‐school clubs) or in a childcare centre or for home learning. The second most common reason for exclusion was the study design (*n* = 77). For some, the study used was not an RCT, while in others, the study was an RCT however had no comparison with a non‐mobile device or ‘business as usual’. For a small number, the study involved secondary analysis of existing research, rather than being itself primary research (e.g., a systematic review). The third most common reason for exclusion was that the device user was not within the target population (*n* = 45). Children were either too old or too young; teachers or parents used the mobile device rather than the children themselves; or the intervention was targeted at pupils with special educational needs or low‐achieving subgroups, rather than mainstream class population.

### 5.2 Risk of bias in included studies

**Figure 8:**
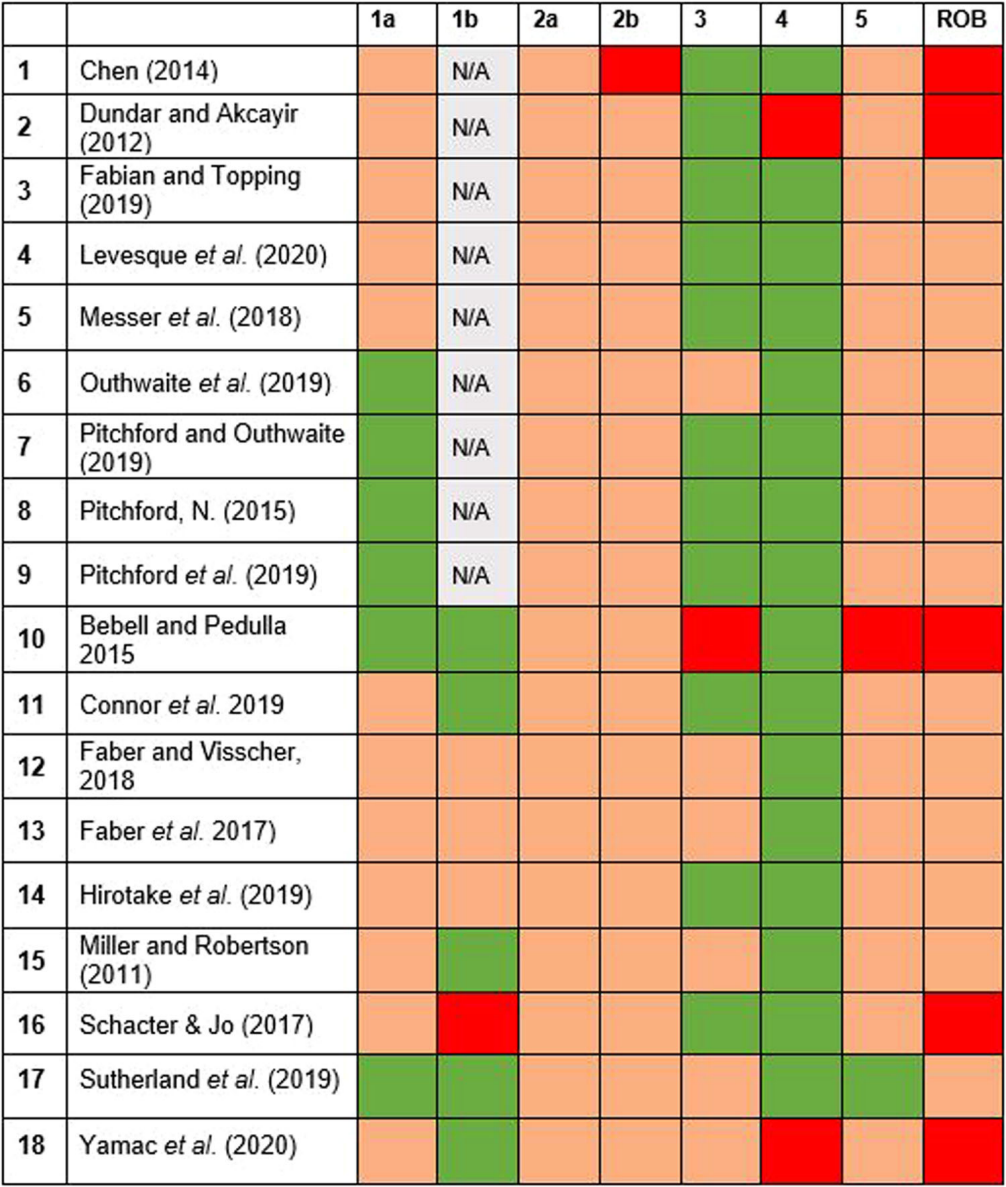
Risk of bias results.

All studies were assessed using the Cochrane Risk of Bias tool (ROB2) (Higgins et al., 2019) and variant tool for cluster randomisation (ROB CRT). Risk of Bias domains are summarised in Table 13, and each domain was rated as low risk, some concerns or high risk of bias. Each study was rated by the first author, and independently rated by one of the co‐authors. Ratings were combined and reconciled, and final assessment of bias for each study is displayed in Figure 8 (red, amber and green blocks represent high risk, some concerns, and low risk respectively).

**Table 13:** Risk of Bias domains assessed.

	Risk of bias due to
**1a**	The randomisation process
**1b**	The timing of identification or recruitment (cluster randomised trial only)
**2a**	Deviations from the intended interventions (effect of assignment to intervention)
**2b**	Deviations from the intended interventions (effect of adhering to the intervention)
**3**	Missing outcome data
**4**	Measurement of the outcome
**5**	Selection of the reported result

**Domain 1a – Risk of bias arising from the randomisation process:** Some risk of bias concerns arose for 12 of 18 studies, in the main due to the poor reporting of the randomisation process. While all studies included random allocation (individual or cluster), only six studies reported detail on the randomisation procedure (such as a computer programme or random number generator) or on the efforts taken to ensure the allocation sequence was concealed until participants were allocated to groups. However, most studies reported testing for baseline equivalence, therefore the lack of detail provided on randomisation procedures was considered as raising some concerns rather than high risk.

**Domain 1b – Risk of bias arising from the timing of identification or recruitment (cluster randomisation only):** Of the nine studies using cluster‐randomisation, one was rated as high risk, three as raising some concerns and five as low risk. The study by Schacter and Jo (2017) was considered high risk as information and parental consent forms were distributed only after classes had been randomised. It was unclear if parents were advised at that stage which group their child had been allocated to, which may have impacted their decision to consent to their child's participation. For those rated as having some concerns, reviewers felt there should have been more detail reported The process of ensuring informed consent within educational research, while not biassing the research itself, is an ongoing discussion (e.g., Gallagher et al., 2010), and research would benefit from a more careful reporting of the issues faced in practical terms and how these were addressed or resolved.

**Domain 2a and 2b** – **Risk of bias due to deviations from the intended interventions (effect of assignment to intervention, and effect of adhering to the intervention):** Blind allocation to intervention or control group is difficult in educational interventions as deviations from normal class activity are clear. However, for all studies, it was unclear whether the pupils were aware they were taking part in research, as there was no mention of pupil consent having been gathered. Only one study (Miller & Robertson, 2011) reported potential deviations from the intervention, with a reflection on possible ‘John Henry’ effects (Saretsky, 1972), noting that as the control group teachers knew they were being compared to the intervention group, they may have adjusted their ‘normal’ practice to compare more favourably. If this was the case, the difference between groups may be smaller than expected. There may have been similar, but unreported, effects in other studies too. Chen (2014) discussed implementation issues with the intervention, including insufficient devices to support all pupils to take part in class as planned, and dosage varied across the group; for this reason it was rated as high risk of bias. All other studies were rated as having some concerns due to the lack of detail provided to make an informed judgement.

**Domain 3 – Risk of bias due to missing outcome data:** One study was considered high risk (Bebell & Pedulla, 2015) due to poor reporting of outcome measures, lack of detail on final sample size and number of pupils included in the analysis. A further six studies were rated as having some concerns, largely due to the high attrition rates across the studies (up to 30% in one study). However, each study reflected on the reasons for attrition rates, how this may have impacted their findings, and how this was accounted for in analysis. In the case of studies conducted in Malawi (e.g., Pitchford et al., 2019), the high attrition rate reflected the general pattern of school attendance in the country, and this was discussed in the paper.

**Domain 4 – Risk of bias in measurement of the outcome:** Two studies (Dundar & Akcayir, 2012 and Yamaç et al., 2020) were rated as high risk in this domain. This was due to a complicated and subjective marking scheme, combined with no reported detail to judge if the assessors were blind to treatment status. All other studies were rated as low risk. There was strong reporting of the assessment procedures, with the same process being used for each pupil, and tests having right or wrong answers that did not require interpretation by the assessor. In four studies, pupils undertook the assessment themselves via the mobile device, with scores automatically generated, therefore were classed as outcome assessors themselves. A further six studies had assessments undertaken by trained assessors or researchers who were blind to treatment allocation. In five studies, teachers or researchers undertook assessment, but it was unclear if they were blind to treatment status, and the remaining three studies did not report details on who undertook the assessment. As already noted above, blind allocation in educational research is difficult due to the setting and the (often) involvement of the teacher. However, unblinded group allocation can lead to overestimation of effect sizes, as demonstrated, for example, by Ainsworth (2015) in a case study comparing blind and unblinded outcome measures in an educational study. Clear reporting and reflection on such issues is therefore critical.

Pitchford (2015) and Pitchford et al. (2019) reflect on the potential for assessment via iPad to have benefitted the intervention groups due to their familiarity with these following the intervention. While not specifically discussed in the other studies, this is a potential source of bias for all assessments conducted via iPad. However, Pitchford et al. (2019) undertook a comparison of paper‐based versus iPad administered assessment, and found similar response patterns, therefore this was not considered a concern.

**Domain 5 – Risk of bias in the selection of the reported result:** Within this domain, 16 studies were rated as having some concerns, one was rated as low risk, and one rated as high risk. In all but one study (Sutherland, 2019 – rated low risk) there was no pre‐specified plan reported for which outcome data would be collected and how it would be analysed. According to the ROB2 guidance tool, this automatically brings some concerns for potential selective reporting and analysis. Despite this, there was no evidence across 17 of the 18 studies to suggest that all eligible outcome measures were not reported on. Of more concern was the one study rated as high risk (Bebell & Pedulla, 2015) where only one outcome measured was fully reported and analysed (with a positive, significant result), out of a possible seven measures collected, raising concerns of reporting bias.

**Overall Risk of Bias:** Overall, 13 studies were rated as having some concerns, and five as having high risk of bias. No studies were rated as having low risk of bias. Poor reporting was judged across most studies, with many critical pieces of information missing which would have enabled a more accurate judgement on the research robustness. Several authors openly reflected on potential shortcomings in their own study, leading to assessment as having a higher risk of bias, while others may have experienced similar issues but not reflected on them.

Hartling et al. (2009) reported low inter‐rater reliability on a previous version of the ROB2 tool. Jørgensen et al. (2016) note that this implementation concern is commonly reported and suggest potential improvements including more detailed guidance and clarity on language used.

In many of the decisions made above, the level of risk of bias identified demonstrates a need for much more robust reporting of educational research, rather than confirmed shortcomings in the methodologies used. Simms et al. (2019) drew a similar conclusion following their systematic review of mathematics interventions. The ROB2 tool used above has already undergone several iterations, after the developers have sought user feedback to continue to make the process easier (Savović et al., 2014) and Cochrane is committed to further improvement work.

### 5.3 Effects of interventions

All 18 studies were included in quantitative analysis; however six outcome measures had to be excluded from one study (Bebell & Pedulla, 2015) due to missing data, despite contact with the authors to obtain this. Additionally, two studies included a measure of ‘time taken’ to complete a reading task – these were removed from meta‐analysis as they were out of character with the remaining measures and therefore not comparable within the analysis. In total, 40 outcome measures were included in meta‐analysis. All calculations were conducted on RStudio; the full R‐code script used in analysis is included in Supporting Information: Appendix 13.

#### 5.3.1 Inspecting for outliers

**Figure 9:**
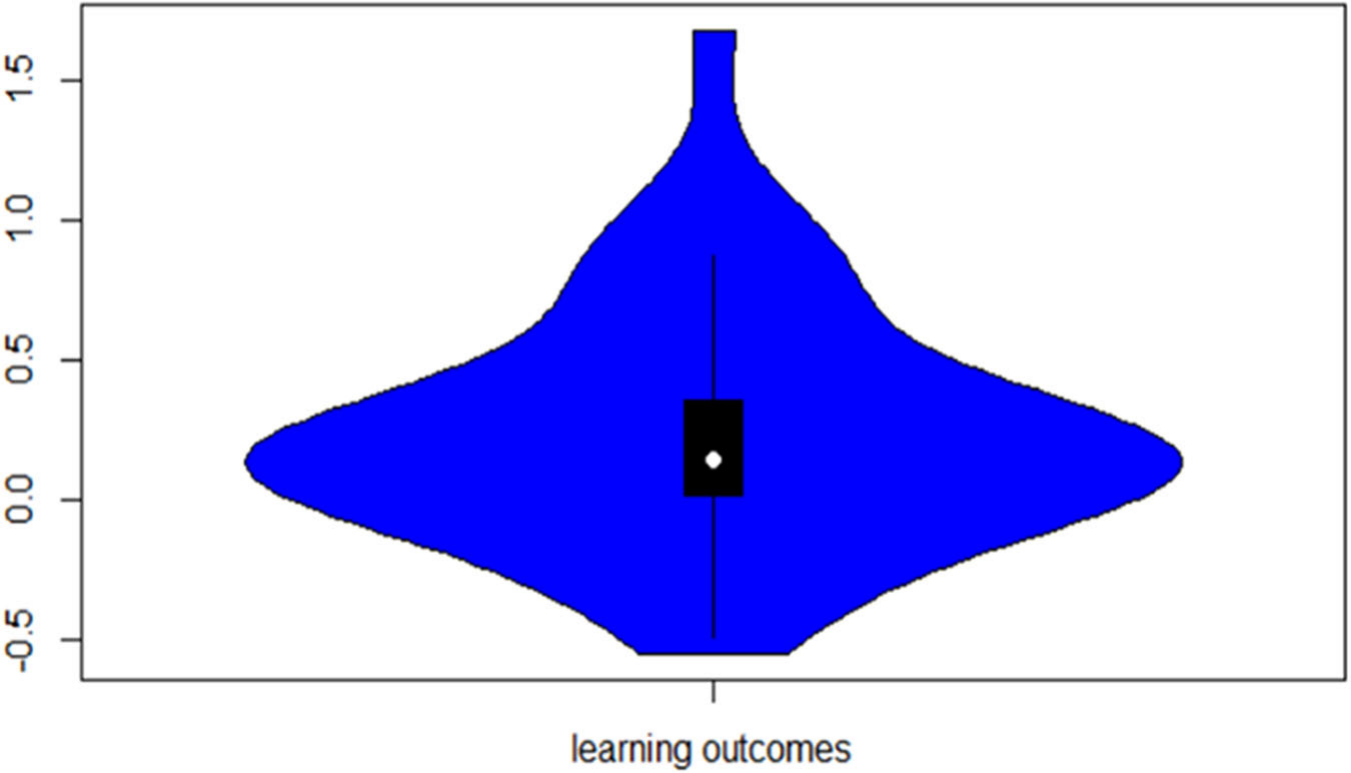
Violin plot for publication bias.

Before analysis, a violin plot was constructed using the vioplot package (Adler & Kelly, 2021) to identify effect size outliers. This combines a box plot with a kernel density plot and displays measures of central tendency: the median effect size (white dot), the interquartile range (thin black box) and the upper and lower adjacent values (thin black line). Any points lying outside these thin black lines are considered outliers. Figure 9 shows that the data is right skewed and approximately normally distributed, and while there appear to be some outliers, these are evenly distributed on both sides.

#### 5.3.2 Overall estimate of effects: Meta‐analysis

RVE was used to combine dependent effect sizes across the range of studies. The dependency is due to the inclusion of several outcome measures within the same sample, as is the case in seven of the included studies. Within this, a random effects model was chosen, as it assumes variability of populations and interventions in each study. The meta‐analysis steps are summarised below.

**Step 1:** Standardised mean difference (Cohen's *d*) and variance were calculated for each outcome measure using the escalc function within the metafor package (Viechtbauer, 2010). Full results are presented in Supporting Information: Appendix 14.

**Step 2:** To determine an overall effect size for the impact of digital devices on literacy and numeracy achievement when compared to ‘business as usual’ or other technology device, meta‐analysis was conducted using the RVE random‐effects model with correlated effects weights and small sample corrections, via the robumeta package (Fisher et al., 2017). Using correlated effects of 0.8 (Rho) and small sample correction, overall, the meta‐analysis found a positive, statistically significant effect size of Cohen's *d* = 0.24, CI = 0.07 to 0.40, *p* = 0.00848. This means that across the studies, children who received a maths and/or literacy intervention with mobile devices had better corresponding numeracy and/or literacy outcomes than children in control groups, who either used an alternative device (such as a laptop or desktop computer) or no device (class activities as usual).

Higgins and Green (2011) note that a value of *I* ^2^ greater than 75% demonstrates variance between studies. In this instance the overall *I* ^2^ value is large, *I* ^2^ = 89%, which means that the total amount of variance (sum of between study + within study) accounts for almost all heterogeneity in model. Reed et al. (2005) note that the heterogeneity of interventions is a common challenge for reviewers, particularly in the education field. There are many potential confounding variables, for example, age and gender of pupils, class size, teacher's personal delivery style, or available resources. As the goal of a systematic review is to compare and combine findings, this lack of homogeneity poses a challenge and means results must be interpreted with this in mind.

**Step 3**: Hedges et al. (2010) recommend that when using the RVE correlated effects model, sensitivity analysis is conducted to determine the effect of the value of Rho (size of within‐study correlation) assumed. Effect size was found to be consistent at all assumed values of Rho, which demonstrates that the results are not impacted by within‐study correlation.

**Figure 10:**
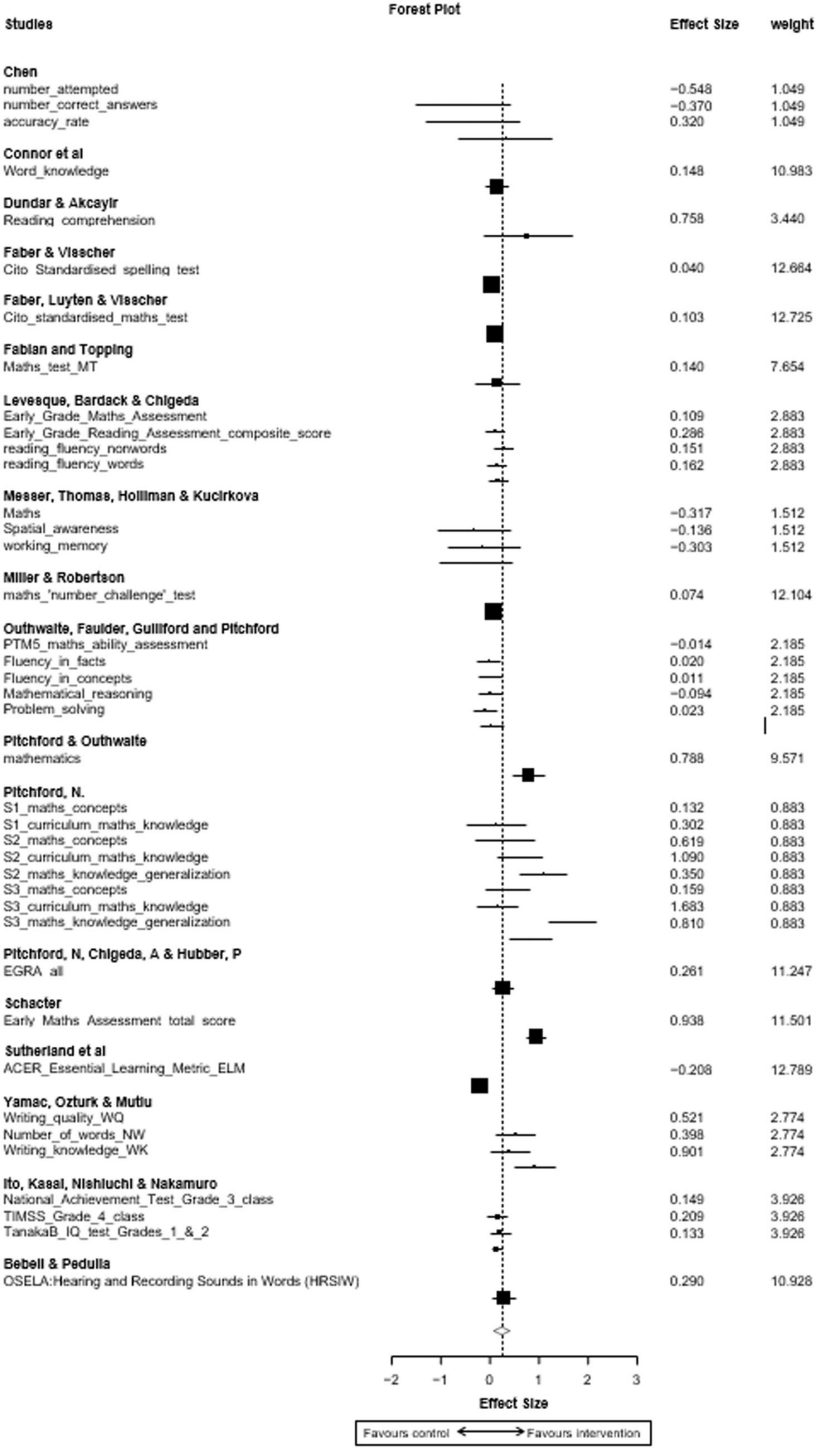
Forest plot – All studies.

**Stage 4:** Weighted effect sizes for each study, their corresponding confidence intervals, and overall effect size and confidence intervals were presented on a Forest Plot for RVE meta‐analysis, using the forest.robu function in robumeta (Fisher et al., 2017; see Figure 10). The forest plot for RVE differs from a traditional forest plot in that individual study weights are divided evenly across all effect sizes in that study.

#### 5.3.3 Moderating variables

To identify if any characteristics of the interventions had moderating effects on the intervention outcome, moderator/subgroup analyses was conducted via the robumeta package (Fisher et al., 2017) using a meta‐regression model with hierarchical effects weighting. The moderators (and corresponding variable codes) are summarised in Supporting Information: Appendix 15.

Where *df* < 4, findings cannot be trusted due to lack of power to detect statistically significant moderators. Therefore, while the meta‐regression model shows significant effects for two moderators (screen size (Cohen's *d* = 1.92) and SAMR level (Cohen's *d* = −0.76)), these findings cannot be relied upon. Additional studies are required to provide sufficient power to interpret these findings appropriately.

#### 5.3.4 Testing for publication bias

Publication bias occurs in the selective reporting of positive results. In small studies, larger effect sizes are required for significance, leading to potential ‘small‐study bias’ which can impact validity of the meta‐analysis (Marks‐Anglin & Chen, 2020). There is not currently a validated measure of publication bias to use alongside the RVE model (i.e., where there are dependent effect sizes), therefore effect sizes, and corresponding variance have been treated as independent. The following tests were applied with this caveat.

**Figure 11:**
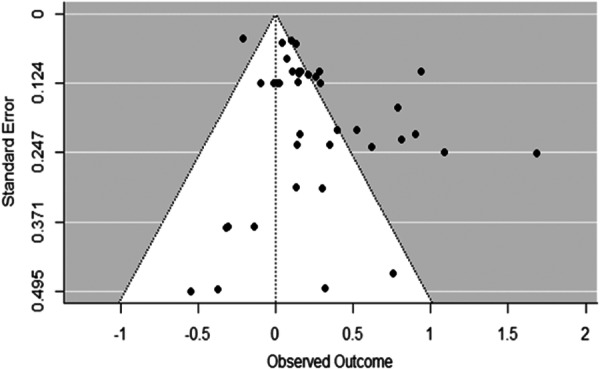
Funnel plot for publication bias.

To identify if there is publication bias present in the current review, a funnel plot (standardised mean difference against a measure of precision, in this case standard error) was drawn for included studies using metafor, and visually inspected for signs of publication bias (Figure 11). Where bias exists, the funnel will appear asymmetrical.

Studies on the funnel plot are somewhat unevenly distributed, with more studies appearing to have larger effect sizes and low standard error. However, this is a subjective assessment which should be confirmed using a statistical test. Egger's regression test is a linear regression of standard error and corresponding SMD's (Egger et al., 1997). If significance is reported (*p* < 0.05), asymmetry has been detected. While there are other similar calculations, Egger's method has been demonstrated to have higher power in meta‐analyses with less than 30 studies (Sterne et al., 2000). Egger's regression (mixed/random effects model) was conducted, and a non‐significant effect found (*p* = 0.50) therefore no asymmetry was detected.

As a final test for asymmetry, the trim and fill method (Duval & Tweedie, 2000) was used. This method trims the smaller studies leading to asymmetry, estimates the number of studies missing, and replaces the ‘trimmed’ studies and missing values in adjusted position assuming a symmetrical funnel plot. The model provides an estimate of number of missing values, and a new overall effect size, adjusted for publication bias. The output shows that zero studies were added, and a bias‐corrected effect size of 0.2574 was estimated (*p* < 0.0001) therefore still showing a positive, significant overall effect. Overall, the effect size appears robust against any potential publication bias. However, this result is interpreted with caution, as the method has been criticised as performing poorly in cases of high between‐study heterogeneity (Terrin et al., 2003).

#### 5.3.5 Sensitivity analysis for high risk of bias studies

Five included studies were rated as having potentially high risk of bias. To determine the impact of these studies on overall effect size, the calculations above were repeated with the five studies removed. The overall effect size decreases to 0.14 with 95% confidence interval of −0.01 to 0.28 and crosses the line of no effect therefore is not significant.

Repeating Egger's Regression Test for funnel plot asymmetry finds a significant effect of *p* = 0.05, suggesting some publication bias, while a significant bias‐corrected effect size of 0.2069 was found, which is closer to the original finding. This suggests that removing the studies with high risk of bias is moving us further away from the true effect size.

## 6 DISCUSSION

The search process identified 18 studies, all of which provided sufficient information to include in a meta‐analysis (with the omission of six outcome measures within one study due to insufficient information).

Using a range of RStudio packages, 40 dependent effect sizes were synthesised using a Robust Variance Estimator model, and an overall positive, significant effect size of Cohen's *d* = 0.24, *p* < 0.01, CI = 0.0707 to 0.409 with substantial heterogeneity (*I* ^2^ = 89%) was found. Moderator analysis was performed on six variables, and two of these (screen size and level of implementation on the SAMR scale) were found to be significant, however these findings cannot be trusted due to the low degrees of freedom (*df* < 4).

Publication bias was assessed using a funnel plot and Egger's Regression Test, and the trim and fill method used to correct for bias, with the overall effect size remaining positive and significant.

### 6.1 Findings in relation to the research questions

The primary research question asked the following:

- **What is the effect of mobile device integration in the primary school classroom on children's literacy and numeracy attainment outcomes?**

Based on the findings of 18 studies, including 40 outcome measures, a significant, positive impact of mobile devices on literacy and numeracy learning has been demonstrated (Cohen's *d* = 0.24, *p* < 0.01) for the included studies, meaning that for the children involved, there was an educational benefit to using digital mobile devices in the primary school classroom beyond traditional teaching methods or alternative technology interventions (non‐mobile devices). Given the range of interventions and study contexts, and the small number of reviews, wider conclusions on technology use in the classroom overall are not possible. In terms of magnitude of effect size, Cohen (1969) proposed a benchmark of 0.2 = small, 0.4 = medium and 0.8 = large to interpret values. However, many researchers, including Cohen himself (Cohen, 1988) have questioned the universal applicability of this benchmark across the full scope of research, suggesting that what may be considered a strong effect size will vary depending on the context. Within educational interventions, an effect size of 0.24 as found in this current meta‐analysis could be interpreted as reasonably substantial when considered in the context of the scope and frequency of the interventions (Higgins & Katsipataki, 2016). Indeed, a new benchmark for interpreting standardised effect sizes in education classes 0.20 and over as being a large effect (Kraft, 2020).

To support the accessibility and appropriate interpretation of effect sizes by those in a position to inform educational practice, the Education Endowment Foundation (EEF) Teaching and Learning Toolkit (Education Endowment Foundation, 2021) translates average effect size into a measure of additional months' progress made. Within this rating, an effect size of 0.24 is considered a moderate impact with an additional 3 months of progress gained (compared to those children who did not receive the intervention) (Higgins & Katsipataki, 2016). The effect size must also be considered in relation to the intensity and dosage of treatment, as low dosage interventions cannot realistically be expected to have large learning gains. This supports the earlier discussion by Selwyn et al. (2020) around unrealistic claims of ‘radical transformation’ by technology in classrooms. Technology is only one of a wide range of pedagogical tools that have the potential to enhance educational outcomes.

With regard to the included studies, the maximum treatment time was 120 h, however for 10 of the included studies, interventions were administered for less than 40 h, and one study only included 2 h of intervention. Considering that children may be in school for around 190 days per year, with approximately 5 h learning time per day, the interventions included could not be considered intensive. It would be unrealistic to expect that a short‐term intervention (e.g., 20 h across a school year) would yield a large effect size, therefore, in educational terms, a small significant finding is worthwhile. Indeed, several of the included studies found a small but not significant difference between control and intervention groups (favouring the intervention group), with authors reflecting that a longer intervention period may have led to a significant difference.

Additionally, in all cases, mobile device interventions were being compared to ‘business as usual’ teaching or an alternative technology intervention, rather than no teaching at all. This effect size therefore reflects additional learning beyond standard practice, rather than total learning. An overall effect size of 0.24 therefore provides evidence for the continued use of such interventions for literacy and numeracy teaching, however as discussed below, should be considered in context. Indeed, ‘business as usual’ varied from study to study. In some cases, there were similarities in activity – for example, where the control group undertook the same or similar problem solving activities or calculations using paper and pen/pencil, or read a similar story from a hard‐copy book. In other cases, detail on ‘business as usual’ was not provided, therefore no judgement was possible on whether the activities given to the control group were similar in content. The difference in intervention and control group should be an important consideration when implementing RCT design.

Five secondary research questions considered whether certain characteristics of the intervention were likely to impact efficacy, as follows:

- **Are there specific devices which are more effective in supporting literacy and numeracy? (Tablets, smartphones, or handheld games consoles)**

Using meta‐analytic regression, moderator analysis was conducted to identify if the type of device (as measured by screen size) had any bearing on the efficacy of the intervention. Only two interventions used devices with screens smaller than 7 inches (a small‐screen mobile phone, and a Nintendo DS Lite). While the analysis showed a significantly larger effect size for larger screens than smaller screens, the limited number of interventions disallows any conclusion to be drawn here on whether screen size was a significant moderating factor. Further research is needed in this regard. In addition, there may be differences in functionality and therefore in activities possible on smaller‐screened devices; consideration is needed as to their comparability. Additionally, while there were only two interventions identified which used smaller screened devices, it is also not possible to conclude that larger screened devices (usually tablets) are more popular in educational use.

- **Are there specific classroom integration activities which moderate effectiveness in supporting literacy and numeracy? (Aligned to the 4 stages of the SAMR framework (Puentedura, 2009) – substitution, augmentation, modification, redefinition)**
- **Are there specific groups of children for whom mobile devices are more effective in supporting literacy and numeracy? (Across age group and gender)**.

Similarly, moderator analysis was used to identify if the degree of intervention implementation (as defined by the SAMR framework) significantly moderated the efficacy of mobile device usage. Again, a significant difference in effect size was found for this factor, with higher values of the variable (representing a higher degree of implementation) having a larger effect on learning achievement. However as noted above, the sample size (*df* < 4) means that no conclusions can be drawn from this. Rather, the significant effect found may be due to a lack of power of the statistical test to detect the true size of the difference. A larger sample of studies may have produced different findings. This will be an important element of any update to this review and reflects the importance of further research in this regard.

Several other potential moderating factors were investigated, including gender and age of the pupils, literacy versus numeracy, and frequency of intervention, with no significant moderating effects found for these. As above, no conclusions can be drawn on this due to the small sample size, therefore further research is required before these potential moderating factors can be meaningfully explored.

- **Do the benefits of mobile devices for learning last for any time beyond the study?**

For most included studies, outcome data was only collected immediately post‐test, therefore it was not possible to draw any conclusions on the long‐term impact of the interventions where a significant effect was found. If learning gains compared to a control group are lost immediately after an intervention ends, this has important implications for ‘real‐life’ usage of interventions therefore should be a critical part of any research.

- **What is the quality of available evidence on the use of mobile devices in primary/elementary education, and where is further research needed in this regard?**

After a robust and systematic search process across a wide range of sources, only 18 studies were identified as being eligible for inclusion in this review. This indicates that overall, there is a lack of quality research on the topic. The inclusion of only RCT studies limited the search, however this was a considered choice to ensure only the highest quality evidence was included. Furthermore, the risk of bias assessment on the 18 included studies, clearly identifies that even for the robust studies identified, quality of reporting is in many cases a concern.

#### 6.1.1 Reflections on the interventions included in the studies

The included studies featured a wide variety of interventions. Some used game‐based formats, while others featured more traditional learning techniques in digital format. Using the SAMR framework (Puentedura, 2006) proved useful in classifying and comparing what were essentially very different interventions. By comparing the activities to the evidence on ‘what works’, it is possible to reflect on why some interventions may be more effective than others. However, this information is presented as a discussion rather than robust conclusions, again due to the limited number of included studies, and additionally, the complexity of factors at play within modern pedagogy.

##### Variability of impact

For many of the included studies, impacts of mobile devices varied widely across ages, class groups and outcome measures. While overall a significant positive impact has been demonstrated, this is far from the universal experience for all children involved. Bebell and Pedulla (2015) highlight the inherent complexity of educational technology within pedagogy, and therefore the need for a much more nuanced approach to both its implementation and study. A much closer investigation, through primary research and subsequent meta‐analysis, is needed to unpick whether mobile devices are more impactful for particular groups of children, for example, those with learning difficulties, those who have fallen behind their peers, or those from different socio‐economic backgrounds.

Several authors of included studies commented on their findings in relation to pupil subgroups and conducted some preliminary analysis. Faber et al. (2017) found a significant positive effect of the mobile intervention (Snappet for maths) for high‐performing students, although this may be explained by their ability to complete a greater volume of work than their peers in the allotted time. In contrast, Miller and Robertson (2011), Outhwaite et al. (2019), and Schacter and Jo (2017) found learning gains to be higher for low‐performing students, although these findings were not significant. There was not sufficient detail provided across the studies to allow for inclusion of these sub‐groups in meta‐analysis, however this is an area where further research and synthesis would produce important evidence for practice.

During the search process, there were studies identified which specifically looked at the impact of mobile devices to support those with learning difficulties to participate in mainstream classes, as well as studies considering how effective mobile devices may be as extracurricular activities to support those falling behind their peers. Several included studies reflected on individual child preferences in using mobile devices. Qualitative data collected by Fabian and Topping (2019) shows that children who found it easy to use the mobile device were more likely to enjoy the activity, and therefore to engage more, while those who struggled to use the mobile device were less positive and less engaged in the activity.

Expert Advisory Group members discussed the variability of impact demonstrated, and agreed that in practice, individual children often experience mobile device activities differently. They noted that this depends on the activity, however using tablets in class is sometimes more beneficial for pupils who struggle with traditional learning approaches, specifically because it uses a different set of skills, relies less on handwriting, and provides spell‐checking and formatting support as indicated below:

They all love using iPads, especially the children with special needs who can shine through it. Often what they produce is better than others when using iPads because they aren't hindered by spelling or writing skills. (Expert Advisory Group Member).

I have some children who don't show any flair, but you put them on an iPad, and they suddenly excel. And some of the applications produce a really high‐quality work with limited skills. (Expert Advisory Group Member).

Again, these are areas where further systematic review and meta‐analyses would build on the current study.

##### Heterogeneity of settings and activities undertaken

As already noted, the types of activities undertaken vary widely from study to study in intensity and complexity of intervention. The study by Bebell and Pedulla (2015) lasted for a full school year and evaluated an iPad implementation scheme that provided pupils with 1:1 iPad access however gave no detail on the specific activities undertaken. While the total usage hours is not provided, the intervention had the potential for significant iPad usage. In contrast, the study by Messer et al. (2018) considered the implementation of BeeBop, an iPad game requiring directions input to help a bee reach a flower, which was played for just 2 h in total across a 6‐week period (two sessions of 10 min per week). In terms of intervention dosage, these are the extremes found, with all other included studies falling somewhere between.

The remaining 17 interventions which specified the activities undertaken differed widely in their approach. Within the literacy subgroup, interventions included reading eBooks, watching instructional content before completing quizzes or comprehension questions, or undertaking more detailed writing assignments. Furthermore, within the eBook interventions there were notable differences. The study by Dundar and Akcayir (2012) simply compared the reading of a paper book with an eBook version of the identical text, with no added functions or features. Conversely, the eBook designed by Connor (2019) included a wide range of interactive and creative features, including a ‘choose your own adventure’ model, a built‐in dictionary, the ability to choose names for characters, and comprehension questions at the end of each chapter with feedback to check progress.

Connor (2019) found a significant positive effect on word knowledge, whereas Dundar and Akcayir (2012) found no significant difference. While it is not appropriate to directly compare the findings of these studies given that the age groups and frequency of interventions was not matched, the eBook used by Connor (2019) incorporated several additional learning tools which may have contributed to the significant findings. The ways in which these learning tools relate to the literature on learning are discussed further in the next section.

Expert Advisory Group members reviewed and discussed the interventions in included studies, and in particular reflected on whether the activities mirrored their own experience of mobile device interventions used in their classes. For the most part, group members had not heard of the interventions studied, although were able to name similar interventions which they had experience of using. Reflecting on the ways in which interventions were used in each study, the group felt that in everyday teaching, interventions in the included studies would not have been implemented in their classrooms in the way they were in the research. Rather, they would integrate a variety of interventions alongside one another, and with other teaching methods, as part of a wider lesson. Indeed, they felt this ability to use a variety of learning tools was one of the biggest benefits of mobile device use in the classroom as indicated in the excerpt below:

I'd rarely be giving iPads out to use an App; it would be more connected to the learning. For example, we used an ant simulator App and played that for a while, because we were studying mini beasts, so that allowed pupils to see things from the ant perspective, and then they used this to help in their story writing. So we played a game, but it was to write the story. The story writing was the main activity, not the App (Expert Advisory Group Member).

This feedback raises questions on the ecological validity of included studies, and the generalisability of their findings to everyday school practice. The debate on the potential for ecological validity in educational and other social research has been ongoing for many years, notably discussed by Bronfenbrenner (1977, p. 516) who defined ecological validity as ‘the extent to which the environment experienced by the subjects in a scientific investigation has the properties it is supposed or assumed to have by the experimenter’.

While all included studies were undertaken within the classroom and delivered in the most part by the teachers themselves, still consideration is needed in future research as to how the intervention might be used in ‘real world’ situations. The EEF Toolkit (Education Endowment Foundation, 2021), while summarising average effect size, also encourages users to look beyond the ‘headline’ to better understand how and why interventions have worked. Teachers are encouraged to look beyond the outcome/average effect size to consider value for money, and to draw on their own professional experience to consider applicability to their own context and improvement priorities for their school.

### 6.2 How do the findings relate to the existing literature?

The literature review above considered a number of elements which may moderate the impact that technology has on learning, or which themselves may be impacted by technology, which in term can support improved outcomes for children. The findings of both the qualitative analysis and the meta‐analysis are considered below in relation to the key areas outlined in the review of literature.

#### 6.2.1 Increasing motivation

As summarised above, the literature identified the elements of an educational intervention which are thought to increase motivation. These include autonomy and a sense of control; the opportunity for fun and creativity; immediate formative feedback; and the opportunity to collaborate (Deci & Ryan, 1985; Malone & Lepper, 1987). Indeed, Ciampa (2014) and others proposed that increased motivation may be a key factor in how mobile devices may support learning. The interventions in included studies, and the elements which may increase motivation, are discussed further below.

Many of the interventions supported autonomy by allowing children to work at their own speed through lessons and exercises, repeating any sections they were unsure of, and undertaking additional tasks if they had completed set assignments ahead of time. Authors noted how this model of practice differs from traditional class activities where children are usually working on the same exercise, led by the teacher, and are generally unable to skip ahead. Other interventions such as Maths Shelf (Schacter & Jo, 2017) and the ‘onebillion’ programme used in several studies (Levesque et al., 2020; Pitchford, 2015; and Pitchford et al., 2019) build on this autonomy function through adaptive assignments that match the skill level of the child, based on their performance in previous activities, therefore challenging each child at a level appropriate to their individual abilities.

Of the 18 included studies, 13 involved interventions that could be described as more ‘fun’ than their traditional counterparts. These interventions included game elements, attractive imagery, and interactive quizzes to keep pupils' attention and motivate them to continue learning. The ‘onebillion’ and ‘onecourse’ suite of activities, for example, as used in several included studies (Outhwaite et al., 2019; Pitchford et al., 2019; Pitchford, 2015; and Levesque et al., 2020) teaches both maths and literacy lessons with the help of animations, puzzles, and a cartoon character ‘teacher’ to guide activities.

Dr. Kawashima's Brain Training App, used in the study by Miller and Robertson (2011), is a commercially available game for home computers and mobile devices, and again includes an animated ‘teacher’ to lead the player through short game‐based puzzles and challenges. In comparison, while Snappet Maths, used in two studies (Faber et al., 2017 and Faber & Visscher, 2018) presents activities in a more visually creative way, activities resemble more traditional maths questions, rather than games. The ‘Explain Everything’ app evaluated in the study by Sutherland (2019) simply provided feedback on classwork, rather than employing any fun learning activities.

For the most part, children worked alone on their task, with collaboration with peers only facilitated in two of the interventions. The maths interventions in Fabian and Topping (2019) study (including Skitch and Pixel touch) required pupils to work in pairs with one shared tablet to identify, photograph and measure angles and lengths in real life objects. The Strategic Digital Writing Environment (SADIWE) literacy intervention studied by Yamaç et al. (2020) gave children the opportunity to work on their own essay assignments (on individual tablets) then share them with peers to give and receive feedback. Both studies gathered qualitative feedback from pupils on the experience.

Yamaç et al. (2020) reported that pupils found this collaborative element a useful learning tool, with friends or peers often giving them suggestions they would not have produced themselves. Fabian and Topping (2019) also reported that pupils recognised this benefit of working together, however some also found it difficult to work in pairs with a shared tablet, with disagreements on how to undertake the task, or one partner taking control of the tablet (particularly the case with mixed‐gender pairs). The eBook intervention studied by Connor (2019), also included an additional intervention condition where children took part in a book club for 15 min per day, allowing them the opportunity to discuss their reading and any challenges they may have faced. Although not relevant for inclusion in this meta‐analysis, the study found that the addition of this collaborative element brought significant positive benefits for pupils involved.

Expert Advisory Group members agreed that in their experience, collaborative opportunities were a crucial element in effective mobile device usage, however they also felt a device each, rather than shared devices, was more efficient as illustrated in the indicative quote below:

You nearly have more focused talk when they're working together from separate devices. They need to keep each other informed of what they're doing, whereas when they're sharing an iPad there was constant fighting over whose turn it was, or ‘you did that wrong’. They communicated more and stayed on task. (Expert Advisory Group Member).

Only one included intervention made use of social media to share children's outputs beyond their immediate peers, extending the collaborative approach far beyond what would normally have been achievable. SADIWE, developed and researched by Yamaç et al. (2020), supported pupils to upload their finished assignments to a class blog, which could then be viewed by friends in other classes; this had a motivating effect for pupils to do their best.

During discussions with the Expert Advisory Group, the opportunity to share completed classwork with a wider audience was also felt to be motivating. Advisory Group members found that existing digital home–school links established via the SeeSaw or Class Dojo school apps allowed pupil work to be shared in ‘real time’ with parents, encouraging live feedback, and prompting parent‐child discussions at home as illustrated below:

They could be sharing pieces of their work in real time [via Class Dojo], and parents could comment during the school day. Children sat a little taller, and this improved the relationships with parents and gave parents a small window into school life. I thought that was powerful used in the right way. (Expert Advisory Group Member).

Another said:

We do that via SeeSaw – they can upload bits and pieces, and this lets parents have something they can talk to their child about because they know what they've been up to. It gives them a conversation starting point. (Expert Advisory Group Member).

The introductory literature suggests that formative feedback, that is, feedback given during an activity with the aim to support behaviour change in real time, can support learning. Of the 18 included interventions, 12 provided some form of formative feedback, however, the type of feedback given varied considerably across the included studies. The ‘Explain Everything’ app, studied by Sutherland (2019) differs from all other interventions included because it is solely used to provide pupils with feedback on their work, and does not include any other pupil activity. This feedback is presented via video or voice recordings, or comments on work submitted digitally, and pupils can review it as required to inform their work.

In contrast, Yamaç et al. (2020) SADIWE creative writing app incorporates feedback from both teacher and peers within a wider learning activity. The pupils in this study found the opportunity for feedback useful, reporting that it increased their attention to detail and gave them fresh ideas to incorporate in their essays. Several studies also incorporated feedback to the teacher on student progress; Faber et al. (2017) note that this function gave teachers a better idea of the progress of their class activities, therefore actively informing teachers' response and content of/approach to future lessons.

#### 6.2.2 Theories of learning

Above, the key theories of learning are summarised, along with how these might apply to both pedagogy in the classroom and learning via mobile device interventions. Revisiting Supporting Information: Appendix 11 (characteristics of included interventions), there are elements of the key learning theories evident across included interventions, however the more complex interventions tend towards constructionism and social constructionism.

Repetitive learning is a feature of most of the included interventions (e.g., Think Think, onebillion and Maths Shelf), with reward or positive affirmation via on‐screen graphics or sounds when success is achieved, and ‘punishment’ via a negative sound or graphic and the need to repeat an activity rather than progress. This approach closely aligns to the core principles of behaviourism. Bebell and Pedulla (2015) note that while many educators assume that providing 1:1 access to iPads satisfies the conditions for child‐centred learning within a constructionist theory, in reality the critical point is how the iPads are used rather than their availability. All interventions but one (the Explain Everything App) actively encouraged autonomy, allowing children to work at their own pace and providing individualised feedback, as promoted by Piaget and others under a constructionist approach.

However notably, some interventions moved further towards a child‐centred approach by enabling children to work both at their own pace and individualised level of ability, building further on constructionist thinking and facilitating the Zone of Proximal Development (Vygotsky, 1978). This is demonstrated, for example, in Maths Shelf (studied by Schacter & Jo, 2017) which required children to first take a test to determine their current ability and therefore their appropriate starting position. Difficulty of activities then adjusted to suit individual progression. Some of the more complex interventions also demonstrated scaffolding through in‐App digital teachers and instructions, with children able to review varying levels of instruction until comfortable with the activity.

The ‘onecourse’ literacy intervention (studied by Levesque et al., 2020) provides a strong example of this scaffolded approach. An online character first explains a reading activity, then leads the child through the same story several times, first reading it aloud to them alongside pictures so they become familiar with the sounds, and gradually adding on‐screen text so they can follow along. Finally, the child has a chance to read the story without narration, touching on any words they do not understand to receive a prompt.

Constructionist theories highlight the importance of a ‘hands‐on’ approach to learning, for example, with physical exploration of objects central to an understanding of their properties. This theory was later developed more fully by Montessori and is an area where the touch‐screen functionality of mobile devices provides a benefit over traditional computers. Several included interventions made use of this functionality. In the intervention studied by Fabian and Topping (2019), pupils used a combination of Skitch, Pixel touch, and Measure Map and Area and Perimeter to photograph shapes in their school (outdoor) surroundings, then manipulate these images onscreen to allow them to calculate lengths, angles, and areas. While this type of activity would of course be possible without a mobile device, Fabian and Topping (2019, p. 10) emphasise that the mobile device made this activity more accessible and ‘seamless’, bringing the outside world directly into the classroom.

Interventions in the included studies tend to reflect quite individual learning experiences, with less evidence of opportunities for social learning and collaboration. As noted in the review of literature, the role of collaboration has been highlighted by more recent educational theorists such as Freire and Dewey who stressed the importance of active enquiry and social engagement within a participatory pedagogy. Only two of the included interventions can be said to fully support collaboration and social enquiry. Fabian and Topping (2019) intervention (discussed above) was conducted in pairs and required physical exploration of the surrounding area to identify appropriate shapes, therefore encouraging active (both mentally and physically) enquiry. Additionally, the SADIWE writing intervention studied by Yamaç et al. (2020) incorporated opportunities throughout for children to collaborate with one another, to research their essay ideas online, and to creatively present their work for peers. This intervention clearly aligns to the principles of social constructionist theorists and provides a more immersive learning experience when compared to other included examples of interventions.

#### 6.2.3 Level of implementation: The SAMR framework

The discussion so far points to a complex and varied picture of mobile device use in the primary classroom, both in interventions used (and the elements of each), and their effects on learning. The SAMR framework (Puentedura, 2006), summarised in the literature review provides a useful classification to enable comparisons between these different interventions, however, also encourages those reviewing the literature to take a closer look into the operational elements of an intervention, raising the question of ‘why do they work’ rather than just ‘what works’. As already noted, there were not sufficient studies to draw robust statistical conclusions on the moderating effect of the level of implementation of interventions (as judged by the SAMR rating), and it is important that this analysis is revisited in the future if and when a larger sample size of appropriate research is available.

However the included studies, rated by the Expert Advisory Group members under the SAMR framework, provide evidence of the types of interventions available, the common elements within, and how they might change the way children learn and engage in class. When designing literacy and maths mobile device applications, there is evidence of effort being made to use the potential innovative elements to expand what is possible in the classroom, as demonstrated by 15 of 18 included studies which incorporated interventions classed as either augmenting or modifying existing class activities. Only one intervention (Dundar & Akcayir, 2012) was considered to be simply a substitution for the usual activity, and one intervention (Yamaç et al., 2020) was considered to fully redefine class activities. Examples of the types of activities involved are discussed below.

The reading intervention assessed by Dundar and Akcayir (2012) compared the accuracy of a child's reading aloud from a paper book or from an eBook. The eBook had no additional features, therefore simply *substituted* one medium for another. Chen (2014) ‘Brain Challenge’ game, classed as *augmenting* existing practice, was played by pupils for 15 min each day, and included a selection of puzzles and tasks. While a paper and pencil version of the same puzzles could have been administered, the mobile app added several features to what was previously possible, including immediate feedback on answers (right or wrong) without the need for intervention by the teacher, and higher levels to be unlocked on achieving a certain score or level, therefore encouraging repetition for learning.

The ‘onebillion’ programme, used in several studies, was rated as a *modification* of ‘normal’ practice. Children engaged in interactive lessons, gaining exposure to new learning materials and concepts in a fun way, but importantly at their own pace. Several studies demonstrated the use of this intervention in developing countries, where often children were in large classes with widely mixed age and abilities. While it would not be possible for the teacher to provide instruction and feedback at the appropriate level for each child, ‘onebillion’ facilitates individualisation of the learning process with minimal teacher time required. The teacher is therefore free to monitor individual progress in real time (a further feature of the intervention) and provide one to one support to children where required. Maths Shelf, used in the study by Schacter and Jo (2017), was also rated as *modification*, and incorporated similar features to ‘onebillion’. Again, individualised learning was facilitated with a pre‐test to identify an appropriate starting level for each child, and immediate feedback was given on performance to allow the child to review or practice the areas they were less confident in.

Only Yamaç et al. (2020) essay‐writing intervention was rated by the Expert Advisory Group as a *redefinition* of classroom activities. As in the interventions above, children were able to work autonomously, engage with instructions through the App on new topics relevant to the area of study, and receive timely feedback on their work. Beyond this there were several elements of the activity which elevated it far above standard ‘paper and pencil’ essay‐writing practice. These included the opportunity to research the essay topic on the internet before starting; the options to include pictures and multimedia features in the final essay presentation; the opportunity to watch instructional videos on each element of the writing process; the ability to plan, rearrange, and redraft writing without the mess that erasing paper and pencil would make; a chance to share draft essays with peers and give/receive feedback on these; and the use of social media to disseminate completed essays to peers, friends, and families.

The study by Yamaç et al. (2020) also included qualitative feedback from pupils, who reported the above elements to be motivating and supportive of learning. This intervention also best matched the Expert Advisory Group members' reflections on the types of approaches used in their own and colleagues' classrooms when using mobile devices to support learning, given the interplay of several different activities. Across the sample of included studies therefore, there is a trend towards modification of traditional classroom practice using the innovative and creative tools available with mobile devices. As the sample was small, it is not clear if this observed trend is a true representation of general practice in primary schools, however the SAMR framework provides a useful framework for both future intervention development and implementation, and for research.

#### 6.2.4 Teacher skills and knowledge: The TPACK framework

A further factor identified in the literature review as having the potential to moderate the effect of mobile devices on learning is the skills, knowledge, and experience of the teacher in the relevant technology, and their ability to combine this with their subject and pedagogical knowledge to effectively support learning. The TPACK Framework (Mishra & Koehler, 2006) proposes that it is at the intersection of these three areas of knowledge (subject, pedagogy, and technology) that the most effective teaching takes place. Expert Advisory Group members saw this combination of skills and knowledge as key as highlighted in the indicative quote below:

Using mobile devices in the classroom is multi‐dimensional, and totally depends on teacher competence and their understanding of pedagogy and how iPads can support that. They may be able to accidentally improve, but it takes real understanding to properly make a difference … it is only as good as the person leading it. (Expert Advisory Group Member).

It all depends on the teacher standing in front of them, their confidence, and skills in using the technology, and their ability to model practice. (Expert Advisory Group Member).

While it can be assumed teachers have a substantial level of knowledge in their subject area and general pedagogical practice, knowledge of the mobile device, and the intervention, is a new area for most and therefore a gap in the TPACK model which must be filled through training and capacity building. In this regard, pedagogical knowledge must also incorporate a knowledge of when technology is appropriate at all, or indeed which of the SAMR degrees of implementation is most relevant to both the subject and the individual children/context at the time. Under many circumstances, traditional teaching methods may in fact be the most appropriate.

The majority of included studies incorporated teacher training within their methodology. Levesque et al. (2020) provided teachers involved in delivering and/or supporting pupils during the intervention with 8 h each of technical training to ensure they were competent in the use of the device (iPads), the software (onebillion reading application) and the practicalities of administration (e.g., the pupil registration process). Teachers also had an opportunity to practice using the software and troubleshoot before pupils were engaged. Similarly, Schacter and Jo (2017) gave teachers 2.5 h of training on the relevant maths app (‘Math Shelf’) to support their understanding of the content and learning approach. In this study, the control group also received training, however the intervention group received an element of training focused on technology, which the control group did not receive. Meanwhile, Miller and Robertson (2011) provided 1 h group training sessions. Faber and Visscher (2018) and Faber et al. (2017) provided two training sessions for teachers, introducing teachers to the software, and facilitating understanding of how to integrate the App (Snappet) effectively into lessons. They also gave teachers optional access to a Snappet coach via telephone to consult as and when required. Training in these studies was not extended to, or relevant for, control group activities.

Several studies include a discussion of relevant teacher skills and knowledge in the interpretation of findings, and the interplay between technology and teaching. Connor (2019) incorporated a teacher‐led book club intervention group in their study, which gave pupils the chance to discuss their reading with their teacher and peers, and brought greater learning effects than the reading intervention alone. The authors note ‘Increasingly, we are discovering how technology that is designed to provide learning opportunities that complement and enhance teacher–student interactions is generally more effective in supporting learning than technology alone’ (Connor, 2019, p. 299).

The TPACK Framework raises questions around the ecological validity of research where the intervention is administered by a researcher without the necessary subject or pedagogical knowledge. This was the case in several included studies (e.g., Dundar & Akcayir, 2012) while others used a combination of researchers and class teachers for delivery (e.g., Connor et al., 2019). Outhwaite et al. (2019, p. 286) note the importance of teacher‐administered interventions for future research to support ecological validity and generalisability of findings. However, this must also be balanced with discussions on the potential risk of bias in teacher‐administered interventions, particularly where the same teacher supports both the intervention and control groups. Craven et al. (2001) discuss potential ‘diffusion effects’ whereby some learning from the intervention group may affect control group outcomes, either because pupils share their learning with peers, or the teacher unintentionally (or intentionally) transfers learning between groups. This potential ‘contamination’ was noted as a limitation in the included study by Sutherland (2019), where the same teacher participated in both control and experimental group delivery.

Finally, Expert Advisory Group members reflected not only on the complex persona of ‘teacher’ and the multiple roles this incorporates, but on the Hierarchy of Needs theory (Maslow, 1943), which proposes that children cannot learn (‘self‐actualisation’) unless their basic needs have first been met as indicated in the quote below:

Teaching is so complex. It's about relationships, and things like ‘has the child had breakfast’. (Expert Advisory Group Member)

As with all learning, with and without mobile devices, the wider context of the child's life and the issues they are facing will impact their outcomes, and teachers must combine all of their knowledge and skills to effectively support learning. Clearly effective mobile device interventions must incorporate an element of teacher training and support, however the critical elements and extent of support required is complex, and conclusions cannot be drawn from the small number of studies included in this review. Again, this is an area where future research might usefully focus.

### 6.3 Summary of main results

The search process identified 18 studies, all of which provided sufficient information to include in a meta‐analysis (with the omission of six outcome measures within one study due to insufficient information).

Using a range of RStudio packages, 40 dependent effect sizes were synthesised using a Robust Variance Estimator model, and an overall positive, significant effect size of 0.24, *p* < 0.01, with substantial heterogeneity (*I* ^2^ = 89%) was found. Moderator analysis was performed on six variables, and two of these (screen size and level of implementation on the SAMR scale) were found to be significant, however these findings cannot be trusted due to the low degrees of freedom (*df* < 4).

Publication bias was assessed using a funnel plot and Egger's Regression Test, and the trim and fill method used to correct for bias, with the overall effect size remaining positive and significant.

Overall, this systematic review and meta‐analysis demonstrates that mobile devices can support learning in literacy and numeracy, within certain contexts and conditions, and provides evidence to inform the direction of travel for both research in the field, and practice development. This is currently one of only a small number of robust systematic reviews and meta‐analysis on this area of educational practice; therefore, the findings should be of particular interest to educational policy makers, practitioners, researchers, and indeed educational programme designers. Specific implications are detailed below. However, findings should be interpreted with caution; Higgins and Katsipataki (2016, p. 237) note that a meta‐analysis tells us ‘what has worked’ rather than ‘what works’. Educational context varies vastly from country to country, school to school, and from pupil to pupil. Therefore, while an overall effect size is a useful tool to guide practice, this must be interpreted within the framework of local knowledge and practitioner experience.

### 6.4 Overall completeness and applicability of evidence

The main limitation to note in assessing the completeness and applicability of evidence is the small number of studies identified for inclusion in the review. While the inclusion of only RCTs aimed to ensure the inclusion of only robust studies, this limited the total number of included studies (18 overall). While the identification of an overall effect size was possible, the small number of studies meant it was not possible to answer the follow‐up research questions on the moderating factors, which would have supported better understand of how and why mobile devices might support learning outcomes. As reflected on above, the geographical coverage of studies was also limited, and there are several studies by the same authors. Furthermore, there was insufficient follow‐up data reported to allow conclusions to be drawn on the longer‐term impact of any learning gains identified. If learning gains compared to a control group are lost immediately after an intervention ends, this has important implications for'real‐life’ usage of interventions therefore should be a critical part of any research. Researchers should therefore consider collecting and reporting longer term data where possible to support a more detailed picture of intervention impact.

### 6.5 Quality of the evidence

Reporting of methodology detail was limited for many of the studies. For one of the included studies (Bebell & Pedulla, 2015) analysis was only reported for one outcome measure, which had a significant positive effect, while several other outcomes had been assessed in the study. This raises concerns of reporting bias and may have artificially increased the effect size. Beyond this, the assessment of a medium or high Risk of Bias across all studies reflects a larger issue regarding an overall lack of reporting of adequate detail to enable an assessment of quality to be made. Research methodologies may have been implemented rigorously but for the large part it was not possible to confirm this. Reporting on the randomisation process was of particular concern, given that for 12 studies, it was not possible to determine whether randomisation was conducted appropriately; these studies simply reported that ‘randomisation took place’ rather than providing detail on the method. Lack of blinding was also a concern in several studies, particularly where the teacher was also the assessor. While this is not a large concern where tests have a straight ‘right or wrong’ answer, this may have introduced opportunity for observer or confirmation bias where assessors had a subjective decision to make on pupil performance in a test, as was the case in studies by Dundar and Akcayir (2012) and Yamaç et al. (2020). Furthermore, as teachers were aware that they were taking part in a trial, they may have adjusted their wider behaviour, consciously or unconsciously, to improve class results.

Authors of one study (Miller & Robertson, 2011) discussed the possibility that teachers in the control group may have adjusted their ‘normal practice’ to compare more favourably with the intervention group (known as the ‘John Henry Effect’), however this may also have been a possibility in several other studies too. While it is difficult to conduct a fully blind study within educational research as interventions take place within classroom settings and are often administered by the teacher, there are some possible steps to take to minimise risk of bias, for example, in the use of external assessors who are blind to allocation. The publication of a pre‐specified protocol and analysis plan would also provide a further guarantee that reasonable steps have been taken. Again, this is a consideration for future educational research.

### 6.6 Potential biases in the review process

The systematic nature of this review means that it provides a robust overview of existing studies. However, there are several limitations which should be considered in the interpretation of the findings presented.

#### 6.6.1 Number of included studies

The main limitation of this study is the overall lack of robust research on the use of mobile devices in the classroom to support the teaching of maths and literacy. The meta‐analysis is based on 18 studies, which is a small sample, and doesn't allow for robust conclusions to be drawn. Within the 18 interventions identified, some had very small sample sizes (the lowest being 12). Widening the search to include quasi‐experimental studies would have drawn a wider selection of studies, however, would have lowered the robustness of included evidence. Considering that tablet implementation is a priority across many schools, and that investment is being made in devices and in teacher development, it is critical that more rigorous research is conducted on the subject to ensure that this investment is actually benefitting children's achievement. However, it is important to recognise that RCTs may not always be relevant in educational settings, and other research designs may be more appropriate. Qualitative research also provides useful context, and was not included in this review. Reproducing this systematic review in the future, perhaps widening the scope in terms of types of study if capacity allows, would include a wider number and range of studies and build on the evidence presented here.

#### 6.6.2 Lack of consistency in control activities

Given the range of activities showcased in the interventions, there is an equally diverse range of activities undertaken as control group activities. This reflects real‐life practice in classrooms where ‘business as usual’ will be wide‐ranging. It was therefore not possible to consider equivalence of control group across studies. As noted in the methods above, control group activities were either traditional teaching methods or activities using alternative technology (such as desktop computers). In each of these cases, the aim was to determine the additional learning benefits afforded by the functionality of the mobile devices. In the future, where more studies are available, closer analysis of the types of control interventions will be of interest.

#### 6.6.3 No studies identified in languages other than English

While language was not included as a limiter in the search process, and several included studies are from non‐English‐speaking countries, nevertheless it is important to reflect that the search strategy included only English search terms, therefore may not have identified relevant studies in other languages. Six countries were represented in the final sample (USA, UK, Turkey, Cambodia, Malawi, Netherlands). Since research has shown the breadth of mobile device usage across the globe, research from a wider range of countries should have been expected. However, Dietrichson (2020) recently completed a similar systematic review on an element of literacy and numeracy interventions, also finding a limited geographical spread in included studies (71 studies from only six countries, including USA, Canada, UK, Germany, Netherlands, and Australia). The fact that many countries are not represented in the final sample may be explained by several factors, for example, a lack of rigorous educational research from those countries, lack of mobile device usage in education, or limitations in the search strategy. Various sources report that English is the most common publishing language even for those for whom it is not their native language (e.g., Di Bitetti & Ferreras, 2017; Hamel, 2007). Additionally, publishing criteria for journal articles in languages other than English often require an English language title and abstract (this is a minimum criterion in Scopus publishing (Elsevier, 2020)). While it is likely that language hasn't limited the identification of published articles, this may be a source of bias for non‐published articles, as identification of these relied on Google Scholar searches.

#### 6.6.4 Limited focus on literacy and numeracy in primary school education

Had the review been conducted by a larger team, the scope would have been broadened to encompass all studies using mobile devices in primary and post‐primary schools. During the search process, examples of interventions to support teaching of a range of subjects were identified, including ‘the world around us’, science, music, and art. Expanding the remit of the review would have provided a much richer insight, and should be considered in any future replication or extension of this review. However, primary and post‐primary education differ in important ways, and therefore separate analyses are required to identify the critical elements of each.

#### 6.6.5 Limitations of search approach and timeframe

The searches for this study were undertaken between October and November 2020 and have not been updated before publication, therefore the authors acknowledge that further relevant studies may have been published in the interim. An updated search would be useful and timely. The authors also note that although the search criteria and approach was designed to be comprehensive and sensitive, is is possible that the approach failed to identify relevant references. In particular, while care was taken to include search terms reflecting the broad descriptors for randomised controlled trials, it is possible that some authors did not include such terms in their abstract and again, relevant studies may have been missed.

### 6.7 Agreements and disagreements with other studies or reviews

While there are no systematic reviews focused specifically on the same target population and subject area, as already discussed, there are others on a similar broad theme. Dietrichson (2020) reported on average a positive, significant effect of targeted school‐based interventions for literacy and maths (including some using technology). Savva et al. (2022) found a small, positive effect for electronic storybooks on language and literacy outcomes for children aged 3 to 8. Meanwhile, Kim et al. (2021) found similarly small, positive effects for educational apps to support literacy and numeracy in children aged 3–9, but reflected on the wide range of apps available and the need to consider quality of app design and the learning principles behind the activities. Indeed, a recent content and qualitative comparitive analysis of educational maths apps undertaken by Outhwaite et al. (2023) stresses the importance of considering the design features and breadth of mathematical skills covered when assessing the effectiveness of educational maths apps. It is not appropriate to directly compare this current study with others, given the wide range of interventions and differing populations, however when considered alongside other reviews on the theme, there is growing evidence that mobile devices, when used in an informed way, can positively impact learning. Yet much wider knowledge and understanding is needed on the design of these apps, and how and why they are effective, under which circumstances.

## 7 AUTHORS' CONCLUSIONS

### 7.1 Implications for practice

Within primary or elementary education, literacy and numeracy are core life‐skills which underpin the learning of all other subjects and are central to achieving wider life outcomes. The use of mobile devices, in particular tablets, is growing in primary schools globally, yet to date there is limited detailed knowledge of the impact these can have on learning, and how they can best be used to support learning. This robust systematic review and meta‐analysis, conducted to the standards set by the Campbell Collaboration, is the first undertaken in the use of mobile devices in mainstream primary school classrooms to support literacy and numeracy, and has demonstrated a significant, positive effect of these devices. Moreover, the review included only Randomised Controlled Trials, meaning that the meta‐analysis findings can be considered amongst the highest standards of evidence of effectiveness, with relevance beyond the included samples.

The review has uncovered issues for consideration by researchers undertaking work in this area in future, including the need for more RCT studies in this field; the importance of clear reporting to support interpretation and application of findings, and indeed meta‐analysis; and the potential moderating factors which should themselves be the focus of future research as researchers and practitioners collectively seek to better understand and use this innovative resource.

#### 7.1.1 Implications for educational practitioners

Systematic reviews have a key role to play in supporting the ‘evidence‐based practice’ movement, particularly in education, where they can bridge the gap between research and real‐world practice (Davies et al., 2000). This review presents not only positive overall findings to support the continued use of mobile devices in literacy and numeracy education, but also highlights the need to pay much closer attention to how and when they are used.

This is an extremely timely review, and the lessons learned have immediate implications for practice given the impact of COVID‐19 on primary and post‐primary education. Children across the globe spent a considerable proportion of the 2020–2021 academic year learning at home, and sporadic disruptions continued into 2021–2022. During this time, practice has varied from school to school, with some schools making use of online activities more than others.

Although classroom lessons have resumed as normal, mobile devices look set to be a permanent fixture in many classrooms. Therefore, a strong understanding of the benefits of mobile technology for education, and how this can best be implemented, has immediate relevance to educators globally. While the current systematic review focused on classroom‐based activities, the lessons can also be translated to the ‘online classroom’ for whole‐class learning. Teachers must be able to access the information on ‘what works, for whom and how’ regarding online learning, and be given the tools, training, and professional support to use this in the real world. Systematic reviews play a key role in making this evidence accessible, and the content and implications of this review should be of interest to many teachers.

#### 7.1.2 Implications for educational resource developers

This review demonstrates wide‐reaching educational benefits to be gained from the careful and informed development of mobile device software to support literacy and numeracy learning. However, the content of this software and its mode of implementation must be led by the evidence on the mechanisms which support learning, and how those can be embedded in the intervention design. Additionally, literacy and numeracy are multifaceted, and interventions can be tailored to support one or several of these elements.

Therefore, a combination of ‘what works’ in literacy/numeracy teaching, ‘what works’ in mobile device interventions, and wider pedagogical practice, should be considered by those seeking to develop effective educational technology. Important implementation considerations include dosage, instruction manual/guidance, teacher training and technical support needed. Finally, resource developers must commit to the evaluation of innovative technologies, in real‐world settings, to robust standards, to ensure that practice can be truly evidence‐informed and makes a positive contribution to outcomes for all children.

### 7.2 Implications for research

This review has firstly demonstrated the importance of research in this area, given the potential to support literacy and numeracy development. It has also illuminated several areas for consideration by those undertaking research in the future, as summarised below.

There is a general need for more research in this area. The robust systematic search process in this review has revealed a significant lack of quality research in this area, with only 18 studies identified for inclusion. This is of significant concern, given the extent to which mobile devices are now being used in education, and particularly considering the blended face to face/online approach to education that has been necessary since the beginning of the COVID‐19 pandemic. A central recommendation stemming from this review is therefore the need for prioritisation of this area in the educational research agenda, nationally and globally.

Furthermore, there is a need for a more nuanced approach to research in this area which preserves ecological validity. Research undertaken in this area must be carefully designed to take account of the wide‐ranging moderating factors at play in using mobile devices in the classroom, including the intensity and approach to implementation, the impact of teacher skills, knowledge and attitudes, and the degree to which mobile devices can redefine what has previously been possible in the classroom. Only one included study (Bebell & Pedulla, 2015) evaluated the impact of a specific iPad implementation scheme, therefore was designed around the aims of the scheme. In most other studies, the intervention was implemented purely to facilitate research, therefore it could be argued the interventions were less akin to real life practice. Bebell and Pedulla (2015, p. 212) reflected on the importance of schools getting on board with the need for educational research, given the rapidly changing technology and desire to embed it in everyday teaching, noting ‘schools themselves need to become increasingly comfortable and conversant with educational research and evaluation opportunities.’ Research and evaluation should go hand in hand with mobile device implementation in education, therefore ensuring that investment made is actually making a difference to children.

There must also be careful consideration of the ethics of educational research. Robust research of this kind requires a randomly allocated control and intervention group, meaning that one group of participants takes part in the research for little obvious benefit. While this is not essentially ‘doing harm to’ participants, the ethical implication of withholding a potentially beneficial intervention from control group members is an ongoing debate by those seeking to undertake RCTs in social research. Fives and Gill (2015) summarise the arguments in this regard, noting that central to informed consent is an understanding by the participant of their contribution to the greater good, even if they themselves will not benefit. While the debate continues, so does the need for robust research in education, therefore steps can be taken to support positive ethical practice, as evidenced in several of the included studies which provided a delayed or ‘waitlist’ intervention option for those children in the control group.

Finally, the process of assessing risk of bias in included studies highlighted several practical issues in the robust reporting of educational research, as well as a wider need for careful and detailed reporting of the procedures undertaken to minimise bias. There are of course areas in educational research where risk of bias cannot be fully removed, for example, in the difficulties of conducting double blind experiments in classroom settings, however steps can be taken to minimise or mitigate risk. Failing that, an open discussion on potential shortfalls of the research adds to the trustworthy nature of the research and allows others working on similar studies to adjust their own practice where possible.

### 7.3 Conclusions

This review sought to investigate the effectiveness of mobile devices in literacy and numeracy education in primary school classrooms. Systematic methods were used to conduct a thorough, transparent, and replicable search of all available research to allow robust synthesis of effect sizes through the most up‐to‐date meta‐analytic techniques. Although based on a small sample of studies, several conclusions can be drawn from this systematic review and meta‐analysis.

Overall, this review has shown that mobile devices are effective when used in the classroom to support literacy and numeracy learning, with a positive significant overall effect found. The use of such devices is complex, and the findings should be interpreted alongside wider knowledge of pedagogy and intervention implementation. The review also highlights concerns on risk of bias in the included studies. Little is known about the longer‐term effect of these devices as there is limited data collected and analysed at follow‐up time intervals post‐intervention.

Robust research on technology use in the classroom is currently extremely limited and is a critical area for focus in the future, given the rapidly increasing use of such devices in teaching. This lack of research means that it is currently not possible to draw conclusions on potential moderating factors within interventions, which is critical for future practice development and will help to answer questions about why and for whom mobile devices work.

This is an emerging area of practice, and therefore of research. As investment in mobile devices grows, and as primary schools look to more innovative methods to support learning in the core skills of literacy and numeracy, so research in this area must be prioritised. Mobile devices have immense potential, and research is the key to fully understanding how best to invest, who can benefit most from new innovations in learning, and how they can most effectively be combined with existing good practice to ultimately support children's educational, and wider, outcomes.

In future, emerging research should be monitored, and this systematic review and meta‐analysis repeated when appropriate to capture emerging technologies and practices and ensure up to date evidence is available to policy makers, practitioners, researchers, and all those with a role to play in supporting outcomes for children. There is no doubt that children can benefit from continued use of mobile device interventions, however a clear understanding of how and why they are effective will optimise the learning experience and outcomes for all.

## CONTRIBUTIONS OF AUTHORS

This systematic review and meta‐analysis formed the basis of Claire Dorris's Doctoral Dissertation, undertaken via the DChild course at the School of Social Sciences, Education and Social Work at Queen's University, Belfast, with support from her supervisors, Prof. Karen Winter and Dr. Liam O'Hare, and from Prof. Edda Tandi Lwoga at the College of Business Education, Dar es Salaam, all co‐authors on this review.

Claire was primarily responsible for all aspects of the review, while co‐authors provided support at all stages, including developing and implementing the screening process, data extraction, risk of bias assessment and wider editing.

## DECLARATIONS OF INTEREST

There are no conflicts of interest noted for any of the authors.

## SOURCES OF SUPPORT

### Internal sources

- No sources of support provided.

### External sources

- No sources of support provided.

## DIFFERENCES BETWEEN PROTOCOL AND REVIEW

For the most part, the protocol was adhered to, however four minor deviations were required. Firstly, a date limiter of 2010 was applied to searches to increase specificity and ensure that only ‘new technologies’ were identified. Meta‐analysis was undertaken via RStudio software rather than Rev Man. This provided a wider suite of tools to undertake a more detailed analysis. Thirdly, the protocol stated that where the population studied includes children outside of the specified age group (4–11 years), contact would be made with the author/s to determine if disaggregated data was available. However, in some cases, class groups of interest included 12‐year‐olds, and it was not possible to exclude them from the analysis. It was therefore decided that 12‐year‐olds should be included in the desired population. Finally, one additional moderator (age of children) was included in subgroup analysis, as already detailed above).

## Supporting information

Supporting information.

Supporting information.
