# Supplementary material for: A systematic review of mobile device use in the primary school classroom and impact on pupil literacy and numeracy attainment: A systematic review
Source: Campbell Syst Rev. 2024 Jun 20;20(2):e1417. doi: 10.1002/cl2.1417 (PMC11190352; doi:10.1002/cl2.1417)
Supplement: Supplementary file 1 — Supporting information. [file CL2-20-e1417-s002.docx]

# Characteristics of studies

## Characteristics of included studies [ordered by study ID]

#### Bebell and Pedulla, 2015

| ***Study characteristics*** | |
| --- | --- |
| Methods | Cluster RCT - class-level assignment |
| Participants | 266 Kindergarteners: 8 classes with 129 iPad students; 8 classes with 137 comparison students. Children aged 5 |
| Interventions | 1:1 iPad access with the intention of supporting literacy and numeracy attainment- numerous apps were accessed, rather than the study testing an individual intervention.  Students used the ipads for 12 weeks, however the experiment tested them at 3 weeks and 12 weeks. |
| Outcomes | Literacy- Traditional ELA assessments used-, inlcuding Rigby Benchmark Assessment, Children's Progress Academic Assessment (CPAA), Observation Survey of Early Literacy Achievement (OSELA). |
| Notes |  |

#### Chen, 2014

| ***Study characteristics*** | |
| --- | --- |
| Methods | RCT - individual randomisation |
| Participants | 17 (14 boys, 3 girls) children from one class in a primary school. Children were randomly assigned to group A (9) or B (8). T-test showed no significant diff between the 2 groups before intervention. |
| Interventions | Brain Challenge'mobile phone game with four different tasks- logical, memory, visual & maths. Games 'Trout Route' & Arithmetic were used as the intervention. Children played Trout Route 5 times before Arithmeteic was unlocked. Each game allowed 90 secs to answer the problems. Game record sheet completed to record how often children played & their high score each time. |
| Outcomes | 3 measures developed (Mental Mathmatics Challenge) and based on the Scottish maths curriculum for the correct age group. Items included addition, subtraction, multiplication & division. Each test had 60 items & was marked right or wrong, thereffore scored out of 60. Time to complete the papers was also recorded, with max 15 minutes. Mean accuracy rate calculated. |
| Notes |  |

#### Connor, 2019

| ***Study characteristics*** | |
| --- | --- |
| Methods | Cluster RCT - class-level assignment |
| Participants | 3rd - 5th grade pupils in 2 primary schools, mean age 9.5. 603 total sample, 49% female, 67% Hispanic. 70% qualified for school lunch programme. 391 exp group (191 book club), 212 control (103 book club). |
| Interventions | Self-developed ebook for tablet reading, with 'choose your own adventure' theme, built in dictionary and understanding tests with feedback. Half of each experimental & control group also took part in a weekly 15 min book club to discuss their reading and strategy (not included in the review). |
| Outcomes | Self-designed comprehension test: 7 items to test summarisation skills, word knowledge and learning strategies (e.g. asked how they learned the word). Word knowledge task: word matching, synonyms and figuring out the meaing from the wider context.  Standardised readign/vocabulary test: Gates MacGinitie Reading Test (vocab and comprehension). Pre, mid & post-tests- mid used for RCT findings (prior to delayed group intervention) |
| Notes |  |

#### Dundar and Akcayir, 2012

| ***Study characteristics*** | |
| --- | --- |
| Methods | RCT - individual randomisation |
| Participants | 20 5th graders in one school (10 each in control & experimental group) |
| Interventions | Reading a text on ipad vs reding the same text on paper. Children read aloud to teacher/researcher who noted errors. |
| Outcomes | Akyol's chart for understanding, with researcher or teacher grading answers for fully, partially or not answered.  To measure speed/accuracy, students read a passage aloud for a set period, & researcher counted the number of accurate words, then converted to number of correct words per minute. |
| Notes |  |

#### Faber and Visscher, 2018

| ***Study characteristics*** | |
| --- | --- |
| Methods | Cluster RCT - class-level assignment |
| Participants | Originally 97 schools recruited and randomly assigned to exp, control or waiting group. 2 exp & 11 control pulled out, 2 from waiting gorup added to exp. A further 10 exp schools pulled out of spelling intervention. Spelling sample- 30 exp schools (619 pupils, 52.2% male, 5.8% disadvantaged) and 39 control (986 pupils, 46% male, 4.4% disadvantaged). |
| Interventions | Schools used Snappet spelling for 6 months, while control schools were business as usual. Pupils work on individual tablets on set assignments, and receive immediate feedback if right or wrong. Pupils complete set curriculum assignments, but the app also calculates their abilities based on responses and sets 'adaptive' assignments based on their performance. Teachers can also review scores and adjust assignments to meet needs. Feedback is basic i.e. right or wrong. Assignments and content align to standard curriculum content |
| Outcomes | Cito standardised spelling test- this is already administered twice yearly (Jan/Feb and June). Student survey & student log files also used to identify attitudes and time spent engaged in Snappet. |
| Notes |  |

#### Faber, Lutyen and Visscher, 2017

| ***Study characteristics*** | |
| --- | --- |
| Methods | Cluster RCT - class-level assignment |
| Participants | 97 primary schools recruited, Grade 3 (age 8 or 9) (40 in experimental group, 50 in control group, 7 on a wait list). 2 experimental & 11 control schools dropped out, so 2 from wait list added to exp group. Initial sample then 40 exp group (822 students- 53% male), 39 control schools (986 students- 47% male). |
| Interventions | Schools used Snappet for 5 months, while control schools were business as usual. Pupils worked don individual tablets on set assignments, and receive immediate feedback if right or wrong. Pupils complete set curriculum assignments, but the app also calculates their abilities based on responses and sets 'adaptive' assignments based on their performance. Teachers can also review scores and adjust assignments to meet needs. |
| Outcomes | For attainment meausre, standardised assessment used- Cito (Dutch Institute for Test Development). Maths test measures 2 domains- arithmetic and geometry, time and money calculations.Pretest January 2015, post-test June 2016. Analysis using multilevel regression model |
| Notes |  |

#### Fabian and Topping, 2019

| ***Study characteristics*** | |
| --- | --- |
| Methods | RCT - individual assignment |
| Participants | 74 pupils, grade 5 and 6 in Primary schools. 35 (20 male) in exp group and 39 (17 male) in control group. |
| Interventions | Pupils used apps Skitch and Pixel touch, to photograph objects and calculate angle sizes of real objects, and Measure Map and Area and Perimeter, to calculate areas. Control group carried out same activities but used paper and pencil to draw objects rather than photograph. SAMR model used to categorise activities- a mix of augmentation and modification. Children were able to go out of the classroom to take pictures and measure objects. |
| Outcomes | Maths test was designed for the study, with items on symmetry, angles, area and perimeter, based on standard text book. To support validity, an experienced maths teacher reviewed the test. |
| Notes |  |

#### Hirotake, 2019

| ***Study characteristics*** | |
| --- | --- |
| Methods | RCT - Cluster randomisation |
| Participants | 1656 Grade 1-4 students, elementary school, although high attrition rate between baseline & follow-up. 20 treatment and 20 control groups. 40 classes, randomised as class groups to either condition. |
| Interventions | Think Think app, which includes mini games to support maths learning. Adaptive learning so that games match the ability of the pupil. |
| Outcomes | National Assessment tests for G3, TIMMS for G4, IQ tests for grades 1 & 2. |
| Notes |  |

#### Levesque, Bardack and Chigeda, 2020

| ***Study characteristics*** | |
| --- | --- |
| Methods | Non-clustered blocked individual random assignment (BIRA) RCT |
| Participants | Two government primary schools purposively sampled (rural and peri-rural (i.e. very rural). Both very low income areas with severe challenges, large classes (100 plus) & very low literacy/maths levels. Children in Standard 2- age 6-10. 674 children considered eligable across the 2 schools. Each school had 4 strata (6-7, 8-10, male, female) and were randmoly assigned to maths or literacy treatment group, or control. |
| Interventions | OneCourse app- delivered via tablet and including 1248 instructional literacy units & 282 maths units. Children took turns to attend a learning centre 1 hour per day, and used either maths or literacy app for 40 mins at each session. |
| Outcomes | Early Grade Readong Assessment & Early Grade Maths Assessment (in local Chichewa language). Baseline & endline assessments carried out by research team, who entered pupil responses on a tablet.Application usage data also recorded for each child.Overall composite score for each domain assessed (use these for SR) as well as various target domains. |
| Notes |  |

#### Messer, Thomas, Holliman and Kucirkova, 2018

| ***Study characteristics*** | |
| --- | --- |
| Methods | RCT (convienence sample randomised at pupil level) |
| Participants | 41 pupils from an infant school, aged 5-6. Convenience sample from 2 classes then randomly assigned to one of 3 groups (programmng on ipads, programming using paper/pencil, maths questions on paper/pencil (control)). |
| Interventions | Bee-bot app for iPad- children had to input instructions to help the bee reach the flower (forward, back, left turn 90 deg, right turn 90 deg.). 18 levels available, with a timer & stars for successful completion. Paper & pencil version- screen shots printed & children had to write/draw the commands. Control group undertook a selection of picture-based maths questions (e.g. count the number of clouds & subtract the number of trees) |
| Outcomes | Maths measured designed for the research by researchers in collaboration with teachers, & based onpre-existing tests (British Ability Scales-3 number skills subtest & Wide Range Achievement Test math computation subtest. Administered pre and post-test. |
| Notes |  |

#### Miller and Robertson, 2011

| ***Study characteristics*** | |
| --- | --- |
| Methods | Cluster RCT - school-level assignment |
| Participants | 634 primary school children across 32 schools, year 6 (aged 10-11). Schools mixed across rural, urban, inner city, and mixed ability & gender. |
| Interventions | Dr Kawashima's Brain Training, Nintendo DS lite. . |
| Outcomes | 100 item 'number challenge' test, devicsed by authors for a previous study but based on calculations within level D scottish maths curriculum. A range of addition, subtraction, multiplication and division, and timed with max time 25 mins. |
| Notes |  |

#### Outhwaite, Faulder, Gulliford and Pitchford,  2019

| ***Study characteristics*** | |
| --- | --- |
| Methods | RCT - individual assignment |
| Participants | School recruitment event held in Nottingham Apple Training Centre, 12 schools volunteered and parents of children age 4-5 were given chance to 'opt in' for their child.  389 children in final sample who undertook pre and post tests and full intervention- 126 group 1, 131 group 2, 132 control. |
| Interventions | onebillion maths app- maths 3-5 & maths 4-6, designed to cover EYFS maths topics e.g. number, shape, space, measures.  Administered in small groups of 10-15, by teachers, and children worked on their own ipads |
| Outcomes | Standardised PTM5 assessment used. Paper based, age appropriate (for 4-5 year olds in summer term). Designed to be used by schools to track progress and covers items from EYFS maths curriculum. Independent of the maths app. Adminsitered one to one by researhcers for each child, |
| Notes |  |

#### Pitchford and Outwaite, 2019

| ***Study characteristics*** | |
| --- | --- |
| Methods | RCT- individual randomisation across gender (meaning equal representation of boys and girls in each group) |
| Participants | Final sample 153 (76 intervention, 77 control). Children aged 6-8 approx |
| Interventions | onebillion app (as above). Maths intervention in local language. |
| Outcomes | A test consisting of 98 items, measuring different aspects of curriculum and conceptual knowledge was used to assess mathematics |
| Notes |  |

#### Pitchford, 2015

| ***Study characteristics*** | |
| --- | --- |
| Methods | RCT- individual randomisation across gender (meaning equal representation of boys and girls in each group) |
| Participants | Final sample 283 (104 intervention, 79 placebo, 100 control). 350 enrolled in study originally, 32 randlmly excluded due to number of ipads available. 318 randmised to one of 3 groups, 304 pre-tested (attrition due to absentee or having transferred school) Children aged 6-8 approx |
| Interventions | Maths app- onebillion, translated into local language. Content designed to cover local curriculum topics. Children worked at their own pace through content, choosing where to focus, and answered a quiz at the end which they had to get 100% correct before they could progress to the next level. |
| Outcomes | 2 x Researcher-developed tests, based on common maths currirulum (reliability and validity tested & presented separately). Designed to test mathematical ability and basic maths skills, these both administered via tablet, while a third test was deivsed to be adminsitered via papre & pen to assess transferability of skills from tablet. |
| Notes |  |

#### Pitchford, Chigeda and Hubber, 2019

| ***Study characteristics*** | |
| --- | --- |
| Methods | RCT - individual assignment |
| Participants | 360 pupils randomly selected from 3 grades across 2 schools. Chidlren aged between 5 & 12 (mean approx 7-8) |
| Interventions | onebillion app. Reading intervention in local language. |
| Outcomes | Standardised assessment, Early Grade Reading Assessment, translated into local language. |
| Notes |  |

#### Schacter and Jo, 2017

| ***Study characteristics*** | |
| --- | --- |
| Methods | Cluster RCT - class-level assignment |
| Participants | Pretest sample consisted of 433 children (259 Math Shelf students and 174 hands-on lesson students). The post-test sample included 378 students (231 Math Shelf and 147 hands-on lesson children). Mean age 4.59 (intervention), 4.49 (control) |
| Interventions | Maths Shelf: maths app for preschool and year 1 pupils (USA therefore primary age equivalent). Children work through various activities at their own speed, and their starting point is matched to their ability (pre-testing carried out). Based on Montessori maths theory and child development. Comparison with usual teaching methods |
| Outcomes | 48 item ipad administered early maths assessment - developed for the study specifically, inlcudding number identificaiton, sequencing, quantities, addition etc. Administered via ipad by school office staff |
| Notes |  |

#### Sutherland, 2019

| ***Study characteristics*** | |
| --- | --- |
| Methods | Cluster RCT - class-level assignmnet |
| Participants | 34 schools, 108 classes, 2564 pupils. Years 4 & 5 |
| Interventions | Explain Everything app: Teachers record their feedback to pupils on their work via an app, then pupils can access this individually via their tablet. Pupils can listen/watch the feedback during class as many times as they want. |
| Outcomes | ACER Essential Learning Metric |
| Notes |  |

#### Yamaç, Ozturk and Mutlu, 2020

| ***Study characteristics*** | |
| --- | --- |
| Methods | Cluster RCT - class-level assignment |
| Participants | Fourth grade primary pupils (ave. age 10.12). 4 classes randomly assigned to 2x exp or 2xcontrol groups. 47 in control group (27F, 2M) & 49 in exp groups (26F, 23M). Middle/higher level income, all familiar with tablets. |
| Interventions | Strategic Digital Writing Environment (SADIWE) developed for this research. Teacher & pupils access via individual tablets, teacher sets writing assignments, SADIWE takes pupils through the stages of writing- planning, idea generation, organsiation etc, and includes video instruction. Pupils work on their own device but can provide feedback to others. At the end, pupils add images & upload their piece to the class blog. Paper and pencil group worked on the same project but in traditional teaching style, with group work face to face. |
| Outcomes | Both groups of students wrote an essay on a topic, and these were scored as follows: writing quality & length of text: scored using '6+1 Writing Assessment Rubric' plus word count. Writing during the sessions (In-progress writing) was also scored this way.   Writing knowledge: pupils answered 3 questions on aspects of writing knowledge (planning, aspects of good writing) & scored based on units of meaning (e.g. I research the idea, plan my essay, show it to a friend for advice = 3 units of meaning) |
| Notes |  |

## Characteristics of excluded studies [ordered by study ID]

| Study | Reason for exclusion |
| --- | --- |
| Ahmed et al, 2020 | Intervention designed for struggling readers and study focused on low-achieving pupils rather than class as a whole. |
| Brezovszky et al, 2019 | Game played on PCs rather than mobile devices |
| Cheung and Xin, 2019 | Intervention is part of a wider literacy programme, inlcuding substantial teacher training, rather than a specific mobile device intervention. |
| Foster et al, 2016 | Intervention undertaken via desk-top computers rather than mobile devices. |
| Hieftje et al, 2017 | Intervention was an after-school activity to support additional learning for a small group, rather than an intervention for the class as a whole. |
| Jones, Christian and Rice, 2016 | Intervention is an overall literacy programme inlcuding tutoring and parent engagement activities, with no specific mobile device component. |
| Klop et al, 2018 | Intervention delivered to small groups rather than to the class as a whole. |
| Neumann, 2018 | Children were younger than the required age gorup (mean age = 45 months) and are in a childcare setting rather than primary school. |
| Nunez et al, 2019 | Intervention included parent instruction and home learning component, rather than a classroom-based intervention. |
| Wolgeuth et al, 2013 | Intervention is an overall literacy package, including teacher training and a range of activities. Delivered in small groups outside of the classroom, with no specific mobile device component. |
| Worth et al, 2015 | Intervention only used mobile devices for part of the study, however a sub-set of data for mobile device element was not available. |
| Worth et al, 2019 | Intervention includes a mix of activities, and spans classroom and home working. |
