# Supplementary material for: A systematic review of mobile device use in the primary school classroom and impact on pupil literacy and numeracy attainment: A systematic review
Source: Campbell Syst Rev. 2024 Jun 20;20(2):e1417. doi: 10.1002/cl2.1417 (PMC11190352; doi:10.1002/cl2.1417)
Supplement: Supplementary file 2 — Supporting information. [file CL2-20-e1417-s001.docx]

# Appendices

## Appendix 1. List of relevant systematic reviews identified

| **Author** | **Title** | **Year** | **Population** | **Type of technology** | **Scope of review** | **Limitations/ justification for further review** |
| --- | --- | --- | --- | --- | --- | --- |
| Haßler, Major and Hennessy, 2015 | Tablet use in schools: a critical review of the evidence for learning outcomes. | 2015 | 5-18 | Tablet | Academic achievement across any learning outcome. Included: literature, maths, social studies, science, economics, SEN | Searches were completed May 2014. Initial scoping of research shows a significant growth in research since then.  Anecdotal evidence suggests that practice has shifted significantly since 2014 in terms of iPad/tablet use in schools, given the first tablet was introduced in 2009 (android, 2010 iPad).  This review considered both primary and post-primary schools, however only considered primary learning outcomes.  Paper highlights the lack of quality research in this area given the emerging area of practice. |
| Abrami, P., Borohkovski, E. & Lysenko, L. (2015) | The effects of ABRACADABRA on reading outcomes: A meta-analysis of applied field research | 2015 | Kindergarten & elementary or equivalent (5-12) | Abracadabra (interactive, web-based software for reading) | Reading outcomes | Meta-analysis on research on a specific intervention (Abracadabra).  This includes wider activities beyond mobile device use. |
| Cheung & Slavin, 2012 | The Effectiveness of education technology for enhancing reading achievement: a Meta-Analysis | 2012 | Grades K-12 or equivalent  (5-18) |  | Impact on reading achievement, including where pupil was previously struggling. | This meta-analysis incorporated support for pupils who are struggling or require additional support, as well as general application in the classroom.  Searches identified studies only up to 2010. Unsurprisingly there was no mention of tablets in education at that stage. |
| Cheung and Slavin, 2013 | The Effectiveness of educational technology applications for enhancing mathematics achievement in K-12 classrooms: A meta-analysis. Best Evidence Encyclopaedia (BEE) | 2013 | Grades K-12 or equivalent  (5-18) | Wider technology | Impact on maths achievement, including where pupil was previously struggling. | This meta-analysis incorporated support for pupils who are struggling or require additional support, as well as general application in the classroom.  Searches identified studies only up to 2011. As above, there was no mention of tablets in education at that stage. |
| Cho et al, 2018 | The Effects of using mobile devices on student achievement in language learning: A Meta-Analysis | 2018 | Primary, post-primary, adult education | Mobile technology | Language learning. | Only considered the impact of technology on language learning.  Limited search terms. Key words used: ‘language learning’ AND achievement, AND ‘mobile’ OR ‘m-learning’  Grey literature search used ProQuest Dissertations & Theses only  Of 20 studies identified, 18 focused on learning English as a second language.  Education of all age groups (primary, post-primary, adult education) included. |
| Harper, 2018 | Technology and teacher-student interactions: A review of empirical research | 2018 | Grades K-12 or equivalent (5-18) | Wider technology | Impact of technology on teacher-student interactions. | Focused specifically on the impact that technology has on interactions of teachers and students in the classroom.  Additionally, the review included only published, peer reviewed studies. |
| Herodotou, 2018 | Young children and tablets: A systematic review of effects on learning and development. | 2017 | 2-5 | Tablets | Learning & development. | Focused only on young children |
| Nguyen et al, 2015 | iPad in higher education- hype and hope | 2014 | Higher education  (18 plus) | iPad (brand-specific) | Wider learning outcomes. | Only peer-reviewed studies included  Older population (higher education)  iPad branded tablets only |
| Tingir et al, 2017 | Effects of mobile devices on K-12 students' achievement: a meta-analysis | 2017 | Grades K-12 or equivalent (5-18) | Mobile technology | Science, maths and reading outcomes. Meta-analyses were conducted across subject areas, device type and school type . | Only Peer-reviewed studies included.  Geographical coverage is limited. While search criteria in relation to school class is not included, the majority of included studies are from countries which use the K-12 system.  Included articles are from the following countries: USA (4), Taiwan (5), India, Turkey, Mexico, New Zealand, Spain (post-primary study). |
| Torgerson et al, 2002 | A systematic review and meta-analysis of the effectiveness of information and communication technology on the teaching of spelling | 2002 | School age (4-18) | ICT in general | Teaching of spelling | Outdated- searches include research up to 2000.  Focused only on the teaching of spelling. |
| Zucker et al, 2009 | The Effects of Electronic Books on Pre-Kindergarten-to-Grade 5 Students' Literacy and Language Outcomes: A Research Synthesis | 2009 | Kindergarten to grade 5 | e-books | Literacy and language | Outdated. technology and practice have shifted significantly since 2009, in particular tablets which were introduced in 2009.  Studied eBooks only, rather than wider technology. |

## Appendix 2. Expert Advisory Group summary paper and Terms of Reference

**Project title: A systematic review of mobile device use in the primary school classroom, and impact on pupil literacy and numeracy attainment.**

**Introduction**

This research is being conducted by Claire Dorris as the thesis component of her Doctorate in Childhood Studies at Queen’s University, Belfast. She is supported by Supervisors Dr. Karen Winter and Dr. Liam O’Hare. A robust systematic review (SR) methodology will be employed; the title has been registered with the Campbell Collaboration and their methodology will be employed.

**The Campbell Collaboration website defines a systematic review as follows:**

*A systematic review aims to sum up the best available research on a specific question by synthesizing the results of several studies. Transparent procedures are used to find, evaluate and synthesize the results of relevant research. Procedures are explicitly defined in advance, to ensure that the exercise is transparent and can be replicated. This practice is also designed to minimize bias. Studies included in a review are screened for quality, so the findings of a large number of studies can be combined.*

**Rationale for the review**

The application of technology in classrooms has expanded rapidly in line with technological advances, from desktop computers to the more recent introduction of tablets and mobile devices. As a relatively new area of pedagogy, it remains an area of uncertainty, particularly with regard to the impact on pupils’ educational experience and learning outcomes. Any innovation in the classroom has the capacity to impact all children and young people to a greater or lesser extent; it is therefore critical that educators are equipped with the skills and knowledge to use emerging technology appropriately and effectively to best support pupil attainment. The proposed review has important policy and practice implications across a number of areas, including:

- Curriculum development and delivery
- Technical provision in schools
- Teacher training and ongoing professional development
- Online safety

A systematic review of existing research is timely and has an important contribution to make in ensuring that future decisions across educational policy, practice and professional development, are informed by evidence of good practice.

**Definition of mobile devices:** For the purposes of this systematic review, mobile devices are defined as handheld computing devices, including tablet computers of varying sizes (iPad and other brands) and smartphones (defined as those with a touchscreen interface which can connect to the internet).

**Age range of interest:** This systematic review will focus on the use of mobile devices in the primary school classroom (and global equivalents). The population of interest will be children aged 4-12.

**Outcomes of interest:** this review aims to identify and synthesise evidence on the impact that mobile devices have on pupil literacy and numeracy attainment.

**Research Question**

Specifically, the review will aim to answer the following question:

1. What is the effect of mobile device integration in the primary school classroom on children's literacy and numeracy attainment outcomes?
2. Are there specific devices which are more effective in supporting literacy and numeracy? (tablets, smartphones or handheld games consoles)
3. Are there specific activities which are more effective in supporting literacy and numeracy?
4. Are there specific groups of children for whom mobile devices are more effective in supporting literacy and numeracy? (Across age group and gender).
5. Do the benefits of mobile devices for learning last for any time beyond the study?
6. What is the quality of available evidence on the use of mobile devices in primary/elementary education, and where is further research needed in this regard?

**Proposed methodology**

This research will take the form of a robust Systematic Review (SR) and meta-analysis of the existing evidence base. Key research tasks include:

- Establishment of an Expert Advisory Group to advise on research questions and methodology, and to support interpretation of findings.
- A robust and systematic search of existing literature, both published and unpublished.
- Appraisal and synthesis of identified literature, including a meta-analysis of identified research studies if appropriate.
- Production of a systematic review and meta-analysis report, conducted to Campbell Collaboration standards, including a Plain Language Summary to ensure the findings of the review are accessible by practitioners and policy makers.

**Expert Advisory Group Terms of Reference**

An Expert Advisory Group is being established to support this systematic review process; this will ensure that the review itself, including scope, research questions and interpretation of findings, reflects everyday practice as far possible.

**Your role**

As an Expert Advisory Group member, your support would be appreciated in the following areas:

- Refining the review methodology to ensure this systematic review reflects the current needs & experiences of primary school practitioners as they embed mobile technology in their classrooms.
- Supporting the identification of relevant research studies.
- Supporting the interpretation of findings of the review in a user-friendly way which can be understood by a wide range of stakeholder audiences for whom the findings may be relevant.
- Highlighting any relevant groups, activities or policy or practice developments which may be of interest.
- Supporting the dissemination of findings to appropriate audiences following completion.

**Engagement activities**

The project is due to be completed in December 2020, and your involvement is requested for the full project period. However, as a voluntary group of Expert Advisors, time commitment and engagement will be kept to a minimum.

- It is envisaged that the advice of the Expert Advisory Group will be sought at three key points:
  - Sept/Oct 2019 (to finalise the review methodology)
  - Jan/Feb 2020 (to review emerging findings)
  - Aug/Sept 2020 (to advise on review drafting)
- Engagement will take place via email where possible, with telephone or face to face meeting only when essential.

## Appendix 3. Expert Advisory Group SAMR task

**Aligning interventions to the SAMR framework – Expert Advisory Group task**

The SAMR Framework (Puentedura, 2006), compares activities undertaken using technology with the everyday activities they are replacing (e.g. reading an e-book rather than a paperback), and asks to what extent the use of technology has added to the learning experience. I would like your help in identifying where you think the digital activities that I’ve identified in the research lie within this framework.

**Each of the activities is described in the table below. Please read the detail on each, and indicate with an X whether you feel it describes:**

- A substitution for usual class activities: it simply replaces one activity with the same activity on a device (e.g. reading a book vs reading the same book on a kindle or e-reader).
- An augmentation of usual class activities: it replaces usual activities with a digital version, and adds some additional functions (e.g. writing an essay with pen/paper vs writing an essay in MS Word, therefore being able to format, add some pictures etc).
- A modification of usual class activities: it allows for activities to be significantly redesigned (e.g. accessing the internet to conduct individual research for an assignment).
- A redefinition of usual class activities: it allows for previously unachievable activities to be undertaken (e.g. a multi-media assignment with video and audio input).

It would also be useful to have an idea of how the activities used in research on this subject actually reflect the ways in which mobile devices are used in everyday teaching. Therefore, it would be great if you could add a comment on whether the activity is similar to the types of activities you (or colleagues) use in teaching.

|  | **SAMR classification (mark one with X)** | | | |  |
| --- | --- | --- | --- | --- | --- |
| **Intervention/activity description** | **Substitution**  (The same as the equivalent non-digital activity) | **Augmentation**  (The same as non-digital activity but some additional functions) | **Modification**  (significantly different from what was possible in non-digital activity) | **Redefinition**  (Allowing completely new activities not possible without technology) | **Does this activity reflect how you might use mobile devices in class?** |
| ‘Brain Challenge’ mobile phone game. 4 maths-related tasks - children receive feedback on their number correct within a set time, and their highest score. They must play each task five times before unlocking the next level, therefore encouraging repetition. |  |  |  |  |  |
| Interactive e-book, read on a tablet. Children name the characters, and at various decision points, choose what they want to happen next, meaning the book can be read several times with different storylines.   Books have an inbuilt dictionary to look up any words they don’t know, and there are comprehension questions (with feedback on answers) before each new chapter, along with discussion points for children to talk about with their classmates. |  |  |  |  |  |
| Standard e-book. A digital version of standard class curriculum books, read on a tablet. No additional functions beyond the text. |  |  |  |  |  |
| ‘Snappet app’ via iPads, for maths and literature. Pupils work on assignments set by the teacher on their individual tablets, and receive immediate feedback via the app if they are right or wrong.   Pupils complete the set curriculum assignments, and the app also sets 'adaptive' additional assignments based on their performance and ability level.   Teachers can review scores and adjust assignments to meet individual needs. |  |  |  |  |  |
| Skitch app (a photo mark-up app) combined with digital measuring tools on iPad to teach maths (angles, shapes, areas, perimeters).   Pupils use the tablet to photograph real objects outdoors or around the school and then calculate angle sizes they see in the picture, mark lines of symmetry and measure areas and perimeters of the shapes they have photographed. |  |  |  |  |  |
| ‘Think Think!’ app- 100 fun mini games designed to stimulate critical thinking.  Designed to be used for 10 minutes per day to keep it fresh & fun & help children stay motivated to use it. The difficulty of the games adapts to match the child’s ability. |  |  |  |  |  |
| ‘onebillion’ app (maths & literacy) - children work through instructional units in maths and/or literacy using iPad.   Children work at their own pace, activities are interactive (e.g. using touchscreen function to manipulate objects) and they get feedback straight away on their progress. |  |  |  |  |  |
| ‘Bee-bot’ app (maths)- played on iPad. Children input basic coding instructions to make an onscreen bee move towards a flower (e.g. forward, left turn 90 degrees etc). Various levels of difficulty, and can be used individually or in groups. |  |  |  |  |  |
| Dr Kawashima’s Brain Training, played on Nintendo DS lite. Series of fun challenges to solve which challenge the brain and aim to stimulate critical thinking. No specific curriculum content. |  |  |  |  |  |
| Maths Shelf app: based on theories of child development and Montessori teaching (using shapes, colours, repetition, clear goals, feedback). Children take a ‘test’ to determine their starting level, then work through activities at their own speed, receive feedback and difficulty adapts to their own ability. Teachers receive weekly performance/usage reports to track their pupils’ progress. |  |  |  |  |  |
| Explain Everything app (effective feedback function) – teachers use the app to record video or audio feedback to individual pupils on their work. Pupils can view feedback as many times as needed, at their own pace. Encourages more detailed feedback from teachers and helps pupils make use of the feedback to develop their work. |  |  |  |  |  |
| Strategic Digital Writing Environment (SADWE) –to support creative writing.   Teacher sets assignment via app, which then takes pupils through the stages in creative writing, from planning, generating ideas, organising their notes, drafting the essay, formatting and adding pictures. App includes instructional videos.   Pupils work individually on their own iPad, but can review and provide feedback to other pupils on their work, and at the end of the process, pupils can upload their work to a class blog and share via (some) social media sites. |  |  |  |  |  |
| General 1:1 iPad programme- all pupils had access to an iPad each, and used them at various stages throughout the day for different game-based activities via apps. |  |  |  |  |  |

## Appendix 4. List of databases searched

| **Searches completed** |
| --- |
| **Journals:** |
| British Journal of Educational Technology https://bera-journals.onlinelibrary.wiley.com/journal/14678535 |
| Computers & Education https://www.sciencedirect.com/journal/computers-and-education |
| **Websites:** |
| EEF https://educationendowmentfoundation.org.uk/ |
| National Literacy Trust https://literacytrust.org.uk/ |
| British Educational Research Association https://www.bera.ac.uk/ |
| The Sutton Trust https://www.suttontrust.com/ |
| **Government websites: (education)** |
| England https://www.gov.uk/government/organisations/department-for-education |
| Northern Ireland https://www.education-ni.gov.uk/ |
| Ireland https://www.gov.ie/en/organisation/department-of-education/ |
| Scotland https://education.gov.scot/ |
| Wales https://www.gov.wales/education-skills |
| **Conference procedings:** |
| International Society for Technology in Education https://iste.org/ |
| BETT https://www.bettshow.com/ |
| British Educational Research Conference https://www.bera.ac.uk/conference/bera-conference-2023 |
| European Conference on Education https://ece.iafor.org/ |
| **Databases** |
| British Education Index (EBSCOhost) |
| Child Development & Adolescent Studies (EBSCOhost) |
| Cochrane Central Register of Controlled Trials |
| Directory of Open Access Journals |
| Education Abstracts (EBSCOhost) |
| ERIC (Education Resources Information Centre) (EBSCOhost) |
| International Bibliography of the Social Sciences (IBSS) (ProQuest) |
| Education Journals (ProQuest) |
| PsychInfo (OVID) |
| Scopus |
| SciELO |
| Social Science Citation Index (Web of Science) |
| **Review databases** |
| Campbell Collaboration |
| Cochrane Database of Systematic Reviews |
| EPPI Centre Database of Education Research |
| **Unpu**blished/grey literature |
| OECD Education iLibrary |
| Current Educational Research in the UK (CERUK)- Centre for the Use of Research & Evidence in Education |
| EducationLine (EBSCOhost) |
| OpenGrey |
| Microsoft Academic Search |
| ProQuest Dissertation and Theses |
| Google Scholar (first 500 hits- 25 pages) |

## Appendix 5. Search terms and keywords

| **Key concepts** | **Intervention (#1)** | **Population: Children aged 4-11 (#2)** | **Outcome area 1 (#3a)** | **Outcome area 2 (#3b)** | **Design (#4)** |
| --- | --- | --- | --- | --- | --- |
| Free text terms or natural language terms | iPad  tablet  tablet computer*  touchscreen  app  application  Handheld device  Portable computer  e-book  ebook  e-reader  ereader  electronic storybook  Game* console  Digital game  Smartphone  smart phone  Mobile phone  iPhone  Cell* phone  Portable cell* phone  Mobile telephone  Cell* telephone  Personal Digital Assistant PDA*  Transportable Cell* Phone  1:1 computing  Online instruction  M-learning | Child*  Pupil  minor  student  AND  Primary school  Elementary school  Junior school  Kindergarten  Age 4, 5, 6…  4 year old, 5 year old, 6 year old…  Grade 1, grade 2, grade 3…  1^st^ grade, 2^nd^ grade, 3^rd^ grade...  First grade, second grade, third grade… | Literacy  Reading  Writing  Handwriting  Transcription  Verbal reasoning  Critical thinking  Comprehension  Notetaking  Composition  Listening skills  Phonics  Phonemic awareness  Alphabet  Spell*  Vocabulary  Punctuation  Grammar  Reading fluency  Speaking skills  Spoken language  Critical literacy  Literacy skills | numeracy  number*  math*  arithmetic  sums  calculat*  addition  adding  subtract*  multiplication  division  count*  algebra  fractions  decimal*  geometr*  statistic*  “problem solving”  “shape sort*”  “mathematical literacy”  “quantitative literacy” | RCT  Randomised Control Trial  Randomised Controlled Trial  Randomized Control Trial  Randomized Controlled Trial  Control Group  Placebo  Random allocation  Random assignment  Single blind  Double blind  Triple blind |
| Subject Terms or headings and/or controlled vocabulary  (From British Education index and ERIC) | Educational technology  Computers in Education  Educational innovations  Internet in Education  Computer Assisted Instruction  Digital Technology  Education-Computer applications  Computer games  Educational games  Handheld devices  Technology integration | Elementary education  Elementary schools  Elementary education research  Primary education  Primary schools  First grade (education) [repeat for all grades]  School children  Elementary school teaching  Primary school teaching  Computers in elementary education | Reading Achievement  Literacy Education  Reading comprehension  Reading achievement  English language education in primary schools  Computers and literacy  Literacy research  Literacy program*  Reading (elementary)  Reading (primary)  Reading enrichment  Reading mobile apps | Mathematics achievement  Numerical calculations  Mathematics instruction  Mathematics education (primary)  Mathematics education (elementary)  Mathematics education (primary)  Mathematics education (preschool) Games in mathematics education  Study and teaching of numeracy  Mathematical ability | Randomised Controlled Trial |

## Appendix 6. Search strings

**Final search strings**

| Database | Population | Setting | Intervention: device | Invervention: subject | Study design | Combined search |
| --- | --- | --- | --- | --- | --- | --- |
| EBSCO | (“Age* 4" OR “age* 5" OR "age* 6" OR “age* 7" OR "age* 8" OR "age* 9" OR "age* 10" OR "age* 11" OR "4 year* old*" OR "5 year* old*" OR "6 year* old*" OR "7 year* old*" OR "8 year* old*" OR "9 year* old*" OR "10 year* old*" OR "11 year* old*" OR Child* OR boy OR girl) AND (school OR class* OR classroom) | "Primary school" OR "Elementary school" OR "Junior school" OR Kindergarten OR "grade 1" OR "grade 2" OR "grade 3" OR "grade 4" OR "grade 5" OR “First Grade“ OR “Second Grade“ OR “Third Grade“ OR “Fourth Grade“ OR “Fifth Grade“ OR “1st grade“ OR “2nd grade“ OR “3rd Grade“ OR “4th Grade“ OR “5th grade“ | iPad OR tablet OR "tablet computer*" OR touchscreen OR app OR "handheld device" OR "handheld computer" OR "PDA" OR "personal digital assistant" OR "portable computer" OR "e-book" OR ebook OR "e-reader" OR ereader OR "electronic storybook" OR "game* console" OR "digital game" OR smartphone OR "smart phone" OR "mobile phone" OR iPhone OR "cell* phone" OR "portable cell* phone" OR "mobile telephone" OR "cell* telephone" OR "transportable Cell* Phone" OR "1:1 comput*" OR "online instruction" OR "mobile learn*" OR "M-learning" | Literacy OR Reading OR Writing OR Handwriting OR Transcription OR "Verbal reasoning" OR "Critical thinking" OR Comprehension OR Notetaking OR Composition OR "Listening skills" OR Phonic* OR "phonemic awareness" OR Alphabet OR Spell* OR Vocabulary OR Punctuation OR Grammar OR "Reading fluency" OR "Speaking skills" OR "Spoken language" OR "Critical literacy" OR "literacy skills" OR numeracy OR number* OR math* OR arithmetic OR sums OR calculat* OR addition OR adding OR subtract* OR multiplication OR division OR count* OR algebra OR fractions OR decimal* OR geometr* OR statistic* OR “problem solving” OR “shape sort*” OR “mathematical literacy” OR “quantitative literacy” | RCT OR "randomised control trial" OR "randomised controlled trial" OR "randomized control trial" OR "randomized controlled trial" OR randomised OR randomized OR placebo* OR (random* AND (allocat* OR assign*)) OR (blind* AND (single OR double OR treble OR triple)) | (ab(("Age* 4" OR "age* 5" OR "age* 6" OR "age* 7" OR "age* 8" OR "age* 9" OR "age* 10" OR "age* 11" OR "4 year* old*" OR "5 year* old*" OR "6 year* old*" OR "7 year* old*" OR "8 year* old*" OR "9 year* old*" OR "10 year* old*" OR "11 year* old*" OR Child* OR boy OR girl) AND (school OR class* OR classroom)) OR ab("Primary school" OR "Elementary school" OR "Junior school" OR Kindergarten OR "grade 1" OR "grade 2" OR "grade 3" OR "grade 4" OR "grade 5" OR "First Grade" OR "Second Grade" OR "Third Grade" OR "Fourth Grade" OR "Fifth Grade" OR "1st grade" OR "2nd grade" OR "3rd Grade" OR "4th Grade" OR "5th grade")) AND (ab(Literacy OR Reading OR Writing OR Handwriting OR Transcription OR "Verbal reasoning" OR "Critical thinking" OR Comprehension OR Notetaking OR Composition OR "Listening skills" OR Phonic* OR "phonemic awareness" OR Alphabet OR Spell* OR Vocabulary OR Punctuation OR Grammar OR "Reading fluency" OR "Speaking skills" OR "Spoken language" OR "Critical literacy" OR "literacy skills" OR numeracy OR number* OR math* OR arithmetic OR sums OR calculat* OR addition OR adding OR subtract* OR multiplication OR division OR count* OR algebra OR fractions OR decimal* OR geometr* OR statistic* OR "problem solving" OR "shape sort*" OR "mathematical literacy" OR "quantitative literacy") AND ab(iPad OR tablet OR "tablet computer*" OR touchscreen OR app OR "handheld device" OR "handheld computer" OR "PDA" OR "personal digital assistant" OR "portable computer" OR "e-book" OR ebook OR "e-reader" OR ereader OR "electronic storybook" OR "game* console" OR "digital game" OR smartphone OR "smart phone" OR "mobile phone" OR iPhone OR "cell* phone" OR "portable cell* phone" OR "mobile telephone" OR "cell* telephone" OR "transportable Cell* Phone" OR "1:1 comput*" OR "online instruction" OR "mobile learn*" OR "M-learning")) AND ab(RCT OR "randomised control trial" OR "randomised controlled trial" OR "randomized control trial" OR "randomized controlled trial" OR randomised OR randomized OR placebo* OR (random* AND (allocat* OR assign*)) OR (blind* AND (single OR double OR treble OR triple))) |
| Proquest | (“Age* 4" OR “age* 5" OR "age* 6" OR “age* 7" or "age* 8" OR "age* 9" OR "age* 10" OR "age* 11" OR "4 year* old*" OR "5 year* old*" OR "6 year* old*" OR "7 year* old*" OR "8 year* old*" OR "9 year* old*" OR "10 year* old*" OR "11 year* old*" OR Child* OR boy OR girl) AND (school OR class* OR classroom) | "Primary school" OR "Elementary school" OR "Junior school" OR Kindergarten OR "grade 1" OR "grade 2" OR "grade 3" OR "grade 4" OR "grade 5" OR “First Grade“ OR “Second Grade“ OR “Third Grade“ OR “Fourth Grade“ OR “Fifth Grade“ OR “1st grade“ OR “2nd grade“ OR “3rd Grade“ OR “4th Grade“ OR “5th grade“ | iPad OR tablet OR "tablet computer*" OR touchscreen OR app OR "handheld device" OR "handheld computer" OR "PDA" OR "personal digital assistant" OR "portable computer" OR "e-book" OR ebook OR "e-reader" OR ereader OR "electronic storybook" OR "game* console" OR "digital game" OR smartphone OR "smart phone" OR "mobile phone" OR iPhone OR "cell* phone" OR "portable cell* phone" OR "mobile telephone" OR "cell* telephone" OR "transportable Cell* Phone" OR "1:1 comput*" OR "online instruction" OR "mobile learn*" OR "M-learning" | Literacy OR Reading OR Writing OR Handwriting OR Transcription OR "Verbal reasoning" OR "Critical thinking" OR Comprehension OR Notetaking OR Composition OR "Listening skills" OR Phonic* OR "phonemic awareness" OR Alphabet OR Spell* OR Vocabulary OR Punctuation OR Grammar OR "Reading fluency" OR "Speaking skills" OR "Spoken language" OR "Critical literacy" OR "literacy skills" OR numeracy OR number* OR math* OR arithmetic OR sums OR calculat* OR addition OR adding OR subtract* OR multiplication OR division OR count* OR algebra OR fractions OR decimal* OR geometr* OR statistic* OR “problem solving” OR “shape sort*” OR “mathematical literacy” OR “quantitative literacy” | RCT OR "randomised control trial" OR "randomised controlled trial" OR "randomized control trial" OR "randomized controlled trial" OR randomised OR randomized OR placebo* OR (random* AND (allocat* OR assign*)) OR (blind* AND (single OR double OR treble OR triple)) | (ab(("Age* 4" OR "age* 5" OR "age* 6" OR "age* 7" OR "age* 8" OR "age* 9" OR "age* 10" OR "age* 11" OR "4 year* old*" OR "5 year* old*" OR "6 year* old*" OR "7 year* old*" OR "8 year* old*" OR "9 year* old*" OR "10 year* old*" OR "11 year* old*" OR Child* OR boy OR girl) AND (school OR class* OR classroom)) OR ab("Primary school" OR "Elementary school" OR "Junior school" OR Kindergarten OR "grade 1" OR "grade 2" OR "grade 3" OR "grade 4" OR "grade 5" OR "First Grade" OR "Second Grade" OR "Third Grade" OR "Fourth Grade" OR "Fifth Grade" OR "1st grade" OR "2nd grade" OR "3rd Grade" OR "4th Grade" OR "5th grade")) AND (ab(Literacy OR Reading OR Writing OR Handwriting OR Transcription OR "Verbal reasoning" OR "Critical thinking" OR Comprehension OR Notetaking OR Composition OR "Listening skills" OR Phonic* OR "phonemic awareness" OR Alphabet OR Spell* OR Vocabulary OR Punctuation OR Grammar OR "Reading fluency" OR "Speaking skills" OR "Spoken language" OR "Critical literacy" OR "literacy skills" OR numeracy OR number* OR math* OR arithmetic OR sums OR calculat* OR addition OR adding OR subtract* OR multiplication OR division OR count* OR algebra OR fractions OR decimal* OR geometr* OR statistic* OR "problem solving" OR "shape sort*" OR "mathematical literacy" OR "quantitative literacy") AND ab(iPad OR tablet OR "tablet computer*" OR touchscreen OR app OR "handheld device" OR "handheld computer" OR "PDA" OR "personal digital assistant" OR "portable computer" OR "e-book" OR ebook OR "e-reader" OR ereader OR "electronic storybook" OR "game* console" OR "digital game" OR smartphone OR "smart phone" OR "mobile phone" OR iPhone OR "cell* phone" OR "portable cell* phone" OR "mobile telephone" OR "cell* telephone" OR "transportable Cell* Phone" OR "1:1 comput*" OR "online instruction" OR "mobile learn*" OR "M-learning")) AND ab(RCT OR "randomised control trial" OR "randomised controlled trial" OR "randomized control trial" OR "randomized controlled trial" OR randomised OR randomized OR placebo* OR (random* AND (allocat* OR assign*)) OR (blind* AND (single OR double OR treble OR triple))) |
| OVID | (Age$ 4 OR age$ 5 OR age$ 6 OR age$ 7 or age$ 8 OR age$ 9 OR age$ 10 OR age$ 11 OR 4 year$ old$ OR 5 year$ old$ OR 6 year$ old$ OR 7 year$ old$ OR 8 year$ old$ OR 9 year$ old$ OR 10 year$ old$ OR 11 year$ old$ OR Child$ OR boy OR girl) AND (school OR class$ OR classroom) | Primary school OR Elementary school OR Junior school OR Kindergarten OR grade 1 OR grade 2 OR grade 3 OR grade 4 OR grade 5 OR First Grade OR Second Grade OR Third Grade OR Fourth Grade OR Fifth Grade OR 1st grade OR 2nd grade OR 3rd Grade OR 4th Grade OR 5th grade | iPad OR tablet OR tablet computer$ OR touchscreen OR app OR handheld device OR handheld computer OR PDA OR personal digital assistant OR portable computer OR e-book OR ebook OR e-reader OR ereader OR electronic storybook OR game$ console OR digital game OR smartphone OR smart phone OR mobile phone OR iPhone OR cell$ phone OR portable cell$ phone OR mobile telephone OR cell$ telephone OR transportable Cell$ Phone OR 1:1 comput$ OR online instruction OR mobile learn$ OR M-learning | Literacy OR Reading OR Writing OR Handwriting OR Transcription OR Verbal reasoning OR Critical thinking OR Comprehension OR Notetaking OR Composition OR Listening skills OR Phonic$ OR phonemic awareness OR Alphabet OR Spell$ OR Vocabulary OR Punctuation OR Grammar OR Reading fluency OR Speaking skills OR Spoken language OR Critical literacy OR literacy skills OR numeracy OR number$ OR math$ OR arithmetic OR sums OR calculat$ OR addition OR adding OR subtract$ OR multiplication OR division OR count$ OR algebra OR fractions OR decimal$ OR geomet$ OR statistic$ OR problem solving OR shape sort$ OR mathematical literacy OR quantitative literacy | RCT OR randomised control trial OR randomised controlled trial OR randomized control trial OR randomized controlled trial OR randomised OR randomized OR placebo$ OR (random$ AND (allocat$ OR assign$)) OR (blind$ AND (single OR double OR treble OR triple)) |  |
| Scopus | ABS(“Age* 4" OR “age* 5" OR "age* 6" OR “age* 7" OR "age* 8" OR "age* 9" OR "age* 10" OR "age* 11" OR "4 year* old*" OR "5 year* old*" OR "6 year* old*" OR "7 year* old*" OR "8 year* old*" OR "9 year* old*" OR "10 year* old*" OR "11 year* old*" OR Child* OR boy OR girl) AND ABS(school OR class* OR classroom) | ABS("Primary school" OR "Elementary school" OR "Junior school" OR Kindergarten OR "grade 1" OR "grade 2" OR "grade 3" OR "grade 4" OR "grade 5" OR “First Grade“ OR “Second Grade“ OR “Third Grade“ OR “Fourth Grade“ OR “Fifth Grade“ OR “1st grade“ OR “2nd grade“ OR “3rd Grade“ OR “4th Grade“ OR “5th grade“) | ABS(iPad OR tablet OR "tablet computer*" OR touchscreen OR app OR "handheld device" OR "handheld computer" OR "PDA" OR "personal digital assistant" OR "portable computer" OR "e-book" OR ebook OR "e-reader" OR ereader OR "electronic storybook" OR "game* console" OR "digital game" OR smartphone OR "smart phone" OR "mobile phone" OR iPhone OR "cell* phone" OR "portable cell* phone" OR "mobile telephone" OR "cell* telephone" OR "transportable Cell* Phone" OR "1:1 comput*" OR "online instruction" OR "mobile learn*" OR "M-learning") | ABS(Literacy OR Reading OR Writing OR Handwriting OR Transcription OR "Verbal reasoning" OR "Critical thinking" OR Comprehension OR Notetaking OR Composition OR "Listening skills" OR Phonic* OR "phonemic awareness" OR Alphabet OR Spell* OR Vocabulary OR Punctuation OR Grammar OR "Reading fluency" OR "Speaking skills" OR "Spoken language" OR "Critical literacy" OR "literacy skills" OR numeracy OR number* OR math* OR arithmetic OR sums OR calculat* OR addition OR adding OR subtract* OR multiplication OR division OR count* OR algebra OR fractions OR decimal* OR geometr* OR statistic* OR “problem solving” OR “shape sort*” OR “mathematical literacy” OR “quantitative literacy”) | ABS(RCT OR "randomised control trial" OR "randomised controlled trial" OR "randomized control trial" OR "randomized controlled trial" OR randomised OR randomized OR placebo* OR (random* AND (allocat* OR assign*)) OR (blind* AND (single OR double OR treble OR triple))) | (ABS(iPad OR tablet OR "tablet computer*" OR touchscreen OR app OR "handheld device" OR "handheld computer" OR "PDA" OR "personal digital assistant" OR "portable computer" OR "e-book" OR ebook OR "e-reader" OR ereader OR "electronic storybook" OR "game* console" OR "digital game" OR smartphone OR "smart phone" OR "mobile phone" OR iPhone OR "cell* phone" OR "portable cell* phone" OR "mobile telephone" OR "cell* telephone" OR "transportable Cell* Phone" OR "1:1 comput*" OR "online instruction" OR "mobile learn*" OR "M-learning")) AND (ABS(RCT OR "randomised control trial" OR "randomised controlled trial" OR "randomized control trial" OR "randomized controlled trial" OR randomised OR randomized OR placebo* OR (random* AND (allocat* OR assign*)) OR (blind* AND (single OR double OR treble OR triple)))) AND (ABS(Literacy OR Reading OR Writing OR Handwriting OR Transcription OR "Verbal reasoning" OR "Critical thinking" OR Comprehension OR Notetaking OR Composition OR "Listening skills" OR Phonic* OR "phonemic awareness" OR Alphabet OR Spell* OR Vocabulary OR Punctuation OR Grammar OR "Reading fluency" OR "Speaking skills" OR "Spoken language" OR "Critical literacy" OR "literacy skills" OR numeracy OR number* OR math* OR arithmetic OR sums OR calculat* OR addition OR adding OR subtract* OR multiplication OR division OR count* OR algebra OR fractions OR decimal* OR geometr* OR statistic* OR "problem solving" OR "shape sort*" OR "mathematical literacy" OR "quantitative literacy")) AND ((ABS("Age* 4" OR "age* 5" OR "age* 6" OR "age* 7" OR "age* 8" OR "age* 9" OR "age* 10" OR "age* 11" OR "4 year* old*" OR "5 year* old*" OR "6 year* old*" OR "7 year* old*" OR "8 year* old*" OR "9 year* old*" OR "10 year* old*" OR "11 year* old*" OR Child* OR boy OR girl) AND ABS(school OR class OR classroom)) OR (ABS("Primary school" OR "Elementary school" OR "Junior school" OR Kindergarten OR "grade 1" OR "grade 2" OR "grade 3" OR "grade 4" OR "grade 5" OR "First Grade" OR "Second Grade" OR "Third Grade" OR "Fourth Grade" OR "Fifth Grade" OR "1st grade" OR "2nd grade" OR "3rd Grade" OR "4th Grade" OR "5th grade"))) AND SUBJAREA(ARTS OR BUSI OR DECI OR ECON OR PSYC OR SOCI) |
| Web of Science | (“Age* 4" OR “age* 5" OR "age* 6" OR “age* 7" OR "age* 8" OR "age* 9" OR "age* 10" OR "age* 11" OR "4 year* old*" OR "5 year* old*" OR "6 year* old*" OR "7 year* old*" OR "8 year* old*" OR "9 year* old*" OR "10 year* old*" OR "11 year* old*" OR Child* OR boy OR girl) AND (school OR class* OR classroom) | "Primary school" OR "Elementary school" OR "Junior school" OR Kindergarten OR "grade 1" OR "grade 2" OR "grade 3" OR "grade 4" OR "grade 5" OR “First Grade“ OR “Second Grade“ OR “Third Grade“ OR “Fourth Grade“ OR “Fifth Grade“ OR “1st grade“ OR “2nd grade“ OR “3rd Grade“ OR “4th Grade“ OR “5th grade“ | iPad OR tablet OR "tablet computer*" OR touchscreen OR app OR "handheld device" OR "handheld computer" OR "PDA" OR "personal digital assistant" OR "portable computer" OR "e-book" OR ebook OR "e-reader" OR ereader OR "electronic storybook" OR "game* console" OR "digital game" OR smartphone OR "smart phone" OR "mobile phone" OR iPhone OR "cell* phone" OR "portable cell* phone" OR "mobile telephone" OR "cell* telephone" OR "transportable Cell* Phone" OR "1:1 comput*" OR "online instruction" OR "mobile learn*" OR "M-learning" | Literacy OR Reading OR Writing OR Handwriting OR Transcription OR "Verbal reasoning" OR "Critical thinking" OR Comprehension OR Notetaking OR Composition OR "Listening skills" OR Phonic* OR "phonemic awareness" OR Alphabet OR Spell* OR Vocabulary OR Punctuation OR Grammar OR "Reading fluency" OR "Speaking skills" OR "Spoken language" OR "Critical literacy" OR "literacy skills" OR numeracy OR number* OR math* OR arithmetic OR sums OR calculat* OR addition OR adding OR subtract* OR multiplication OR division OR count* OR algebra OR fractions OR decimal* OR geometr* OR statistic* OR “problem solving” OR “shape sort*” OR “mathematical literacy” OR “quantitative literacy” | RCT OR "randomised control trial" OR "randomised controlled trial" OR "randomized control trial" OR "randomized controlled trial" OR randomised OR randomized OR placebo* OR (random* AND (allocat* OR assign*)) OR (blind* AND (single OR double OR treble OR triple)) |  |
| Google Scholar |  |  |  |  | (“primary school” OR “elementary school” OR child OR pupil) AND (“mobile learning” OR technology OR tablet OR iPad OR “mobile phone” OR “smart phone”) AND (RCT OR randomised control trial) AND (reading OR writing OR maths OR literacy OR numeracy) |  |
| EPPI-Centre Database of Education Research |  |  |  |  | keyword search:  curriculum literacy OR maths Educational setting: primary school topic of study: teaching & learning Freetext: technology |  |
| Campbell Collaboration |  |  |  |  | keyword search: maths OR numeracy OR reading OR writing OR literacy OR technology |  |
| Cochrane Database of Trials, Cochrane Database of Systematic reviews |  | school OR class OR pupil OR student OR "primary school" OR "elementary school" (title, keyword or abstract) | iPad OR "tablet computer*" OR touchscreen OR app OR "handheld device" OR "handheld computer" OR "PDA" OR "personal digital assistant" OR "portable computer" OR "e-book" OR ebook OR "e-reader" OR ereader OR "electronic storybook" OR "game* console" OR "digital game" OR smartphone OR "smart phone" OR "mobile phone" OR iPhone OR "cell* phone" OR "portable cell* phone" OR "mobile telephone" OR "cell* telephone" OR "transportable Cell* Phone" OR "1:1 comput*" OR "online instruction" OR "mobile learn*" OR "M-learning" (title, keyword or abstract) | Literacy OR Reading OR Writing OR Handwriting OR Transcription OR "Verbal reasoning" OR "Critical thinking" OR Comprehension OR Notetaking OR Composition OR "Listening skills" OR Phonic* OR "phonemic awareness" OR Alphabet OR Spell* OR Vocabulary OR Punctuation OR Grammar OR "Reading fluency" OR "Speaking skills" OR "Spoken language" OR numeracy OR number* OR math* OR arithmetic OR sums OR calculat* OR addition OR adding OR subtract* OR multiplication OR division OR algebra OR fractions OR decimal* OR geometr* OR statistic* OR “problem solving” OR “shape sort*” (title) | RCT OR "randomised control trial" OR "randomised controlled trial" (title, keyword or abstract) | Literacy OR Reading OR Writing OR Handwriting OR Transcription OR "Verbal reasoning" OR "Critical thinking" OR Comprehension OR Notetaking OR Composition OR "Listening skills" OR Phonic* OR "phonemic awareness" OR Alphabet OR Spell* OR Vocabulary OR Punctuation OR Grammar OR "Reading fluency" OR "Speaking skills" OR "Spoken language" OR numeracy OR number* OR math* OR arithmetic OR sums OR calculat* OR addition OR adding OR subtract* OR multiplication OR division OR algebra OR fractions OR decimal* OR geometr* OR statistic* OR “problem solving” OR “shape sort*” in Record Title AND school OR class OR pupil OR student OR "primary school" OR "elementary school" in Title Abstract Keyword AND iPad OR "tablet computer*" OR touchscreen OR app OR "handheld device" OR "handheld computer" OR "PDA" OR "personal digital assistant" OR "portable computer" OR "e-book" OR ebook OR "e-reader" OR ereader OR "electronic storybook" OR "game* console" OR "digital game" OR smartphone OR "smart phone" OR "mobile phone" OR iPhone OR "cell* phone" OR "portable cell* phone" OR "mobile telephone" OR "cell* telephone" OR "transportable Cell* Phone" OR "1:1 comput*" OR "online instruction" OR "mobile learn*" OR "M-learning" in Title Abstract Keyword AND RCT OR "randomised control trial" OR "randomised controlled trial" in Title Abstract Keyword - |
| Opengrey |  |  |  |  |  | (Literacy OR Reading OR Writing OR Handwriting OR Transcription OR "Verbal reasoning" OR "Critical thinking" OR Comprehension OR Notetaking OR Composition OR "Listening skills" OR Phonic* OR "phonemic awareness" OR Alphabet OR Spell* OR Vocabulary OR Punctuation OR Grammar OR "Reading fluency" OR "Speaking skills" OR "Spoken language" OR "Critical literacy" OR "literacy skills" OR numeracy OR number*) AND (iPad OR tablet OR "tablet computer*" OR touchscreen OR app OR "handheld device" OR "handheld computer" OR "PDA" OR "personal digital assistant" OR "portable computer" OR "e-book" OR ebook OR "e-reader" OR ereader OR "electronic storybook" OR "game* console" OR "digital game" OR smartphone OR "smart phone" OR "mobile phone" OR iPhone OR "cell* phone" OR "portable cell* phone" OR "mobile telephone" OR "cell* telephone" OR "transportable Cell* Phone" OR "1:1 comput*" OR "online instruction" OR "mobile learn*" OR "M-learning") AND (school OR class OR classroom OR "primary school" OR "elementary school" OR pupil OR child OR "Junior school" OR Kindergarten OR "grade 1" OR "grade 2" OR "grade 3" OR "grade 4" OR "grade 5" OR “First Grade“ OR “Second Grade“ OR “Third Grade“ OR “Fourth Grade“ OR “Fifth Grade“ OR “1st grade“ OR “2nd grade“ OR “3rd Grade“ OR “4th Grade“ OR “5th grade“) AND (RCT OR "randomised control trial" OR "randomised controlled trial" OR "randomized control trial" OR "randomized controlled trial" OR randomised OR randomized OR placebo* OR (random* AND (allocat* OR assign*)) OR (blind* AND (single OR double OR treble OR triple))) NOT "emotional literacy" |
| European conference on Education Research; British Educational research Conference; | Searches run individually: smartphone iPad mobile technology RCT tablet |  |  |  |  |  |
| DOAJ | (searches in title & narrowed by 'education') tablet primary school maths maths AND technology math AND digital smart phone mobile phone digital game reading AND technology e-book digital AND reading RCT AND literacy (abstract) RCT AND maths RCT AND reading |  |  |  |  |  |
| British Educational Research Journal | (“Age* 4" OR “age* 5" OR "age* 6" OR “age* 7" OR "age* 8" OR "age* 9" OR "age* 10" OR "age* 11" OR "4 year* old*" OR "5 year* old*" OR "6 year* old*" OR "7 year* old*" OR "8 year* old*" OR "9 year* old*" OR "10 year* old*" OR "11 year* old*" OR Child* OR boy OR girl) AND (school OR class* OR classroom) | "Primary school" OR "Elementary school" OR "Junior school" OR Kindergarten OR "grade 1" OR "grade 2" OR "grade 3" OR "grade 4" OR "grade 5" OR “First Grade“ OR “Second Grade“ OR “Third Grade“ OR “Fourth Grade“ OR “Fifth Grade“ OR “1st grade“ OR “2nd grade“ OR “3rd Grade“ OR “4th Grade“ OR “5th grade“ | iPad OR tablet OR "tablet computer*" OR touchscreen OR app OR "handheld device" OR "handheld computer" OR "PDA" OR "personal digital assistant" OR "portable computer" OR "e-book" OR ebook OR "e-reader" OR ereader OR "electronic storybook" OR "game* console" OR "digital game" OR smartphone OR "smart phone" OR "mobile phone" OR iPhone OR "cell* phone" OR "portable cell* phone" OR "mobile telephone" OR "cell* telephone" OR "transportable Cell* Phone" OR "1:1 comput*" OR "online instruction" OR "mobile learn*" OR "M-learning" | Literacy OR Reading OR Writing OR Handwriting OR Transcription OR "Verbal reasoning" OR "Critical thinking" OR Comprehension OR Notetaking OR Composition OR "Listening skills" OR Phonic* OR "phonemic awareness" OR Alphabet OR Spell* OR Vocabulary OR Punctuation OR Grammar OR "Reading fluency" OR "Speaking skills" OR "Spoken language" OR "Critical literacy" OR "literacy skills" OR numeracy OR math* OR arithmetic OR sums OR calculat* OR addition OR adding OR subtract* OR multiplication OR division OR count OR algebra OR fractions OR decimal* OR geometr* OR statistic* OR “problem solving” OR “shape sort*” OR “mathematical literacy” OR “quantitative literacy” | RCT OR "randomised control trial" OR "randomised controlled trial" OR "randomized control trial" OR "randomized controlled trial" OR randomised OR randomized OR placebo* OR (random* AND (allocat* OR assign*)) OR (blind* AND (single OR double OR treble OR triple)) |  |
| European Educational Research Journal | ((“Age* 4" OR “age* 5" OR "age* 6" OR “age* 7" OR "age* 8" OR "age* 9" OR "age* 10" OR "age* 11" OR "4 year* old*" OR "5 year* old*" OR "6 year* old*" OR "7 year* old*" OR "8 year* old*" OR "9 year* old*" OR "10 year* old*" OR "11 year* old*" OR Child* OR boy OR girl) AND (school OR class* OR classroom)) OR ("Primary school" OR "Elementary school" OR "Junior school" OR Kindergarten OR "grade 1" OR "grade 2" OR "grade 3" OR "grade 4" OR "grade 5" OR “First Grade“ OR “Second Grade“ OR “Third Grade“ OR “Fourth Grade“ OR “Fifth Grade“ OR “1st grade“ OR “2nd grade“ OR “3rd Grade“ OR “4th Grade“ OR “5th grade“) | "Primary school" OR "Elementary school" OR "Junior school" OR Kindergarten OR "grade 1" OR "grade 2" OR "grade 3" OR "grade 4" OR "grade 5" OR “First Grade“ OR “Second Grade“ OR “Third Grade“ OR “Fourth Grade“ OR “Fifth Grade“ OR “1st grade“ OR “2nd grade“ OR “3rd Grade“ OR “4th Grade“ OR “5th grade“ | iPad OR tablet OR "tablet computer*" OR touchscreen OR app OR "handheld device" OR "handheld computer" OR "PDA" OR "personal digital assistant" OR "portable computer" OR "e-book" OR ebook OR "e-reader" OR ereader OR "electronic storybook" OR "game* console" OR "digital game" OR smartphone OR "smart phone" OR "mobile phone" OR iPhone OR "cell* phone" OR "portable cell* phone" OR "mobile telephone" OR "cell* telephone" OR "transportable Cell* Phone" OR "1:1 comput*" OR "online instruction" OR "mobile learn*" OR "M-learning" | Literacy OR Reading OR Writing OR Handwriting OR Transcription OR "Verbal reasoning" OR "Critical thinking" OR Comprehension OR Notetaking OR Composition OR "Listening skills" OR Phonic* OR "phonemic awareness" OR Alphabet OR Spell* OR Vocabulary OR Punctuation OR Grammar OR "Reading fluency" OR "Speaking skills" OR "Spoken language" OR "Critical literacy" OR "literacy skills" OR numeracy OR number* OR math* OR arithmetic OR sums OR calculat* OR addition OR adding OR subtract* OR multiplication OR division OR count* OR algebra OR fractions OR decimal* OR geometr* OR statistic* OR “problem solving” OR “shape sort*” OR “mathematical literacy” OR “quantitative literacy” | RCT OR "randomised control trial" OR "randomised controlled trial" OR "randomized control trial" OR "randomized controlled trial" OR randomised OR randomized OR placebo* OR (random* AND (allocat* OR assign*)) OR (blind* AND (single OR double OR treble OR triple)) | ((“Age* 4" OR “age* 5" OR "age* 6" OR “age* 7" OR "age* 8" OR "age* 9" OR "age* 10" OR "age* 11" OR "4 year* old*" OR "5 year* old*" OR "6 year* old*" OR "7 year* old*" OR "8 year* old*" OR "9 year* old*" OR "10 year* old*" OR "11 year* old*" OR Child* OR boy OR girl) AND (school OR class* OR classroom)) OR ("Primary school" OR "Elementary school" OR "Junior school" OR Kindergarten OR "grade 1" OR "grade 2" OR "grade 3" OR "grade 4" OR "grade 5" OR “First Grade“ OR “Second Grade“ OR “Third Grade“ OR “Fourth Grade“ OR “Fifth Grade“ OR “1st grade“ OR “2nd grade“ OR “3rd Grade“ OR “4th Grade“ OR “5th grade“)) AND (iPad OR tablet OR "tablet computer*" OR touchscreen OR app OR "handheld device" OR "handheld computer" OR "PDA" OR "personal digital assistant" OR "portable computer" OR "e-book" OR ebook OR "e-reader" OR ereader OR "electronic storybook" OR "game* console" OR "digital game" OR smartphone OR "smart phone" OR "mobile phone" OR iPhone OR "cell* phone" OR "portable cell* phone" OR "mobile telephone" OR "cell* telephone" OR "transportable Cell* Phone" OR "1:1 comput*" OR "online instruction" OR "mobile learn*" OR "M-learning") AND (Literacy OR Reading OR Writing OR Handwriting OR Transcription OR "Verbal reasoning" OR "Critical thinking" OR Comprehension OR Notetaking OR Composition OR "Listening skills" OR Phonic* OR "phonemic awareness" OR Alphabet OR Spell* OR Vocabulary OR Punctuation OR Grammar OR "Reading fluency" OR "Speaking skills" OR "Spoken language" OR "Critical literacy" OR "literacy skills" OR numeracy OR number* OR math* OR arithmetic OR sums OR calculat* OR addition OR adding OR subtract* OR multiplication OR division OR count* OR algebra OR fractions OR decimal* OR geometr* OR statistic* OR “problem solving” OR “shape sort*” OR “mathematical literacy” OR “quantitative literacy”) AND (RCT OR "randomised control trial" OR "randomised controlled trial" OR "randomized control trial" OR "randomized controlled trial" OR randomised OR randomized OR placebo* OR (random* AND (allocat* OR assign*)) OR (blind* AND (single OR double OR treble OR triple))) |

Strings were compiled using the equation **1 AND 2 AND (3a OR 3b) AND 4.**

**#1** ((“Age* 4" OR “age* 5" OR "age* 6" OR “age* 7" or "age* 8" OR "age* 9" OR "age* 10" OR "age* 11" OR "4 year* old*" OR "5 year* old*" OR "6 year* old*" OR "7 year* old*" OR "8 year* old*" OR "9 year* old*" OR "10 year* old*" OR "11 year* old*" OR Child* OR boy OR girl) AND (school OR class* OR classroom)) OR ("Primary school" OR "Elementary school" OR "Junior school" OR Kindergarten OR "grade 1" OR "grade 2" OR "grade 3" OR "grade 4" OR "grade 5" OR “First Grade“ OR “Second Grade“ OR “Third Grade“ OR “Fourth Grade“ OR “Fifth Grade“ OR “1st grade“ OR “2^nd^ grade“ OR “3rd Grade“ OR “4^th^ Grade“ OR “5th grade“)

**#2** iPad OR tablet OR "tablet computer*" OR touchscreen OR app OR "handheld device" OR "handheld computer" OR "PDA" OR "personal digital assistant" OR "portable computer" OR '"e-book" OR ebook OR "e-reader" OR ereader OR "electronic storybook" OR "game* console" OR "digital game" OR smartphone OR "smart phone" OR "mobile phone" OR iPhone OR "cell* phone" OR "portable cell* phone" OR "mobile telephone" OR "cell* telephone" OR "transportable Cell* Phone" OR "1:1 comput*" OR "online instruction" OR "mobile learn*" OR M-learning

**#3a OR #3b** (Literacy OR Reading OR Writing OR Handwriting OR Transcription OR "Verbal reasoning" OR "Critical thinking" OR Comprehension OR Notetaking OR Composition OR "Listening skills" OR Phonic* OR "phonemic awareness" OR Alphabet OR Spell* OR Vocabulary OR Punctuation OR Grammar OR "Reading fluency" OR "Speaking skills" OR "Spoken language" OR "Critical literacy" OR "literacy skills") OR (numeracy OR number* OR math* OR arithmetic OR sums OR calculat* OR addition OR adding OR subtract* OR multiplication OR division OR count* OR algebra OR fractions OR decimal* OR geometr* OR statistic* OR “problem solving” OR “shape sort*” OR “mathematical literacy” OR “quantitative literacy”)

**#4** RCT OR "randomised control trial" OR "randomised controlled trial" OR "randomized control trial" OR "randomized controlled trial" OR randomised OR randomized OR placebo* OR (random* AND (allocat* OR assign*) OR (blind* AND (single OR double OR treble OR triple))

## Appendix 7. Summary of search sources and returns

| **Database or Source Name** | **Items** |
| --- | --- |
| British Educational Research Database | 96 |
| Campbell Collaboration Systematic Reviews | 2 |
| Cochrane Controlled Trials Database | 22 |
| Directory of Open Access Journals (DOAJ) | 8 |
| EBSCO | 63 |
| ProQuest Education Database (Accessed via QUB Library) | 38 |
| Education Endowment Foundation | 4 |
| EPPI Centre Education Database | 2 |
| Google Scholar | 27 |
| Proquest International Bibliography of the Social Sciences (IBSS) (Accessed via QUB library) | 4 |
| Microsoft Academic | 14 |
| OECD iLibrary | 1 |
| OpenGrey (accessed via QUB library) | 1 |
| ProQuest dissertations (accessed via QUB library) | 16 |
| APA PsychInfo (Ovid) (accessed via QUB library) | 53 |
| Scientific Electronic Library Online (SciELO) (accessed via QUB library) | 1 |
| Scopus (Elsevier) (accessed via QUB library) | 83 |
| Web of science (Clarivate) (accessed via QUB library) | 74 |
| General reading | 100 |
| Full citation & reference search | 26 |
| Journal hand-search | 30 |
| References from author contact | 3 |
| **Total** | **668** |

## Appendix 8. Cohen’s K values for dual screening process

|  |  |  | Agree | | Disagree |  |  |
| --- | --- | --- | --- | --- | --- | --- | --- |
| Group # | Screening stage | Reviewer 1 | Include | Exclude | Include | Kappa k | Kappa rating |
| 1 | Full Text | Reviewer 1 | 6 | 25 | 2 | 0.615 | Substantial |
|  |  | Reviewer 2 |  |  | 3 |  |  |
| 2 | Full Text | Reviewer 1 | 3 | 18 | 7 | 0.0199 | Slight |
|  |  | Reviewer 2 |  |  | 7 |  |  |
| 3 & 4 | Full Text | Reviewer 1 | 9 | 19 | 2 | 0.449 | Moderate |
|  |  | Reviewer 2 |  |  | 8 |  |  |
| 1 | Title and Abstract | Reviewer 1 | 13 | 31 | 2 | 0.764 | Substantial |
|  |  | Reviewer 2 |  |  | 3 |  |  |
| 2 | Title and Abstract | Reviewer 1 | 8 | 30 | 3 | 0.445 | Moderate |
|  |  | Reviewer 2 |  |  | 8 |  |  |
| 3 | Title and Abstract | Reviewer 1 | 11 | 57 | 9 | 0.503 | Moderate |
|  |  | Reviewer 2 |  |  | 5 |  |  |
| 4 | Title and Abstract | Reviewer 1 | 8 | 34 | 2 | 0.604 | Substantial |
|  |  | Reviewer 2 |  |  | 5 |  |  |
| 5 | Title and Abstract | Reviewer 1 | 19 | 19 | 4 | 0.58 | Moderate |
|  |  | Reviewer 2 |  |  | 6 |  |  |
| 6 | Title and Abstract | Reviewer 1 | 11 | 23 | 0 | 0.43 | Moderate |
|  |  | Reviewer 2 |  |  | 14 |  |  |

## Appendix 9. Letter to authors to request unpublished research

Claire Dorris

School of Social Sciences, Education & Social Work

Queen’s University Belfast

20 College Green

Belfast, BT7 1HL

Northern Ireland

xx October 2020

To Whom it May Concern,

I am a final year student undertaking a Doctorate in Childhood Studies at Queen’s University, Belfast. My thesis will take the form of a systematic review and meta-analysis, registered with the Campbell Collaboration, on the use of mobile technology in primary/elementary schools to support literacy and/or numeracy outcomes for children. Co-authors on the review are Prof. Karen Winter and Dr. Liam O’Hare, Queen’s University, and Dr. Edda Tandi Lwoga, College of Business Education, Dar es Salaam.

I am in the process of conducting systematic searches, in line with robust systematic review methodology, to ensure that all relevant research, published and unpublished, is included. As part of this search process, I am contacting you as an individual or organisation with an interest in the topic, to ask if you are aware of any relevant research that may not be easily accessible through a database search strategy. This may include research in progress, unpublished articles, reports, dissertations, or other documentation.

Below, I have outlined the specific criteria for study inclusion in the review:

1. **Population**: The intervention must be delivered to children aged between 4-11 in a mainstream primary/elementary school class, within school hours. Studies which consider use of mobile devices in special schools, educational provision other than at school, afterschool programmes or for homework will not be included, nor will studies which focus on interventions to provide additional support to low-performing students, rather than the class as a whole.
2. **Intervention:** A mobile device is defined as a tablet computer, smartphone, handheld game console or other handheld device, with a touchscreen interface and internet connectivity. The intervention must involve pupils directly using the device, either individually or in pairs/groups, to support learning in literacy or numeracy. Interventions where only the teacher uses the device to support teaching will not be included.
3. **Outcome:** The study must report intervention effects for at least one outcome variable relating to children’s literacy or numeracy achievement.
4. **Study design:** The study must report effect sizes for the comparison of an intervention and control group or groups through a Randomised Control Trial (RCT) or Cluster RCT. Comparison interventions can include either:
   1. traditional teaching methods which do not incorporate technology (no intervention)
   2. an alternative technology (e.g. desktop computers)

I hope you agree that this systematic review will provide valuable evidence to support future policy and practice development in this rapidly evolving area of education, and thank you in advance for any additional information you can provide.

Yours sincerely,

Claire Dorris

**Email:** cadrain01@qub.ac.uk

## Appendix 10. Characteristics of included studies

|  | Authors | Year | Country | Literacy or numeracy | Age of pupils | % Boys | sample size | Screen size | Intervention Duration (hrs) | SAMR classification |
| --- | --- | --- | --- | --- | --- | --- | --- | --- | --- | --- |
| 1 | Bebell & Pedulla | 2015 | USA | Both | 5-6 | Unclear | 266 | ≥7 inch | Unclear | Unclear |
| 2 | Chen | 2014 | Scotland | Maths | 8-9 | 82% | 17 | <7 inch | 3.7 | Aug |
| 3 | Connor *et al.* | 2019 | USA | Literacy | 9.5 (mean) | 51% | 603 | ≥7 inch | 4.5 | Mod |
| 4 | Dundar & Akcayir | 2012 | Turkey | Literacy | 11-12 | Unclear | 20 | ≥7 inch | Unclear | Sub |
| 5 | Faber & Visscher | 2018 | Netherlands | Literacy | 8-9 | 49.1% | 1605 | ≥7 inch | Unclear | Mod |
| 6 | Faber, Luyten & Visscher | 2017 | Netherlands | Maths | 8-9 | 55% | 1808 | ≥7 inch | Unclear | Mod |
| 7 | Fabian and Topping | 2019 | Scotland | Maths | 10-11 | 50.5% | 74 | ≥7 inch | 40 | Mod |
| 8 | Hirotake, Kasai, Nishiuchi & Nakamuro | 2019 | Cambodia | Maths | 8.5 (mean) | Unclear | 1656 | ≥7 inch | 30 | Aug |
| 9 | Levesque, Bardack & Chigeda | 2020 | Malawi | Both | 6-10 | 53% | 674 | ≥7 inch | 120 | Mod |
| 10 | Messer, Thomas, Holliman & Kucirkova | 2018 | England | Maths | 5-6 | Unclear | 41 | ≥7 inch | 2 | Aug |
| 11 | Miller & Robertson | 2011 | Scotland | Maths | 10-11 | 43.6% | 634 | <7 inch | 15 | Aug |
| 12 | Outhwaite, Faulder, Gulliford and Pitchford | 2019 | England | Maths | 5.05 (mean) | 49.1% | 389 | ≥7 inch | 30 | Mod |
| 13 | Pitchford & Outhwaite | 2019 | Malawi | Maths | 8.27 | 48.8% | 153 | ≥7 inch | 20 | Mod |
| 14 | Pitchford | 2015 | Malawi | Maths | 7 | 65% | 204 | ≥7 inch | 20 or 10 | Mod |
| 15 | Pitchford, Chigeda, & Hubber | 2019 | Malawi | Literacy | 7-8 | 54.8% | 320 | ≥7 inch | 70 | Mod |
| 16 | Schacter & Jo | 2017 | USA | Maths | 4.59 (mean) | 50.3% | 433 | ≥7 inch | 7.3 | Mod |
| 17 | Sutherland *et al.* | 2019 | England | Maths | 8-10 | 50% | 2133 | ≥7 inch | Unclear | Mod |
| 18 | Yamac, Ozturk & Mutlu | 2020 | Turkey | Literacy | 10.12 (mean) | 44.8% | 96 | ≥7 inch | 12 | Red |

## Appendix 11. Characteristics of included interventions

| Study | Intervention summary |  |  | Intervention characteristics | | | | | | | | | |
| --- | --- | --- | --- | --- | --- | --- | --- | --- | --- | --- | --- | --- | --- |
|  |  | Significant effect found? | Dosage (hours) | Fun | Encourages autonomy | Repetitive learning | Creativity & combines skills | Provides formative feedback | Encourages collaboration | Adapts to individual ability | Teacher can monitor progress | Instructional element | SAMR rating (see 5.3.3 below) |
| Bebell and Pedulla (2015) | **1:1 iPad scheme:** Part of the Auburn Public Schools Advantage Programme, one of the world’s first district-wide 1:1 iPad provision schemes in education. Children are given 1:1 iPad access with the intention of supporting literacy and numeracy attainment- numerous apps are accessed, rather than the study testing just one. No consistent dosage in class so difficult to compare usage between schools. Control schools had no iPads. | One element only – hearing and recording sounds in words (not included in meta-analysis) | Unclear | ? | ? | ? | ? | ? | ? | ? | ? | ? | ? |
| Chen (2014) | **‘Brain Challenge' mobile phone game**: four different tasks were undertaken - logical, memory, visual and maths. Games ‘Trout Route’ and ‘Arithmetic’ were used as the intervention. Children played Trout Route five times before Arithmetic was unlocked. Each game allowed 90 seconds to answer the problems, and immediate feedback provided on correct/incorrect answers. Children completed a game record sheet to show how long they played and their high score each time. Control schools continued with class as normal. | No | 3.75 | X |  | X |  | X |  |  |  |  | Augmentation |
| Connor *et al.* (2019) | **Interactive e-book, read on a tablet:** the eBook was self-developed by the researchers specifically for tablet reading, with features including a 'choose your own adventure' theme, built in dictionary and comprehension or understanding tests with immediate feedback. Half of each experimental and control group also took part in a weekly 15-minute book club to discuss their reading and strategy (not included in meta-analysis). Control groups read the same text in traditional printed form, without the additional features. | Yes, for one element only - word knowledge | 4.5 | X | X |  | X | X |  |  |  |  | Moderation |
| Dundar and Akcayir (2012) | **eBook:** Children read aloud to a teacher or researcher, either from a text on an iPad or a traditional printed book. Text was identical. The teacher/researcher noted errors. No additional features were included in the experimental group intervention. | No | Unclear |  |  |  |  |  |  |  |  |  | Substitution |
| Faber and Visscher (2018) | **Snappet, an online teaching environment:** school used Snappet for 6 months to work on spelling, while control group continued with business as usual. Pupils complete set assignments aligned to the curriculum, and additionally, the app sets 'adaptive' assignments based on their performance. They receive immediate basic feedback (i.e. if right or wrong). Teachers can also review scores and adjust assignments to meet the needs of individuals or the class. | No | Unclear |  | X |  |  | X |  | X | X |  | Modification |
| Faber, Luyten and Visscher (2017) | **Snappet, an online teaching environment:** Schools used Snappet for 6 months to work on spelling, while control group continued with business as usual. Pupils complete set assignments aligned to the curriculum, and additionally, the app sets 'adaptive' assignments based on their performance. They receive immediate basic feedback (i.e. if right or wrong). Teachers can also review scores and adjust assignments to meet the needs of individuals or the class. | Yes. Effect more pronounced for high-achieving students | Unclear |  | X |  |  | X |  | X | X |  | Modification |
| Fabian and Topping (2019) | **Mobile apps (Skitch, Pixel Touch, Measure Map and Area and Perimeter):** Children worked in pairs and used a combination of mobile apps to undertake maths activities. Pupils used apps Skitch and Pixel touch to photograph objects and calculate angle sizes of real objects, and Measure Map and Area and Perimeter, to calculate areas. They also had to look for shapes in the natural environment with specific geometric properties. Children were able to go out of the classroom to take pictures and measure objects. The control group undertook the same activities but used paper and pencil to draw objects rather than photograph. | No | 40 | X | X |  | X |  | X |  |  |  | Modification |
| Hirotake *et al.* (2019) | **‘Think Think’ app:** 100 fun mini games to support maths learning. Children play the games for a maximum 10 minutes per day to keep it fresh and fun and help children stay motivated to use it. The difficulty of the games adapts to match the child’s ability. The control group continued with usual class activities. | Yes | 30 | X | X | X |  |  |  | X |  |  | Augmentation |
| Levesque, Bardack and Chigeda (2020) | **onecourse app (part of the Onebillion programme):** a maths and literacy app with 1248 instructional literacy units and 282 maths units. The activities are interactive and game-based, and children can work at their own pace, receiving immediate feedback on their performance as they go. Teachers can track progress and step in to provide immediate support if needed. | Yes | 120 | X | X | X |  | X |  | X | X | X | Modification |
| Messer *et al.* (2018) | **Bee-bot app for iPad:** children input instructions to help a bee reach a flower through a maze (e.g. forward, back, left turn 90 degrees, right turn 90 degrees). There are eighteen progressively difficult levels available, with a timer and stars awarded for successful completion. The control group undertook a paper and pencil version, using printed screen shots on which children wrote or drew the commands. | No | 2 | X | X | X |  |  |  | X |  |  | Augmentation |
| Miller and Robertson (2011) | **Dr Kawashima's Brain Training:** a series of fun mini games played individually on a Nintendo DS lite. This is a commercial game, and while details not given in the paper on specific activities, the game provides ‘brain age’ and performance feedback and unlocks new games the longer it is played. | Yes – speed and accuracy of completion | 15 | X | X | X |  |  |  |  |  |  | Augmentation |
| Outhwaite *et al.* (2019) | **One Billion maths app:** Children individually work through fun, age-appropriate maths activities (aligned to EYFS curriculum) on topics such as number, shape, space, measures. Online ‘teachers’ provide instruction, and children can ask them to repeat if they don’t understand. Touch screen function is used so that tasks are manipulative, and children receive immediate feedback on their performance. Activities also adapt to the child’s progress. An in-app quiz assesses progress. | Yes | 30 | X | X | X |  | X |  | X | X | X | Moderation |
| Pitchford and Outhwaite (2019) | **onebillion maths app (as above)**: Translated to local language. | Yes | 30 | X | X | X |  | X |  | X | X | X | Moderation |
| Pitchford, Chigeda and Hubber (2019) | **onebillion reading app:** Reading intervention with similar functionality to maths app. Content is based on the local curriculum, activities are fun and interactive, and children work alone at their own pace, receiving feedback on performance and with in-app teaching instruction. | Yes | 70 | X | X | X |  | X |  | X | X | X | Moderation |
| Pitchford, N. (2015) | **onebillion Maths app (as above):** translated into local language. | Yes – for some elements of literacy | 20  / 10 | X | X | X |  | X |  | X | X | X | Moderation |
| Schacter & Jo (2017) | **Maths Shelf:** A maths app for preschool and year 1 pupils (USA therefore primary age equivalent), which is based on Montessori maths learning theory and wider child development theories. Children first take a ‘placement test’ to identify starting point relevant to their current skill level, then work through various activities at their own speed. Activities are game-based and fun, provide immediate feedback on progress, and involve manipulative and ‘hands on’ activities using the touchscreen function. | Yes | 7.3 | X | X | X |  | X |  | X | ? |  | Moderation |
| Sutherland *et al.* (2019) | **Explain Everything app:** An app specifically for teachers to provide digital feedback on pupils’ maths work. Teachers record their feedback via an app, either using video, voice recording, or annotations on digital work. Pupils then access this feedback individually on their tablet. Pupils can listen/watch the feedback during class as many times as they need to grasp the message. | No | Unclear |  |  |  |  | X |  |  |  |  | Modification |
| Yamac, Ozturk and Mutlu (2020) | **Strategic Digital Writing Environment (SADIWE):** A creative writing app developed specifically for this research. Teacher and pupils access the app via individual tablets. The teacher sets writing assignments, and the SADIWE app takes pupils through the stages of writing, including planning, idea generation, and organisation, and includes video instruction. Pupils work on their own device but can provide feedback to others. On completion, pupils add images and upload their piece to the class blog. The control group worked on the same assignment, but using paper and pencil, with group work completed face to face. | Yes – for quality and writing knowledge | 12 | X | X |  | X | X | X |  | X | X | Redefinition |

## Appendix 12. Summary of outcome measures assessed in included studies

|  | **Study ID** | **Subject** | **Outcome domain** | **Instrument** | **Standardised (S) or designed for the research (D)** | **Administration** | **Validity/ Reliability** |
| --- | --- | --- | --- | --- | --- | --- | --- |
| 1 | Bebell & Pedulla, 2015 | Literacy | Accuracy, comprehension, and fluency | Rigby Benchmark Assessment | S | Not specified but usually relies on observation by teacher. | All standardised tests administered regularly and shown to be valid and reliable tests of literacy development. |
|  |  | Literacy | Phonics/writing | Children’s Progress Academic Assessment (CPAA) | S |  |  |
|  |  | Literacy | Listening | CPAA | S |  |  |
|  |  | Literacy | Phonemic Awareness | CPAA | S |  |  |
|  |  | Literacy | Reading | CPAA | S |  |  |
|  |  | Literacy | Letter Identification | Observation Survey of Early Literacy Achievement (OSELA) | S |  |  |
|  |  | Literacy | Concepts about Print | OSELA | S |  |  |
|  |  | Literacy | Word Reading/Ohio Word Test | OSELA | S |  |  |
|  |  | Literacy | Writing Vocabulary | OSELA | S |  |  |
|  |  | Literacy | Hearing/Recording work Sounds | OSELA | S |  |  |
| 2 | Chen, 2014 | Numeracy | number attempted | Mental Maths Challenge test | D | Paper test | No discussion on reliability/ validity of assessment. |
|  |  | Numeracy | number correct answers | Mental Maths Challenge test | D |  |  |
|  |  | Numeracy | accuracy rate | Mental Maths Challenge test | D |  |  |
| 3 | Connor *et al*. 2019 | Literacy | Word knowledge | Gates MacGinitie Reading Test | S | Paper test | Published reliability score of 0.96 |
| 4 | Dundar and Akcayir, 2012 | Literacy | Reading comprehension | Akyol’s understanding chart | S | Read aloud to teacher from paper or tablet text.  Both paper and digital versions available- unclear which was used. | No discussion on reliability or validity. |
|  |  | Literacy | Reading speed | Timed by teacher | D |  |  |
| 5 | Faber and Visscher, 2018 | Literacy | Spelling ability | Cito Standardised spelling test | S |  | Published detail on high validity and reliability of CITO tests. |
| 6 | Faber *et al.* 2017 | Numeracy | Composite maths ability | Cito Standardised maths test | S | As above | As above |
| 7 | Fabian and Topping, 2019 | Numeracy | Geometry (symmetry, angles, area) | Bespoke maths test | D | Test items taken from standard textbooks. | Experienced maths teacher revied test items as validity check. |
| 8 | Hirotake *et al.* 2019 | Numeracy | Composite maths ability | National Achievement Test | S | Not clear | National tests widely regarded as valid and reliable tests. |
|  |  | Numeracy | Composite maths ability | Trends in International Maths and Science Study (TIMMS) | S | Not clear |  |
|  |  | Numeracy | IQ | Tanaka-B IQ test | S | Not clear |  |
| 9 | Levesque *et al.* 2020 | Numeracy | Composite maths ability | Early Grade Maths Assessment | S | Collected on device by assessor | Cronbach’s Alpha calculated for all measures (0.75 – 0.85 average – acceptable to good). |
|  |  | Literacy | Competency literacy ability | Early Grade Reading Assessment | S |  |  |
|  |  | Literacy | reading fluency- non-words | Early Grade Reading Assessment | S |  |  |
|  |  | Literacy | reading fluency - words | Early Grade Reading Assessment | S |  |  |
| 10 | Messer *et al.* 2018 | Numeracy | Maths ability | Based on British Ability Scales Number subtest | D | Not clear | Cronbach’s Alpha calculated for internal reliability (0.84 – 0.88 - good). |
|  |  | Numeracy | Spatial awareness | Based on British Ability Scales Number subtest | D |  |  |
|  |  | Numeracy | Working memory | Based on British Ability Scales Number subtest | D |  |  |
| 11 | Miller and Robertson, 2011 | Numeracy | Maths ability | 100-item maths 'number challenge' test | D | Not clear | Experienced maths teacher revied test items as validity check. |
|  |  | Numeracy | Time taken | 100-item maths 'number challenge' test | D |  |  |
| 12 | Outhwaite *et al.* 2019 | Numeracy | Maths ability | Progress Test in Maths (PTM5) assessment | S | Paper-based | Highly regarded standardised assessment, Cronbach’s alpha calculated for internal reliability (0.80 - good). |
|  |  | Numeracy | Fluency in facts | Progress Test in Maths (PTM5) assessment | S |  |  |
|  |  | Numeracy | Fluency in concepts | Progress Test in Maths (PTM5) assessment | S |  |  |
|  |  | Numeracy | Mathematical reasoning | Progress Test in Maths (PTM5) assessment | S |  |  |
|  |  | Numeracy | Problem solving | Progress Test in Maths (PTM5) assessment | S |  |  |
| 13 | Pitchford and Outhwaite, 2019 | Numeracy | Maths knowledge | Maths test based on curriculum | D | Child completed on iPad | Validity and reliability of tool assessed, and paper published. |
| 14 | Pitchford, 2015 | Numeracy | Mathematical concepts | Maths test based on curriculum | D | iPad |  |
|  |  | Numeracy | Curriculum maths knowledge | Maths test based on curriculum | D | iPad |  |
|  |  | Numeracy | Maths curriculum knowledge generalization | Maths test based on curriculum | D | iPad |  |
| 15 | Pitchford *et al.* 2019 | Literacy | Composite literacy ability | Early Grade Reading Assessment | D | Collected by assessor on iPad | Established validity. |
| 16 | Schacter & Jo, 2017 | Numeracy | Composite maths ability | Early Maths Assessment | D | iPad | Cronbach’s Alpha for internal reliability – 0.93 (excellent). |
| 17 | Sutherland *et al.* 2019 | Literacy | Composite literacy skills | ACER Essential Learning Metric | S | Paper-based | Widely accepted valid and reliable assessment with evidence base. |
| 18 | Yamac *et al.* 2020 | Literacy | Writing quality | Assessment of written essay | D | Written | Cronbach’s alpha for internal consistency calculated (0.91 - excellent), interrater reliability (0.88). |
|  |  | Literacy | Number of words | Assessment of written essay | D | Written |  |
|  |  | Literacy | Writing knowledge | Assessment of written essay | D | Written |  |

## Appendix 13. Coding script for RStudio

**Analysis 1: All studies**

**## open package readxl**

> library(readxl)

**## read in data file**

> dat <- read_excel("data/SMD all studies.xlsx")

**## display vioplot to identify outliers**

vioplot(SMD_all_studies$yi, col = "blue", names = "learning outcomes")

**## fit RVE random-effects model with correlated effects weights**

> res2 <- robu(formula = yi ~ 1, var.eff.size = vi, studynum = studyid, modelweights = c("CORR", "HIER"), rho = 0.8, small = TRUE, data = SMD_all_studies)

>Print(res2)

**## conduct sensitivity analysis to determine effect of value of Tau used, as recommended by Hedges *et al.* (2010)**

> sensitivity(res2)

**## draw forest plot**

> forest.robu(res2, es.lab = "Outcome_measure", study.lab = "Authors", "Effect Size" = effect.size, "weight" = r.weights)

**## fit meta-regression model using hierarchical effects weighting**

> model_hier <- robu(yi ~ CC1+CC2+CC3+CC4+CC5+CC6, data = SMD_all_studies, modelweights = "HIER", studynum = studyid, var.eff.size = vi, small = TRUE)

**## publication bias analysis**

> pubbi 2<- rma(yi, vi = vi, data = SMD_all_studies)

> trimfill(pubbi2)

**## draw funnel plot**

funnel(pubbi2, refline = 0)

**## classical Egger test**

> regtest(pubbi2, model = "lm")

**## random/ mixed effects Egger test**

> regtest(pubbi2)

> Trimfull(Pubbi2)

**Analysis 2: removing high risk studies**

**## display vioplot to identify outliers**

vioplot(SMD_minus_high_risk2$yi, col = "blue", names = "learning outcomes")

**## fit RVE random-effects model with correlated effects weights**

> res3 <- robu(formula = yi ~ 1, var.eff.size = vi, studynum = studyid, modelweights = c("CORR", "HIER"), rho = 0.8, small = TRUE, data = SMD_minus_high_risk2)

**## sensitivity analysis for differing values of Rho**

> sensitivity(res3)

**## Draw forest plot**

>forest.robu(res3, es.lab = "Outcome_measure", study.lab = "Authors", "Effect Size" = effect.size, "weight" = r.weights)

Hierarchical model not run as less outcomes than previously (df already less than 4)

**## publication bias analysis**

pubbi3<- rma(yi, vi, data = SMD_minus_high_risk2)

trimfill(pubbi3)

**## draw funnel plot & inspect for symmetry**

> funnel(pubbi3, refline = 0)

**## Classical Egger Test**

> regtest(pubbi3, model = "lm")

**## Random/ mixed effects Egger test**

regtest(pubbi3)

## Appendix 14. Full outcome data extracted, including effect size and variation calculations

| studyid | Authors | Year | Outcome_measure | m1i | sd1i | n1i | m2i | sd2i | n2i | CC1 | CC2 | CC3 | CC4 | CC5 | CC6 | yi | vi |
| --- | --- | --- | --- | --- | --- | --- | --- | --- | --- | --- | --- | --- | --- | --- | --- | --- | --- |
| 1 | Chen | 2014 | number_attempted | 46.33 | 14.81 | 9 | 53.25 | 7.59 | 8 | 1 | 2 | 1 | 2 | 2 | 2 | -0.54752 | 0.244928 |
| 1 | Chen | 2014 | number_correct_answers | 41.89 | 14.06 | 9 | 46.25 | 6.48 | 8 | 1 | 2 | 1 | 2 | 2 | 2 | -0.37005 | 0.240139 |
| 1 | Chen | 2014 | accuracy_rate | 89 | 7.76 | 9 | 87 | 2.59 | 8 | 1 | 2 | 1 | 2 | 2 | 2 | 0.319699 | 0.239117 |
| 2 | Connor et al | 2019 | Word_knowledge | 12.59 | 5.63 | 200 | 11.81 | 4.48 | 103 | 2 | 3 | 1 | 2 | 1 | 1 | 0.147681 | 0.014745 |
| 3 | Dundar & Akcayir | 2012 | Reading_comprehension | 5.16 | 0.27 | 10 | 4.93 | 0.31 | 10 | 2 | 1 |  | 3 |  | 1 | 0.757713 | 0.214353 |
| 4 | Faber & Visscher | 2018 | Cito_Standardised_spelling_test | 132.1 | 7.65 | 619 | 131.8 | 7.52 | 963 | 2 | 3 |  | 2 | 1 | 1 | 0.039605 | 0.002654 |
| 5 | Faber, Luyten & Visscher | 2017 | Cito_standardised_maths_test | 83 | 13.2 | 803 | 81.7 | 12.2 | 971 | 2 | 3 |  | 2 | 1 | 2 | 0.102623 | 0.002278 |
| 6 | Fabian and Topping | 2019 | Maths_test_MT | 20.57 | 6.3 | 35 | 19.72 | 5.7 | 39 | 2 | 3 | 4 | 3 | 1 | 2 | 0.1404 | 0.054346 |
| 7 | Levesque, Bardack & Chigeda | 2020 | Early_Grade_Maths_Assessment | 0.34 | 0.189 | 199 | 0.32 | 0.177 | 187 | 2 | 3 | 5 | 2 | 1 | 2 | 0.108906 | 0.010388 |
| 7 | Levesque, Bardack & Chigeda | 2020 | Early_Grade_Reading_Assessment_composite_score | 0.19 | 0.109 | 199 | 0.16 | 0.1 | 187 | 2 | 3 | 5 | 2 | 1 | 1 | 0.285871 | 0.010479 |
| 7 | Levesque, Bardack & Chigeda | 2020 | reading_fluency_nonwords | 3.03 | 6.85 | 199 | 2.07 | 5.78 | 187 | 2 | 3 | 5 | 2 | 1 | 1 | 0.150784 | 0.010402 |
| 7 | Levesque, Bardack & Chigeda | 2020 | reading_fluency_words | 3.94 | 9.23 | 199 | 2.59 | 7.23 | 187 | 2 | 3 | 5 | 2 | 1 | 1 | 0.161913 | 0.010407 |
| 8 | Messer, Thomas, Holliman & Kucirkova | 2018 | Maths | 6.14 | 3.88 | 14 | 7.43 | 4.03 | 14 | 2 | 2 | 1 | 1 |  | 2 | -0.3166 | 0.144647 |
| 8 | Messer, Thomas, Holliman & Kucirkova | 2018 | Spatial_awareness | 11.93 | 4.1 | 14 | 12.5 | 4.03 | 14 | 2 | 2 | 1 | 1 |  | 2 | -0.13613 | 0.143188 |
| 8 | Messer, Thomas, Holliman & Kucirkova | 2018 | working_memory | 8.57 | 2.47 | 14 | 9.43 | 3.01 | 14 | 2 | 2 | 1 | 1 |  | 2 | -0.30324 | 0.144499 |
| 9 | Miller & Robertson | 2011 | maths_'number_challenge'_test | 83.04 | 16.86 | 326 | 81.65 | 20.8 | 309 | 1 | 2 | 2 | 3 | 3 | 2 | 0.073535 | 0.006308 |
| 10 | Outhwaite, Faulder, Gulliford and Pitchford | 2019 | PTM5_maths_ability_assessment | 15.02 | 5.08 | 131 | 15.09 | 4.82 | 132 | 2 | 3 | 3 | 1 | 1 | 2 | -0.0141 | 0.01521 |
| 10 | Outhwaite, Faulder, Gulliford and Pitchford | 2019 | Fluency_in_facts | 3.68 | 2.06 | 131 | 3.64 | 1.95 | 132 | 2 | 3 | 3 | 1 | 1 | 2 | 0.019887 | 0.01521 |
| 10 | Outhwaite, Faulder, Gulliford and Pitchford | 2019 | Fluency_in_concepts | 6.78 | 1.81 | 131 | 6.76 | 1.81 | 132 | 2 | 3 | 3 | 1 | 1 | 2 | 0.011018 | 0.01521 |
| 10 | Outhwaite, Faulder, Gulliford and Pitchford | 2019 | Mathematical_reasoning | 3.49 | 1.72 | 131 | 3.65 | 1.69 | 132 | 2 | 3 | 3 | 1 | 1 | 2 | -0.09357 | 0.015226 |
| 10 | Outhwaite, Faulder, Gulliford and Pitchford | 2019 | Problem_solving | 1.07 | 0.91 | 131 | 1.05 | 0.82 | 132 | 2 | 3 | 3 | 1 | 1 | 2 | 0.023028 | 0.01521 |
| 11 | Pitchford & Outhwaite | 2019 | mathematics | 44.84 | 16.37 | 76 | 32.22 | 15.51 | 77 | 2 | 3 | 2 | 2 | 1 | 2 | 0.787633 | 0.028172 |
| 12 | Pitchford, N. | 2015 | S1_maths_concepts | 5.1 | 4.6 | 22 | 4.5 | 4.3 | 20 | 2 | 3 | 1 | 1 | 2 | 2 | 0.131988 | 0.095662 |
| 12 | Pitchford, N. | 2015 | S1_curriculum_maths_knowledge | 7.7 | 6.3 | 22 | 5.9 | 5.3 | 20 | 2 | 3 | 1 | 1 | 2 | 2 | 0.302068 | 0.096541 |
| 12 | Pitchford, N. | 2015 | S2_maths_concepts | 14.6 | 6.6 | 38 | 10.5 | 6.5 | 37 | 2 | 3 | 2 | 2 | 2 | 2 | 0.619415 | 0.055901 |
| 12 | Pitchford, N. | 2015 | S2_curriculum_maths_knowledge | 20.7 | 10.3 | 38 | 10.8 | 7.4 | 37 | 2 | 3 | 2 | 2 | 2 | 2 | 1.090158 | 0.061266 |
| 12 | Pitchford, N. | 2015 | S2_maths_knowledge_generalization | 23.8 | 9.1 | 38 | 20.7 | 8.4 | 37 | 2 | 3 | 2 | 2 | 2 | 2 | 0.35016 | 0.05416 |
| 12 | Pitchford, N. | 2015 | S3_maths_concepts | 19.5 | 5.2 | 44 | 18.6 | 6 | 43 | 2 | 3 | 2 | 2 | 2 | 2 | 0.15902 | 0.046128 |
| 12 | Pitchford, N. | 2015 | S3_curriculum_maths_knowledge | 35.2 | 7 | 44 | 23.4 | 6.9 | 43 | 2 | 3 | 2 | 2 | 2 | 2 | 1.682623 | 0.062254 |
| 12 | Pitchford, N. | 2015 | S3_maths_knowledge_generalization | 33.5 | 7.3 | 44 | 27.2 | 8.1 | 43 | 2 | 3 | 2 | 2 | 2 | 2 | 0.81034 | 0.049757 |
| 13 | Pitchford, N, Chigeda, A & Hubber, P | 2019 | EGRA_all | 18.99 | 20.94 | 162 | 13.96 | 17.34 | 158 | 2 | 3 | 5 | 2 | 1 | 1 | 0.260723 | 0.012608 |
| 14 | Schacter | 2017 | Early_Maths_Assessment_total_score | 30.24 | 8.85 | 260 | 21.92 | 8.85 | 173 | 2 | 2 | 1 | 1 | 1 | 2 | 0.938476 | 0.010644 |
| 15 | Sutherland et al | 2019 | ACER_Essential_Learning_Metric_ELM | 18.4 | 5.93 | 1103 | 19.7 | 6.55 | 1030 | 2 | 2 |  | 2 | 1 | 2 | -0.20836 | 0.001888 |
| 16 | Yamac, Ozturk & Mutlu | 2020 | Writing_quality_WQ | 14.97 | 1.68 | 49 | 14.08 | 1.71 | 47 | 2 | 4 | 2 | 3 | 3 | 1 | 0.520949 | 0.043098 |
| 16 | Yamac, Ozturk & Mutlu | 2020 | Number_of_words_NW | 150.62 | 57.61 | 49 | 127.5 | 57.66 | 47 | 2 | 4 | 2 | 3 | 3 | 1 | 0.397938 | 0.04251 |
| 16 | Yamac, Ozturk & Mutlu | 2020 | Writing_knowledge_WK | 10.01 | 3.64 | 49 | 6.71 | 3.63 | 47 | 2 | 4 | 2 | 3 | 3 | 1 | 0.900547 | 0.045909 |
| 17 | Ito, Kasai, Nishiuchi & Nakamuro | 2019 | National_Achievement_Test_Grade_3_class | |  | 177 |  |  | 179 | 2 | 2 | 3 | 3 |  | 2 | 0.1489 | 0.0113 |
| 17 | Ito, Kasai, Nishiuchi & Nakamuro | 2019 | TIMSS_Grade_4_class |  |  | 174 |  |  | 173 | 2 | 2 | 3 | 3 |  | 2 | 0.209 | 0.0116 |
| 17 | Ito, Kasai, Nishiuchi & Nakamuro | 2019 | TanakaB_IQ_test_Grades_1_&_2 |  |  | 700 |  |  | 685 | 2 | 2 | 3 | 3 |  | 2 | 0.1328 | 0.0029 |
| 18 | Bebell & Pedulla | 2015 | OSELA:Hearing and Recording Sounds in Words (HRSIW) | | | 129 |  |  | 137 | 2 |  |  | 1 |  | 2 | 0.2895 | 0.0152 |

## Appendix 15. Moderator variables and codes

| **Code** | **Moderator variable** | **Assigned values** |
| --- | --- | --- |
| CCI | screen size | 1= <7 inches  2 = 7 inches or larger |
| CC2 | SAMR (model on levels of implementation) | 1 = substitution  2 = augmentation  3 = modification  4 = redefinition |
| CC3 | intervention dosage | 1=1-10 hours  2=11-20 hours  3=21-30 hours  4=31-40 hours  5=41+ hours |
| CC4 | age | 1= age 4 – 6  2 = age 7 – 9  3 = age 10 – 12 |
| CC5 | gender breakdown | 1 = equal numbers of boys & girls (within 2%)  2 = higher % boys  3 = higher % girls |
| CC6 | subject | 1= numeracy  2 = literacy |

## Appendix 16. Request for missing data from authors to support meta-analysis

Dear xxxx

I am a final year Doctoral student, working on a systematic review and meta-analysis, registered with the Campbell Collaboration, as follows: ‘A systematic review of mobile device use in the primary school classroom and its impact on pupil literacy and numeracy attainment’.

The search and screening process has identified your paper ‘[INSERT TITLE (author, date)] as a relevant paper for inclusion.

I’m in the process of extracting outcome data for analysis, and hoped you would be able to clarify the following information:

[insert details of data required]

Your support would be very much appreciated to ensure completeness of the review’s findings, and I hope you agree that this is a useful study with important implications for practice.

Best wishes,

Claire

Claire Dorris

DChild Research Student

School of Social Sciences, Education & Social Work Queen’s University Belfast, Northern Ireland

## Appendix 17. Data extraction/coding framework

The following tool was adapted from guidelines set out in ‘Chapter Five from the Cochrane Handbook for Systematic Reviews of Interventions. The tool was piloted and refined, and uploaded to EPPI-Reviewer for final data extraction.

| General Information | |
| --- | --- |
| Name of data extractor: |  |
| Date of data extraction |  |
| Study ID or reference |  |
| Report ID (if more than one report relating to the same study) |  |
| If linked, IDs of linked reports |  |
| Author/s |  |
| Lead author contact details |  |
| Year of publication |  |
| Publication type (e.g. journal) | Journal article  Technical report  Dissertation/thesis  Unpublished study  Other (specify) |
| Citation |  |
| Study funder |  |
| Is author affiliated with funder? | Y/N/Unclear |
| Study methodology | |
| Aim of the study |  |
| Study design (e.g. cluster randomised or RCT) |  |
| Method of recruitment |  |
| Sampling procedures |  |
| Inclusion/exclusion criteria applied |  |
| Unit of allocation (e.g. individual, class, school) |  |
| Start date of study |  |
| End date of study |  |
| Duration of participation (from recruitment to last follow-up) |  |

| Participants: | | |
| --- | --- | --- |
| Population description |  | |
| Country/region of study |  | |
| Setting |  | |
| Method of recruitment of settings |  | |
| Inclusion criteria |  | |
| Exclusion criteria |  | |
| Total number of participants: |  | |
| - At the beginning |  | |
| - At the end |  | |
| - % Participants who completed the study |  | |
| - Details of attrition at any stage of the study |  | |
| Age (SD, mean, range):   - For overall sample - For each group reported |  | |
| Class/classes/grade reported |  | |
| Gender   - Of overall sample - For each group | Report # and % | |
| Any other relevant characteristics specified? |  | |
| Was participation voluntary (for children) | Y/N/Unclear | |
| Consent reported? | Y/N/Unclear | |
| If yes, who was consent sought from? (Select all that apply) | Child  Parent  Teacher  Other (specify) | |
| Method of consent (for each of the above) | Opt in  Opt out | |
| Intervention: | | |
| Name of the intervention | |  |
| Description of intervention, target outcome/s & key activities | |  |
| Technology device used | | Tablet  Smartphone  Handheld game console  Other (specify)  Screen size (specify) |
| Who owed the devices used? | | School  Pupils  Combination  Unclear |
| How was the intervention accessed? | | App downloaded to the device  Website accessed online  Hardware already on the device  Other (please specify) |
| Individual device use or groups? | | Individual use  In pairs  Groups (3 or more)  Unclear (specify) |
| Dosage of intervention (frequency and length of sessions, total number of hours | |  |
| Cost of accessing the intervention (if available) | |  |
| Who delivered or supervised the intervention (e.g. teacher, classroom assistant) | | Teacher  Classroom assistant  Other (specify) |
| What was their role? | |  |
| Was specialist training required to deliver the intervention? | | Y/N/Unclear  (If yes, provide detail) |
| What is the primary aim/target outcome of the intervention? | |  |
| What is/are the secondary aims/outcomes of the intervention? | |  |
| Number of intervention groups | |  |
| Number of control groups | |  |
| Any differences found in baseline between the groups? | |  |
| Provide details of the control/comparison condition | | Business as usual  Other intervention  Waiting list  Other? |
| Were any other interventions being delivered concurrently which may have impacted outcomes? | | Y/N/unclear |
| If yes, provide detail | |  |
| Was effort made to assess fidelity? | | Y/N/Unclear |
| If yes, provide detail | |  |

| Assessment of level of intervention on the SAMR model | |
| --- | --- |
| **Substitution:** technology replaces a traditional activity, but activity remains fundamentally the same |  |
| **Augmentation:** technology replaces the traditional activity and adds some additional function |  |
| **Modification:** Technology significantly alters the original task |  |
| **Redefinition:** technology allows task to be redefined to include previously unachievable activities |  |
| Outcomes | |
| Outcomes assessed | Academic achievement:  Literacy (specify)  Numeracy (specify)  Other (specify)  Unclear (specify) |
| Answer the following questions for each outcome assessed: |  |
| Measurement tools used and details of administration |  |
| Unit of measurement (if relevant) |  |
| Were tools validated? | Y/N/unclear  (Provide detail) |
| Time points at which measures were assessed | After intervention only  Before and after intervention  Unclear (specify) |
| How long after intervention was data collected? | Between 0-3 months  Between 4-6 months  Between 6-9 months  Longer than 9 months (specify)  Unclear (specify) |
| Was follow up data collected? | Yes (specify time points)  No  Unclear |
| Summary data collected |  |
| Missing data? | Y/N/unclear  Provide detail |
| Action taken to access summary data (if not provided) | (If no above) |
| Statistical analyses conducted |  |

| Findings | |
| --- | --- |
| Key conclusions of authors |  |
| References to other relevant studies identified: |  |
| Details on further correspondence needed: (who with, what information is needed, when/how will it be requested) |  |

(Repeat this table for each outcome)

| Data collection and analysis | | | | |
| --- | --- | --- | --- | --- |
| Comparison |  | | | |
| Outcome measure |  | | | |
| Type of outcome (e.g. dichotomous, continuous) |  | | | |
| Subgroup |  | | | |
| Time point of data collection (specify from start or end of intervention) |  | | | |
| Post-intervention or change from baseline? (If continuous) |  | | | |
| No of participants | Intervention | | Control | |
|  |  | |  | |
| Results (if dichotomous) | Intervention | | Comparison | |
|  | No. with event | Total in group | No. with event | Total in group |
|  |  |  |  |  |
| Results (if continuous) | Intervention | | Control | |
|  | Mean | SE (or another variance) | Mean | SE (or another variance) |
|  |  |  |  |  |
|  | Overall effect size | | Variance | |
|  |  | |  | |
| Any other results reported (e.g. odds ratio, risk difference, CI or P value) |  | | | |
| No. missing participants |  | |  | |
| Reasons missing |  | |  | |
| Unit of analysis (by individuals, cluster/groups) |  | | | |
| Statistical methods used and appropriateness of these *(e.g. adjustment for correlation)* |  | | | |
